# Quantifying the mRNA epitranscriptome reveals epitranscriptome signatures and roles in cancer

**DOI:** 10.1007/s00018-025-05805-7

**Published:** 2025-07-28

**Authors:** Ying Feng, Xiaoli He, Mingxin Guo, Ying Tang, Guantong Qi, Qian Huang, Wenran Ma, Hong Chen, Yifan Qin, Ruiqi Li, Jin Wang, Yu Liu

**Affiliations:** State Key Laboratory of Reproductive Regulation and Breeding of Grassland Livestock, Institute of Biomedical Sciences, School of Life Sciences, Inner Mongolia University, Hohhot, People’s Republic of China

**Keywords:** mRQuant, RNA modification, Cancer, Drug resistance, m^1^A

## Abstract

Post-transcriptional modifications on mRNA are crucial for mRNA fate and function. The current lack of a comprehensive method for high-coverage and sensitive quantitative analysis of mRNA modifications significantly limits the discovery of new mRNA modifications and understanding mRNA modifications’ occurrence, dynamics and function. Here, a highly sensitive, high-throughput and robust LC-MS/MS-based technique, mRQuant, was developed to simultaneously detect and quantify 84 modified ribonucleosides in cellular mRNA. Using mRQuant, we quantified 32–34 modified ribonucleosides across several human cancer and non-cancer cell lines and uncovered cancer- and cancer type-specific signatures. Analyses of cisplatin- and paclitaxel-treated HeLa cells and drug-resistant variants revealed several drug resistance-associated modifications. Among them, m^1^A exhibited significant differences across multiple cell types and between cancerous and non-cancerous cells. Knocking down mRNA m^1^A writer or eraser protein resulted in altered cell viability, cell cycle and apoptosis in HeLa cells, suggesting a role of mRNA m^1^A in cancer. Transcriptomic and proteomic analyses further revealed the molecular mechanisms underlying the phenotypic variation.

**Supplementary information:**

The online version contains supplementary material available at 10.1007/s00018-025-05805-7.

## Introduction

Beyond the four classic nucleosides (adenosine, guanosine, cytidine, uridine), RNA molecules also contain many chemical modifications [1]. To date, over 170 different types of RNA modifications have been reported to be present in nature [2] most of which are found in the abundant noncoding RNAs, i.e., transfer RNA (tRNA) and ribosomal RNA (rRNA) [3]. The discovery of *N* ^6^-methyladenosine (m^6^A) and other reversible modifications in mRNA opens new directions in RNA modification-mediated regulation of gene expression and other biological processes [4].

With the advancement of detection technologies, a handful of mRNA modifications have been characterized in mammals, including m^6^A, *N*^6^,2’-*O*-dimethyladenosine (m^6^Am), *N*^7^-methylguanosine (m^7^G), *N*^1^-methyladenosine (m^1^A), pseudouridine (ψ), 5-methyluridine (m^5^U), 5-methylcytidine (m^5^C), *N*^3^-methylcytidine (m^3^C), 5-hydroxymethylcytidine (hm^5^C), *N*^4^-acetylcytidine (ac^4^C), inosine (I), and 2’-*O*-methylation (Nm) [5–8]. Previous studies have demonstrated that these modifications play important roles in RNA metabolism and function, immunity, development and disease [9–11].

A variety of techniques have been developed to measure RNA modifications, such as liquid chromatography-tandem mass spectrometry (LC-MS/MS) [12–14], epitranscriptomic sequencing [15, 16] and capillary electrophoresis with MS detection [17, 18]. The LC-MS/MS technology has been and remains the ‘gold standard’ for RNA modification analysis, because it enables simultaneous analysis of many modifications with high sensitivity, high specificity, and high accuracy and direct measurement of RNA [19, 20]. However, despite having been frequently used to measure modified ribonucleosides (rN) in endogenous rRNA [21] and tRNA [12, 22] LC-MS/MS has been infrequently used to analyze modified ribonucleosides in endogenous mRNA. In addition, in all studies employing LC-MS/MS to measure modified ribonucleosides in endogenous mRNA [23–25] to our best knowledge, merely less than 30 modified ribonucleosides, which represents at most merely 16% of the total number of known naturally occurring modified ribonucleosides in RNA, were included in the measurement. Therefore, developing and utilizing an LC-MS/MS method that enables the analysis of a great number of modified ribonucleosides to analyze mRNA modifications is much needed.

Cancer, a major human disease endangering human health, kills approximately 600,000 people each year [26]. Increasing evidence suggests that RNA modification pathways are misregulated in human cancers and may be ideal targets of cancer therapy [3, 27]. During cancer therapy, however, drug resistance emerges and this has always been a major difficulty in cancer therapy [28–31]. Accumulating data indicates that RNA modifications, such as m^6^A, m^1^A, m^5^C, m^7^G, ψ, and A-to-I editing, are involved in cancer progression and cancer drug resistance [32]. The analysis of RNA modifications in these studies mainly relies on the use of sequencing-based methods [33] that generally suffer from limited coverage of modifications and have limited chemical specificity in modification detection, posing a significant limitation to comprehensively profiling RNA modifications in cancers and deciphering the roles of the RNA modification machinery in cancer pathogenesis and cancer drug resistance.

Here, we report a versatile and sensitive LC-MS/MS-based method for epitranscriptome-wide quantification of mRNA modifications – mRQuant – to enable accurate quantification of 84 types of modified ribonucleosides with sensitivity (amol-fmol) and chemical specificity. Combining this “omic” approach with gene knockdown and transcriptome and proteomic analyses, we provide important new insights into the epitranscriptome landscape in mRNAs in human noncancerous, cancerous, and anticancer drug-resistant cells. Given the significant differences in m^1^A levels observed between cancer and normal cells, and the established role of m^1^A in cancer development [34] we use HeLa cells as our model system to investigate the impact of m^1^A regulatory enzymes on cellular phenotype and mRNA modifications, and revealed some novel functions of m^1^A.

## Materials and methods

### Modified nucleoside standards

Am, m^1^A, m^2^A, m^6^A, m^8^A, m^6^Am, c^7^A, Um, m^3^U, m^5^U, s^2^U, mo^5^U, m^5^Um, hm^5^U, Cm, m^4^C, m^5^C, m^6^C, m^5^Cm, hm ^5^C, f^5^C, s^2^C, ca^5^. C, Gm, m^6^G, m^2^_2_G, c^7^G were purchased from Berry and Associates (Dexter, MI, USA). m^6^_2_Am, ms^2^m^6^A, ms^2^i^6^A, Im, cm^5^U, cmo^5^U, cmnm^5^U, ncm^5^U, m^3^Um, mcm^5^U, mcmo^5^U, m^5^s^2^U, mnm^5^s^2^U, mcm^5^s^2^U, m^5^D, ψ, m^1^ψ, m^3^ψ, s^2^ψ, m^3^C, ac^4^C, ac^4^Cm, f^5^Cm, m^4^_2_Cm, m^2,7^G, m^2, 7^G, imG-14, perQ0 were purchased from Carbosynth (Compton, Berkshire, UK). m^6^_2_A, m^1^acp^3^ψ, ho^5^U, chm^5^U, mcm^5^Um, mchm^5^U, tm^5^U, ncm^5^Um, tm^5^s^2^U, yW, OHyW, OHyWx, O^2^yW, Q, manQ, galQ, Ar(p) ms^2^t^6^A, ms^2^io^6^A were purchased from Chemsoon (Shanghai, China). m^1^Am, i^6^A, t^6^A, m^1^I, D, m^1^G were purchased from Toronto Research Chemicals (Toronto, ON, Canada). I, m^7^G, s^4^U were purchased from Sigma-Aldrich (St. Louis, MO, USA). m^2^G was purchased from MedChemExpress (Monmouth Junction, NJ, USA). ^15^N_5_-dA were purchased from Cambridge Isotope Laboratories (Cambridge, MA, USA). The 84 modified nucleoside standards mentioned above were used for the establishment of mRQuant.

### LC–MS/MS analysis of modified nucleoside standards

Using purchased rNs standards, we optimized the product ion of modified nucleoside standards on the Thermo Fisher Scientific Vanquish™ Flex HPLC system and a Thermo Fisher Scientific TSQ Altis mass spectrometer [35, 36]. The optimization process primarily involved adjusting collision energy (CE) to maximize the sensitivity and specificity of the nucleoside standards. By fine-tuning the CE parameters, we identified optimal conditions for product ion generation to ensure accurate and reliable detection of the nucleoside standards by the mass spectrometer. The optimized mass spectrometry parameters will be used for subsequent DMRM setup. Then we defined the HPLC retention times for the 84 mNs and ^15^N_5_-dA on a Hypersil GOLD aQ C18 column (150 × 2.1 mm, 3 μm) coupled to a Thermo Fisher Scientific Vanquish™ Flex HPLC system and a Thermo Fisher Scientific TSQ Altis mass spectrometer. A solution of 1% Formic Acid in H_2_O (solution A) and 1% Formic Acid in ACN (solution B) were used as mobile phases. The elution was conducted at 36℃ and a flow rate of 400 µL/min, with a gradient of 100% solution A and 0% solution B for 6 min, followed by a linear increase from 0 to 1% solution B over 1.65 min, then from 1 to 6% solution B over 1.7 min, maintaining 6% solution B for 0.65 min, increasing to 50% solution B over 2 min, maintaining 50–75% solution B for 2 min, maintaining 75% solution B for 11 min, followed by a rapid decrease to 0% solution B over 1 s, and finally holding at 100% solution A and 0% solution B for 7 min (column equilibration phase) [12]. The HPLC column was coupled to a Thermo Fisher Scientific TSQ Altis mass spectrometer with an electrospray ionization source in positive mode with the following parameters: Ion Transfer Tube Temp, 350◦C; Sheath Gas, 45Arb; Aus Gas, 15Arb; Vaporizer Temp, 350◦C; Pos Ion Spray Voltage, 3500 V. DMRM mode was used for detection of product ions derived from the precursor ions for all the 84 mNs and ^15^N_5_-dA with instrument parameters which mainly included mode, precursor ion of mN (*m/z*), product ion of mN *m/z*, collision energy (CE), retention time in min, retention time window in min (Table S5).

### Cell culture and treatment

LNCaP Clone FGC human prostate cancer cell line and WPMY-1 human normal prostate stromal cell line were purchased from Pricella Life Science and Technology. SNU-182 human liver cancer cell line and QSG-7701human normal liver cell line were purchased from Hunan Fenghui Biotechnology. These cells were cultured in DMEM supplemented with 10% FBS, 50 ug*/*ml streptomycin and 50 units*/*ml penicillin at 37℃ and 5% CO_2_. BT-20 human breast cancer cell line and Hs 578Bst human normal breast cell line were purchased from YaJi Biological. HeLa was purchased from Pricella Life Science and Technology. HeLa cisplatin-resistant strain (HeLa/DDP) and HeLa paclitaxel-resistant strain (HeLa/PTX) were purchased from Jennio Biotech. These cells were cultured in RPMI-1640 supplemented with 10% FBS, 50 ug*/*ml streptomycin and 50 units*/*ml penicillin at 37℃ and 5% CO_2_. Treatment HeLa with 2.7 µM of DDP or 66 nM of PTX was started when the cell density reached∼70%. Treatment HeLa cisplatin-resistant strain and HeLa paclitaxel-resistant strain with 0.5 µg/mL of DDP or PTX was started when the cell density reached∼70%. Subsequent medication can be increased in concentration based on the cell condition, with incremental concentrations of 0.5 µg/mL, 1 µg/mL, 1.5 µg/mL, and 2 µg/mL. These cells were collected by centrifugation at 250 g for 5 min at 4℃ followed by three washed with ice-cold PBS. All cells were stored at − 80℃ until total RNA extraction.

### Transfection of siRNA

The siRNAs used for HeLa transfection include *TRMT6*, *TRMT61A*, *TRMT10C*, *ALKBH3*, and all siRNAs were synthesized by Sangon Biotech (Table S1). Transfection of siRNA was performed using RNATransMate (Sangon Biotech) according to the manufacturer’s instructions. All transfections were conducted using a siRNA concentration of 50 nM.

### RNA extraction

The total RNA from cells pellets was directly extracted with TRIzol reagent (Invitrogen), according to the manufacturer’s protocol. The poly(A)-tailed RNA was isolated from the total RNA using a Dynabeads mRNA Purification kit (Invitrogen) following the manufacturer’s protocols. The poly(A)-tailed RNA isolated from these cells was subjected to rRNA depletion using Terminator™ 5’-Phosphate-Dependent Exonuclease (Lucigen), following the manufacturer’s protocol. The enzyme was then removed by extraction with chloroform: isoamyl alcohol (24:1, Sevag, Fluka).The poly(A)-tailed RNA in the aqueous layer was then purified by passing through a 3000 Da spin filter (Millipore), followed by washing six times with water. All the poly(A)-tailed RNA samples free from rRNA contamination were stored at − 80℃ before use. The quality of the total RNA (Fig. S1) and poly(A)-tailed RNA (Fig. S2) was assessed using an Agilent Bioanalyzer (Agilent Technologies) with RNA 6000 Pico chips.

### Poly(A)-tailed RNA hydrolysis

Poly(A)-tailed RNA (~ 200 ng) was incubated with1 µL of 50 mM EHNA (Sigma), 2.5 µL of 100 mM Deferoxamine (Sigma), 1 µL of 5 mg/mL Tetrahydrouridine (Sigma), 2.5 µL of 20 mM Butylated hydroxytoluene (Sigma), 1 µL of 5 U/uL Benzonase (Sigma), 5 µL of 0.5 ug/µL Phosphodiesterase I (Sigma), 0.5 µL of 50 U/µL Bacterial Alkaline Phosphatase (Invitrogen), 2.5 µL of 1 M PH 8.0 Tris (Invitrogen), 2.5 µL of 100 mM MgCl_2_ (Sangon Biotech), 10 µL of 200 nM ^15^N_5_-dA and water, making a final volume of ∼50 µL [12]. The enzyme was subsequently removed by extraction with Sevag. All mRNA hydrolysis samples were stored at − 80℃ before use.

### Quantitative real-time PCR

Total RNA was reverse transcribed into cDNA using the PrimeScript™ RT Reagent Kit with gDNA Eraser (TaKaRa), followed by PCR amplification using the primers listed in Table S2 (BGI) and cDNA with TB Green^®^ Premix Ex Taq™ II (TaKaRa) on a fluorescence quantitative PCR instrument (Applied Biosystems).

### Western blot analysis

HeLa cells transfected with siRNA were collected and lysed using 0.3 M Guanidine Hydrochloride (Sigma) and 1% SDS containing 0.1% Protease Inhibitor Cocktail (Sigma) to extract protein samples. The protein samples were incubated with the corresponding primary antibodies: TRMT6 Polyclonal Antibody (Proteintech), TRMT10C Polyclonal Antibody (Proteintech), ALKBH3 Polyclonal Antibody (Proteintech), and TRMT61A Polyclonal Antibody (ORIGENE), followed by incubation with the respective secondary antibodies: Multi-rAb HRP-Goat Anti-Mouse Recombinant Secondary Antibody (Proteintech) and Multi-rAb HRP-Goat Anti-Rabbit Recombinant Secondary Antibody (Proteintech). Signals were detected using Super ECL Prime (Bioscience) and captured with an imaging system. The band intensities of the target proteins were quantified using ImageJ software and normalized to the corresponding internal control proteins GADPH.

### CCK-8 assay

The CCK-8 cell proliferation and cytotoxicity assay was performed using the CCK-8 kit (Saviorbio, G4103). Seven experimental groups were prepared: HeLa cells, *NC-siRNA* cells, *si-ALKBH3*, *si-TRMT10C*, *si-TRMT6*, *si-TRMT61A* and *si-TRMT6-61 A* cells. Cell counting was performed using the cell suspension mixed with trypan blue solution on a Count Star cell counting plate and analyzed with a Count Star cell counter.

### Cell cycle and apoptosis analysis

Cell cycle analysis was performed using a Cell Cycle and Apoptosis Detection Kit (Beyotime, C1052). Collect the transfected cells and fix them with ice-cold 70% ethanol for 12 h. After fixation, stain the cells with propidium iodide (PI) and analyze them using flow cytometry with an excitation wavelength of 488 nm.

Cell apoptosis was evaluated using the Annexin V-FITC Apoptosis Detection Kit (Beyotime, C1062). Collect the transfected cells, resuspend them in Annexin V-FITC binding buffer, and incubate with PI solution at room temperature in the dark for 10–20 min. Apoptotic cells were then detected using flow cytometry.

### Transcriptomic and proteomic analyses

NC-siRNA, *TRMT6*-siRNA, *TRMT61A*-siRNA, *TRMT6/61A*-siRNA, *TRMT10C*-siRNA, and *ALKBH3*-siRNA were transfected into HeLa cells as described previously. The mRNA and protein expression levels of m^1^A regulatory enzyme genes before and after transfection were assessed by RT-qPCR and Western blotting, respectively. Cells from the Control group, siTRMT6 group, siTRMT61A group, siTRMT6/61A group, siTRMT10C group, and siALKBH3 group were collected and sent to OE Biotech Co., Ltd. (shanghai, China) for subsequent transcriptomic and proteomic sequencing and analysis.

## Results

### Development of a sensitive LC-MS/MS Method-mRQuant for quantifying mRNA modifications

**Fig. 1:**
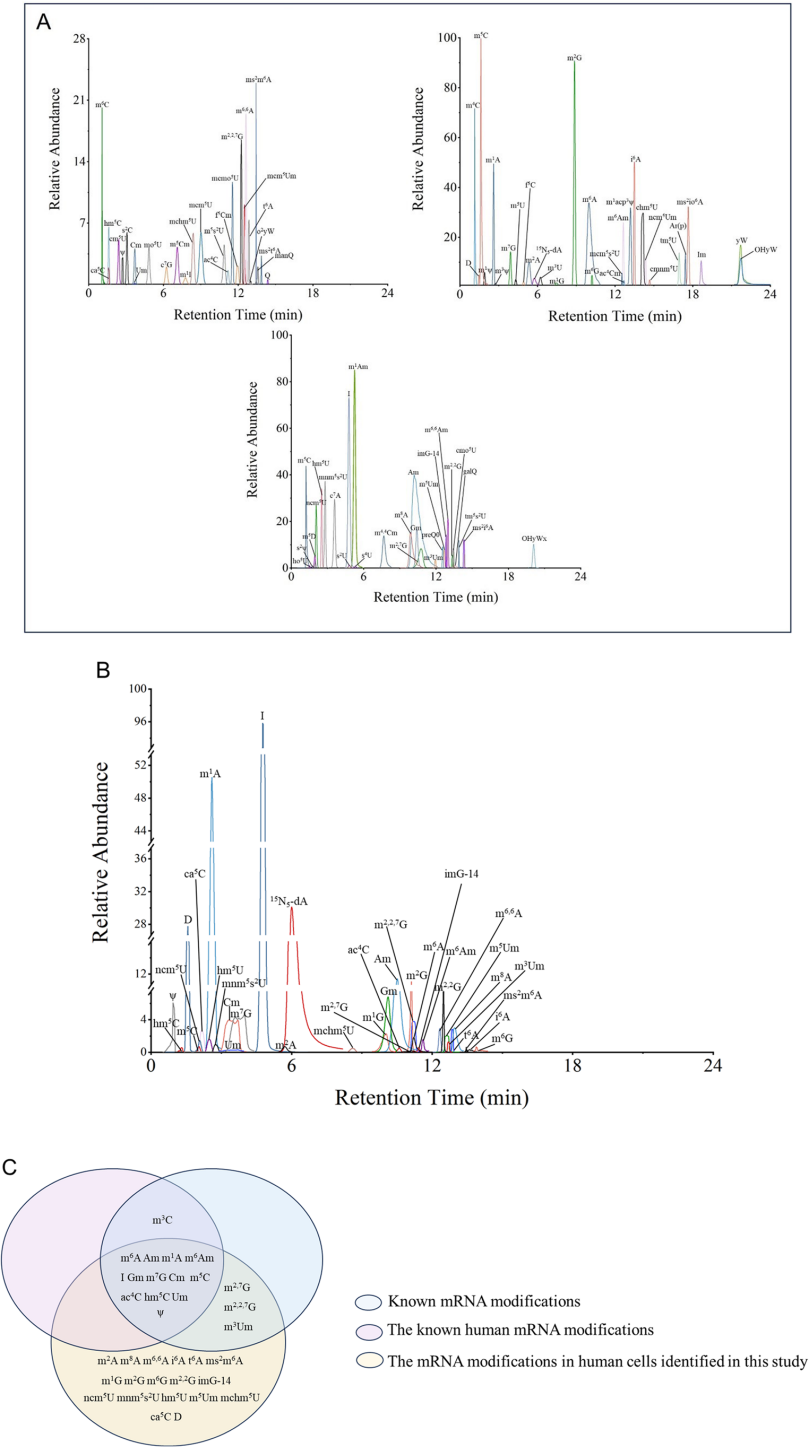
Modified nucleosides detected using mRQuant technology. (A) Retention times of 84 modified nucleosides standards and internal standard ^15^N_5_-dA. (B) Retention time of 34 modified nucleosides and internal standard ^15^N_5_-dA detected in mRNA of liver normal epithelial cells QSG-7701. (C) Comparison of known mRNA modifications, known human mRNA modifications, and the human mRNA modifications identified in this study

As shown in Fig. 1A, we developed an LC-MS/MS method to quantify 84 out of the approximately 170 known ribonucleoside modifications in cellular mRNA. First, we isolated total RNA from cultured cells and purified mRNA from them using OligoT beads to obtain mRNA. Then, we removed contaminating rRNAs from the mRNA using a 5’-phosphate-dependent exonuclease. Next, we digested the purified mRNA into individual rNs using a mixture of nucleases, phosphatase, antioxidant, deaminase inhibitors, and an internal standard, ^15^N_5_-dA. The resulting rNs were separated by reversed-phase HPLC and identified by mass spectrometry using CID fragmentation patterns and by comparison to corresponding chemical standards. Quantification was performed using pre-determined molecular transitions in dMRM mode during CID in the LC-MS/MS system (Fig. S3). Using this method, we detected and quantified 26–34 modifications in mRNA from several human cell lines, both with and without drug treatment.

Our approach emphasizes quantitative accuracy to precisely measure even small changes in the relative abundance of rNs. We used OligoT beads to purify mRNA and further removed its contaminating rRNA content to furnish high-purity mRNA. An internal standard, ^15^N_5_-dA, was used to reduce variation in measuring individual rNs. Our method also provides high coverage, identifying and quantifying 84 modified rNs using chemically synthesized standards (Fig. 1A). The method also provides excellent sensitivity, with limits of detection (LOD) for the 84 modified nucleosides ranging from 0.01 amol to 100 fmol (Table S4).

However, two groups of isobaric modified rNs (m^3^C, m^4^C, m^5^C and m^6^C; m^6^A and m^8^A) were challenging to differentiate due to their close retention times and similar CID fragmentation patterns, which made their identification and quantification difficult. Nevertheless, by utilizing single standard spiking (m^3^C, m^4^C, m^5^C and m^6^C; Fig. S16) or differences in fragmentation (m^6^A and m^8^A; Fig. S17), we were able to identify them unambiguously and quantify them in the cell samples. We obtained highly reproducible data for the 34 detected modified rNs (Fig. 1B).

Subsequently, we compared the 34 modified rNs detected in this study with the currently known mRNA modifications and human-specific mRNA modifications. The overlapping areas revealed the types of modifications that they have in common. Among them, 18 modified rNs detected in this study have not been previously reported in mRNAs (Fig. 1C). These unreported modifications may stem from contamination with tRNA or could represent putative new mRNA modifications. Further research is needed to confirm the origin and nature of these modifications.

These results demonstrate that mRQuant is sensitive, precise, and accurate in the analysis of the mRNA epitranscriptome.

### Exploring the mRNA modification profiles in Cancer and normal cells

Changes in modified nucleosides may serve as biomarkers for cancer diagnosis, prognosis, or therapeutic response evaluation. The differences in modified nucleosides between normal and cancer cells are instrumental in elucidating the mechanisms underlying cancer development, progression, and regulation.

**Fig. 2:**
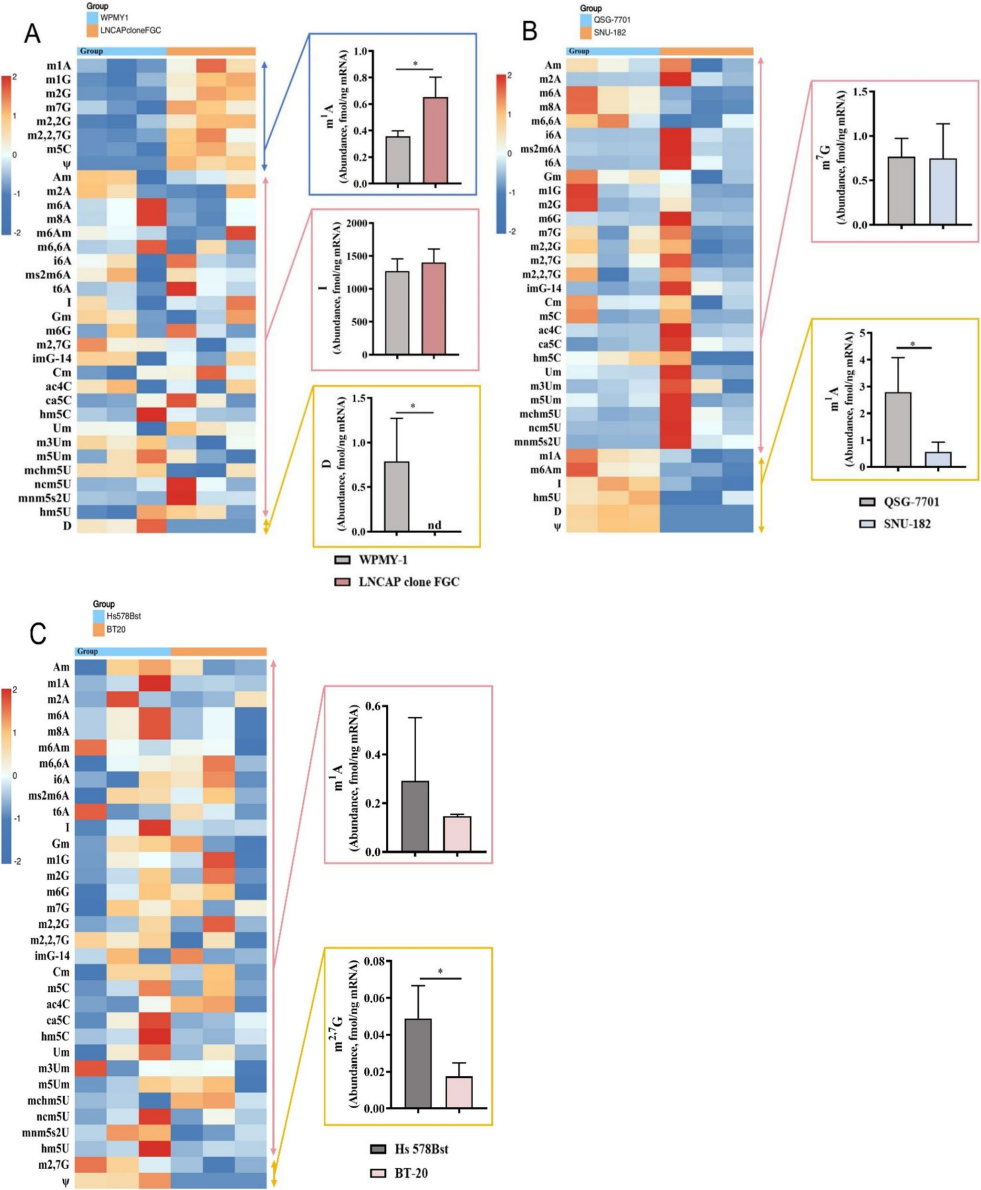

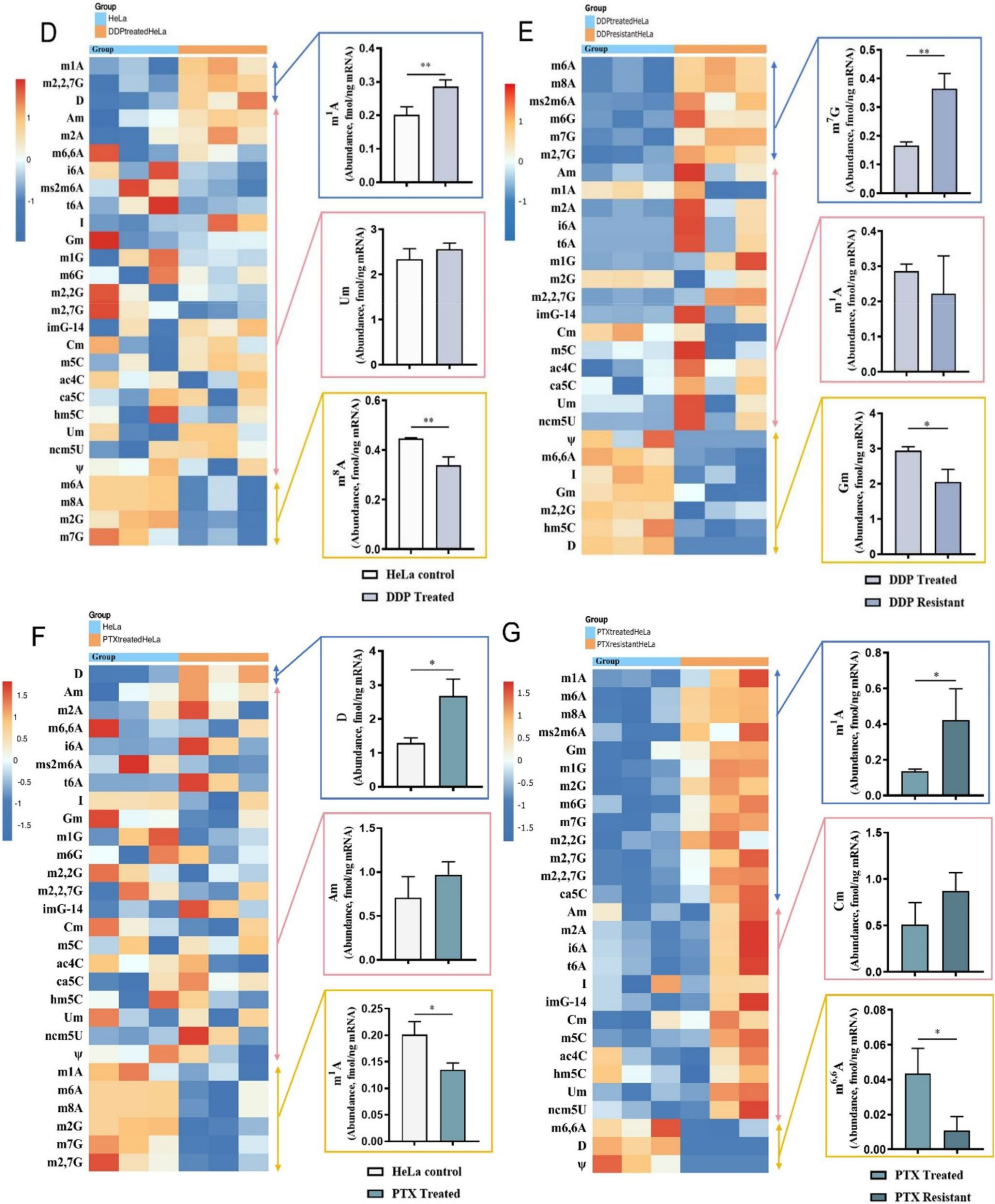
The relative abundance changes of 32–34 modified nucleosides in cellular mRNA were quantified by LC-MS/MS and visualized in a heatmap. On the right side of the heatmap, a representative bar chart was selected for each group of modifications based on their change trends. (A) WPMY-1 and LNCAP clone FGC. (B) QSG-7701 and SNU-182. (C) Hs 578Bst and BT-20. (D) HeLa cells and HeLa cells treated with DDP. (E) HeLa cells treated with DDP and DDP-resistant HeLa cells. (F) HeLa cells and HeLa cells treated with PTX. (G) HeLa cells treated with PTX and PTX-resistant HeLa cells

This approach was applied to analyze mRNA from human prostate cancer cells LNCaP Clone FGC and normal cells WPMY-1, liver cancer cells SNU-182 and normal cells QSG-7701, and breast cancer cells BT-20 and normal cells Hs 578Bst. Among the 84 types of targeted modified nucleosides (Fig. 1A), 32–34 were detected (Fig. 2A, B and C and Fig. S4-S6).

Compared to the modified nucleosides in the mRNA of human normal prostate stromal immortalized cell line WPMY-1, eight modified nucleosides in the mRNA of human prostate cancer cell line LNCaP Clone FGC showed significantly increased abundance (Fig. 2A and Fig. S4), namely m^1^A (*P* < 0.05), m^1^G (*P* < 0.01), m^2^G (*P* < 0.01), m^7^G (*P* < 0.05), m^2^G (*P* < 0.01), m^2, 7^G (*P* < 0.05), m^5^C (*P* < 0.01), and ψ (*P* < 0.01), with only D showing a significant decrease (*P* < 0.05). Specifically, D was found in the mRNA of the human normal prostate stromal immortalized cell line WPMY-1, whereas ψ was not. In the mRNA of human prostate cancer cell line LNCaP Clone FGC, ψ was detected but not D.

Interestingly, no significantly increased modified nucleosides were found in the mRNA of human liver cancer cells SNU-182 compared to those in the mRNA of human normal liver cells QSG-7701, while six showed significantly decreased abundance (Fig. 2B and Fig. S5), namely m^1^A (*P* < 0.05), m^6^Am (*P* < 0.05), I (*P* < 0.01), hm^5^U (*P* < 0.05), ψ (*P* < 0.001), and D (*P* < 0.001). Notably, ψ and D were absent in human liver cancer cells SNU-182.

Remarkably, no significantly increased modified nucleosides were found in the mRNA of human breast cancer cells BT-20 compared to those in human normal breast cells Hs 578Bst, while two showed significantly decreased abundance (Fig. 2C and Fig. S6), namely m^2, 7^G (*P* < 0.05) and ψ (*P* < 0.05). Interestingly, ψ was not detected in human breast cancer cells BT-20.

The results showed that in the mRNA of the six cancer and non-cancer cell lines mentioned above, the most abundant modification was I, while the least abundant modifications (Fig. S4-S6) were m^6^Am (LNCaP Clone FGC), m^2^G (WPMY-1), and m^2^A (SNU-182, QSG-7701, BT-20, and Hs 578Bst).

### Investigating drug-Induced and drug Resistance-Associated mRNA modifications in HeLa cells

Cisplatin (DDP, Cisplatin) and paclitaxel (PTX, Paclitaxel) are two classic anticancer drugs that inhibit the proliferation of cancer cells through different mechanisms. Cisplatin binds to DNA, interfering with its replication and repair processes, thereby inducing apoptosis. In contrast, paclitaxel inhibits the depolymerization of microtubules, thereby preventing mitosis. Based on this, we focused on mRNA modifications in HeLa cells under various conditions: untreated, treated with the drugs DDP and PTX, and resistant to DDP and PTX.

By examining the changes in modified nucleosides among these different states, we aim to pinpoint the alterations induced by drug exposure and to decipher how resistance to these drugs is associated with changes in nucleoside modifications. This approach is vital for unraveling the mechanisms of drug resistance in cancer cells. Comparing the amounts of detected modified nucleosides in the mRNA of HeLa cells, HeLa cells treated with DDP and PTX, and HeLa cells resistant to DDP and PTX revealed several significant specific differences. Among the 84 types of targeted modified nucleosides, 26–28 were detected (Fig. 2D, E, F and G and Fig. S7-S8).

Compared to the modified nucleosides in the mRNA of HeLa cells, three modified nucleosides in the mRNA of HeLa cells treated with DDP showed significantly increased abundance (Fig. 2D and Fig. S7), namely m^1^A (*P* < 0.01), m^2, 7^G (*P* < 0.01), and D (*P* < 0.05), and four showed significantly decreased abundance, namely m^6^A (*P* < 0.01), m^8^A (*P* < 0.01), m^2^G (*P* < 0.01), and m^7^G (*P* < 0.05). Compared to the modified nucleosides in the mRNA of HeLa cells treated with DDP, six modified nucleosides in the mRNA of HeLa cells resistant to DDP showed significantly increased abundance(Fig. 2E and Fig. S7), namely m^6^A (*P* < 0.001), m^8^A (*P* < 0.001), ms^2^m^6^A (*P* < 0.01), m ^6^G (*P* < 0.05), m^7^G (*P* < 0.01), and m^2, 7^G (*P* < 0.01), and six showed significantly decreased abundance, namely m^6^A (*P* < 0.01), I (*P* < 0.01), Gm (*P* < 0.05), m^2^G (*P* < 0.05), hm^5^C (*P* < 0.01), and D (*P* < 0.001). It is worth noting that both ψ and D were detected in the mRNA of DDP-treated HeLa cells, whereas neither was detected in the mRNA of DDP-resistant HeLa cells.

Compared to the modified nucleosides in the mRNA of HeLa cells (Fig. 2F and Fig. S8), only D showed significantly increased abundance in the mRNA of HeLa cells treated with PTX (*P* < 0.05), while six showed significantly decreased abundance, namely m^1^A (*P* < 0.05), m^6^A (*P* < 0.05), m^8^A (*P* < 0.05), m^2^G (*P* < 0.01), m^7^G (*P* < 0.05), and m^2, 7^G (*P* < 0.05). Compared to the modified nucleosides in the mRNA of HeLa cells treated with PTX, thirteen modified nucleosides in the mRNA of HeLa cells resistant to PTX showed significantly increased abundance (Fig. 2G and Fig. S8), namely m^1^A (*P* < 0.05), m^6^A (*P* < 0.01), m^8^A (*P* < 0.01), ms^2^m^6^A (*P* < 0.05), Gm (*P* < 0.05), m^1^G (*P* < 0.05), m^2^G (*P* < 0.01), m^6^G (*P* < 0.05), m^7^G (*P* < 0.05), m^2^G (*P* < 0.05), m^2, 7^G (*P* < 0.05), m^2, 7^G (*P* < 0.05), and ca^5^C (*P* < 0.05), and three showed significantly decreased abundance, namely m^6^A (*P* < 0.05), D (*P* < 0.001), and ψ (*P* < 0.05). It is noteworthy that ψ and D were present in the mRNA of PTX-treated HeLa cells, whereas neither was found in the mRNA of PTX-resistant HeLa cells.

The results showed that in the mRNA of HeLa cells, HeLa cells treated with DDP and PTX, and HeLa cells resistant to DDP and PTX, the highest abundance of modified nucleosides was I. The lowest abundance modified nucleoside was m^6^A in the DDP-resistant HeLa cells, while ms^2^m^6^A was the lowest in HeLa cells, DDP-treated HeLa cells, PTX-treated HeLa cells, and PTX-resistant cells (Fig. S7 and S8).

### The impact of knocking down the m^1^A regulatory enzyme on HeLa

m^1^A is an internal base modification in mRNA that, although previously underrecognized, is highly prevalent. It plays a crucial role in regulating mRNA translation, reprogramming cancer cell metabolism, and helping cancer cells adapt to harsh environments. To date, research on the regulatory enzymes associated with m^1^A modification on mRNA has made some progress. In this study, we found significant differences in the levels of m^1^A modification (Fig. 2 and Fig. S4-S8) in the mRNA of cancer cells compared with normal cells, HeLa cells, their drug-treated groups, and drug-resistant strains. Based on these findings, we plan to explore the effects of knocking down the expression of m^1^A modification-related regulatory enzymes in HeLa cells, in order to study their roles in cellular functions and characteristics. This approach will help us gain a deeper understanding of the mechanisms underlying m^1^A modification in cell biology and its potential contribution to the development of drug resistance.

**Fig. 3:**
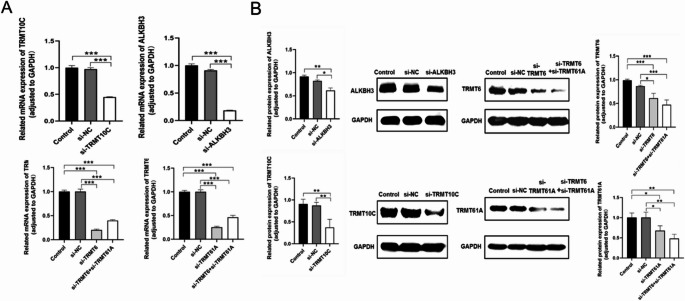
siRNA decreases the mRNA and protein levels of m^1^A regulatory enzymes in HeLa cells. (A) Relative expression changes of mRNA for *TRMT10C*, *ALKBH3*, *TRMT6*, *TRMT61A*, and *TRMT6-61 A* were measured by RT-qPCR. The x-axis represents the treatment groups, while the y-axis indicates the mRNA expression changes of m^1^A regulatory enzymes relative to the reference gene (GAPDH). (B) Protein level changes of TRMT10C, ALKBH3, TRMT6, TRMT61A, and TRMT6-61 A were assessed by Western blotting. Protein bands were quantified using ImageJ software. The x-axis represents the treatment groups, and the y-axis shows the changes in m1A regulatory enzymes relative to the reference gene (GAPDH). Each group was conducted with three biological replicates. * *p* < 0.05, ** *p* < 0.01, *** *p* < 0.001

HeLa cells were chosen as a model for our study, and we targeted the human m^1^A methyltransferase genes *hTRMT6*, *hTRMT61A*, *hTRMT6-61 A*, *hTRMT10C*, and the demethylase gene *hALKBH3* for knockdown. The expression of these five m^1^A regulatory enzyme genes was confirmed to be significantly reduced at both the mRNA (Fig. 3A) and protein levels (Fig. 3B) through RT-qPCR and Western blot analyses.

Subsequently, we utilized the mRQuant platform to explore the impact of these knockdowns on m^1^A levels in mRNA (Fig. S9b). We observed a significant decrease in m^1^A levels following the knockdown of the methyltransferase genes *hTRMT6*, *hTRMT61A*, *hTRMT6-61 A*, and *hTRMT10C*. In contrast, the knockdown of the demethylase gene hALKBH3 resulted in a significant increase in m^1^A abundance. Additionally, changes in m^1^A abundance also influenced the levels of other modifications (Fig. S9).

**Fig. 4:**
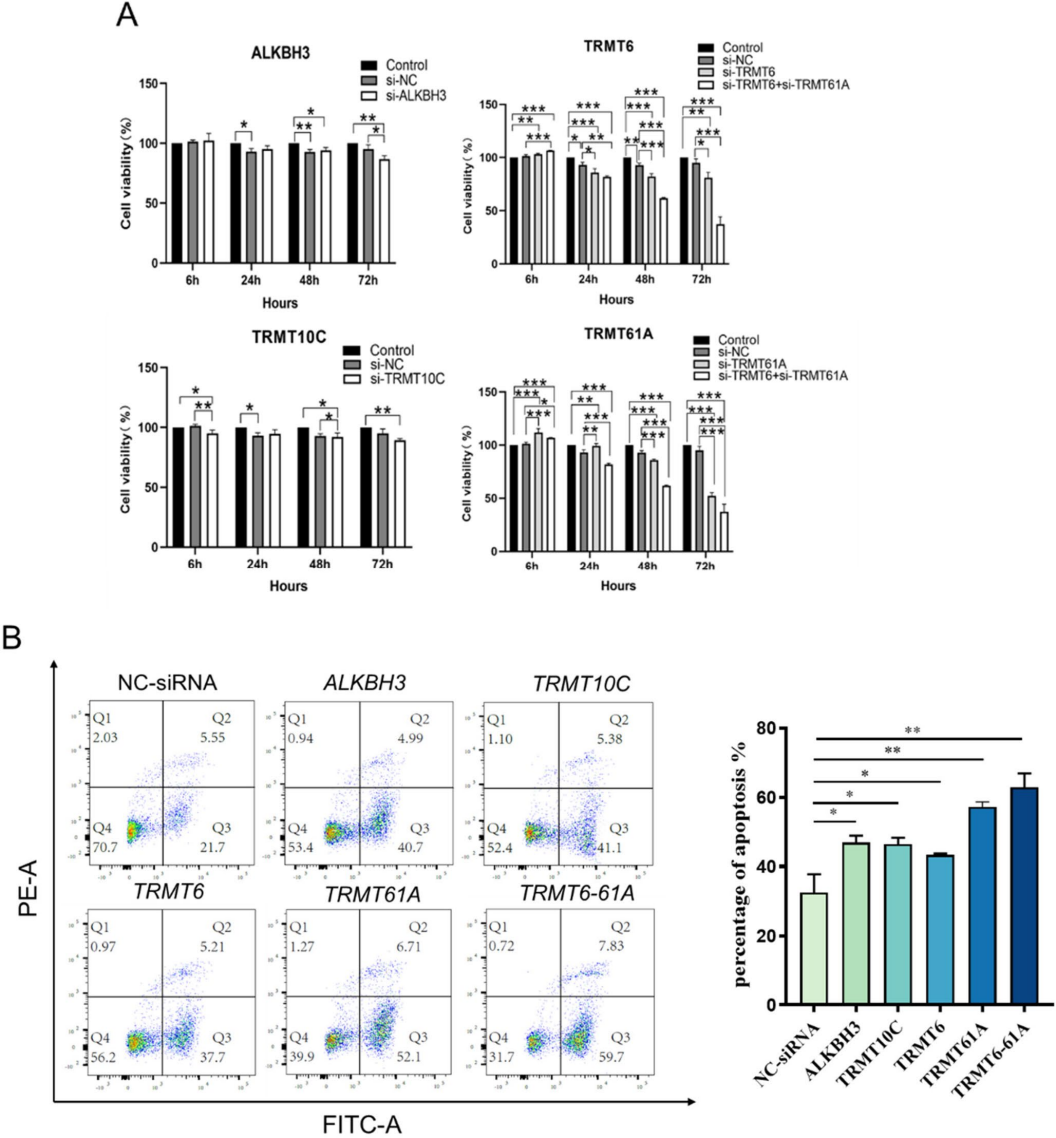
Effects of m^1^A regulatory enzyme knockdown on cell viability. (A) CCK8 assay evaluating changes in cell proliferation following knockdown of m^1^A regulatory enzymes *TRMT10C*, *ALKBH3*, *TRMT6*, *TRMT61A*, and *TRMT6-61 A* at 6 h, 24 h, 48 h, and 72 h. The x-axis represents treatment duration, and the y-axis indicates cell viability. Each group consists of three biological replicates. * *p* < 0.05, ** *p* < 0.01, *** *p* < 0.001. (B) Flow cytometry measurement of apoptosis rates after 72 h of knockdown of m^1^A regulatory enzymes *TRMT10C*, *ALKBH3*, *TRMT6*, *TRMT61A*, and *TRMT6-61 A*. Cell apoptosis percentages were quantified using GraphPad Prism software; the x-axis represents m^1^A regulatory enzymes, and the y-axis represents the percentage of apoptosis. Each group consists of three biological replicates. * *p* < 0.05, ** *p* < 0.01, *** *p* < 0.001

We also observed significant effects of the knockdown of these m^1^A regulatory enzyme genes on cell viability (Fig. 4A) and cell cycle progression (Fig. S10; Table S3). Flow cytometry analysis further revealed that, compared to the control group, the apoptosis rates of HeLa cells significantly increased after the knockdown of each gene (Fig. 4B): *hALKBH3* (47.0 **±** 1.9%), *hTRMT10C* (46.5 **±** 1.9%), *hTRMT6* (43.3 **±** 0.43%), *hTRMT61A* (57.2 **±** 1.4%), and *hTRMT6-61 A* (62.9 **±** 4.0%).

In summary, upon downregulating key m^1^A regulatory enzymes, we observed significant changes in the cell cycle, apoptosis, and survival rates, indicating that m^1^A modification plays an important role in these biological processes.

### Comprehensive transcriptomic and proteomic characterization after knockdown of mRNA m^1^A writer or eraser proteins

To explore the roles of mRNA m^1^A modification enzymes in HeLa cells, we used siRNA technology to suppress the expression of *ALKBH3*, *TRMT6-61 A*, *TRMT6*, *TRMT61A* and *TRMT10C* genes. We then conducted transcriptomic and proteomic analyses on key functional gene sets affected by these genes.

**Fig. 5:**
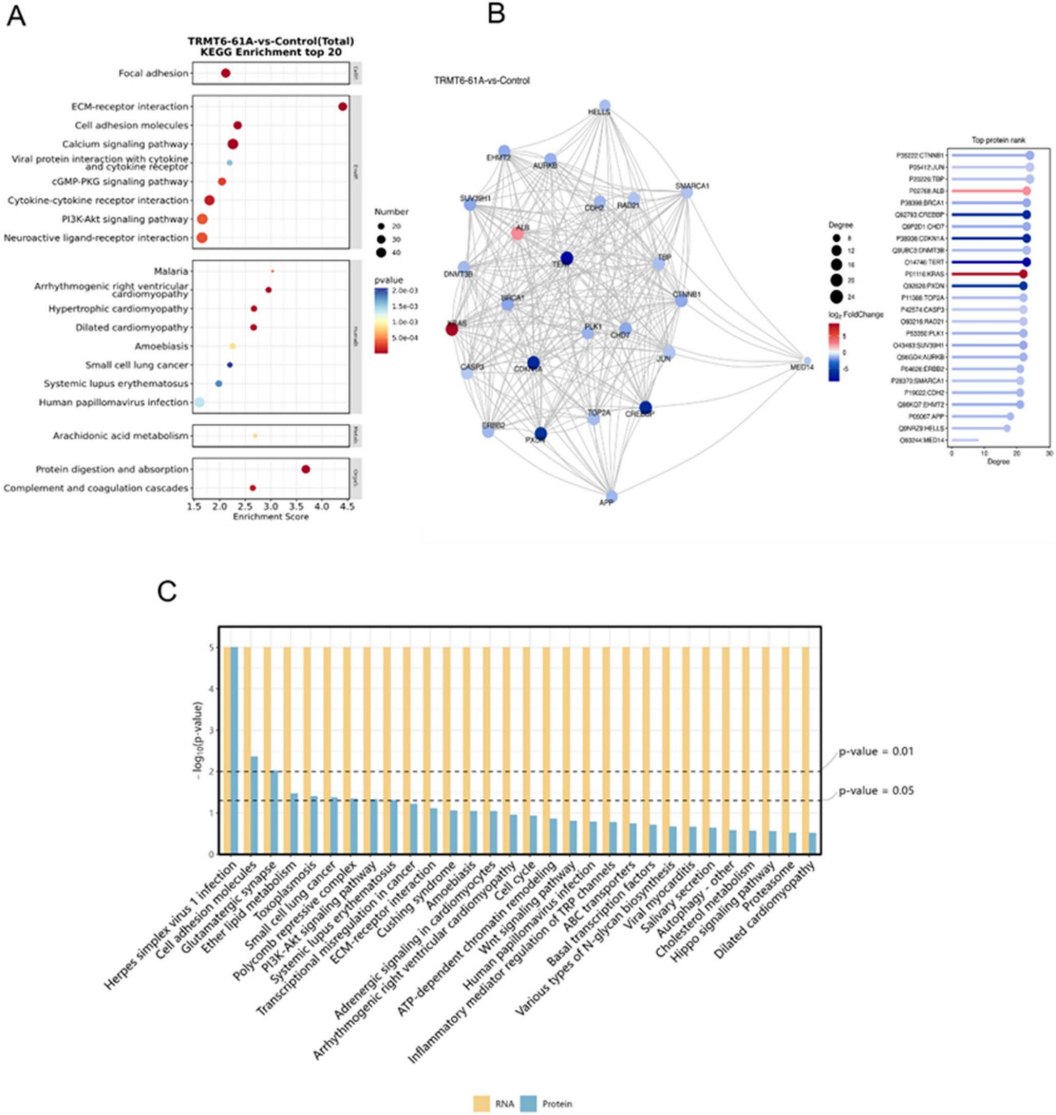
Transcriptomic and proteomic analysis of *TRMT6-61 A* treated HeLa cells: Transcriptome: (A) Top 20 KEGG enrichment bubble chart. Proteome: (B) Top 25 connectivity protein interaction network diagram. Combined transcriptomic and proteomic analysis: (C) Bar chart of the top 30 KEGG (GSEA) pathways shared across different omics

Upon analyzing differentially expressed genes (DEGs), we found that the upregulated DEGs in the knockdown groups of *ALKBH3*, *TRMT6-61 A*, *TRMT6*, *TRMT61A* and *TRMT10C* were 258, 847, 209, 714, and 298, respectively, while the downregulated DEGs were 181, 1295, 243, 1060, and 215, all meeting the criteria of fold change (FC > 2 and *P* < 0.05). Gene Ontology (GO) and Kyoto Encyclopedia of Genes and Genomes (KEGG) enrichment analyses showed that *ALKBH3* and *TRMT10C* are involved in the negative regulation of protein phosphorylation and the Wnt signaling pathway (Fig. S11b and S15b). *TRMT6-61 A* and *TRMT61A* are related to extracellular matrix organization and cell adhesion pathways (Fig. S12b and S14b), while *TRMT6* is associated with the positive regulation of neuroinflammatory responses and podocyte differentiation (Fig. S13b). All five m^1^A modification enzymes were significantly enriched in the calcium signaling pathway (Fig. 5A, Fig. S11a and Fig. S13a -S15a). Further analysis of differential transcription factors and their target genes revealed that the differential transcription factors for these five m^1^A modification enzymes were mainly enriched in the ETS family (Fig. S11c, Fig. S12a and Fig. S13c −15c).

In the analysis of differentially expressed proteins (DEPs), we found that the knockdown groups of *ALKBH3*, *TRMT6-61 A*, *TRMT6*, *TRMT61A* and *TRMT10C* had 278, 1074, 297, 914, and 304 significantly differentially expressed proteins, respectively, meeting the criteria of fold change (FC > 2 and *P* < 0.05). KEGG enrichment analysis revealed that ALKBH3 and TRMT10C are involved in the regulation of lifespan regulation pathways and FoxO signaling pathways (Fig. S11f and Fig. S15f). TRMT6-61 A and TRMT61A are related to ATP-dependent chromatin remodeling and lysine degradation pathways (Fig. S12c and S14f), while the TRMT6 knockdown group is involved in tyrosine metabolism and longevity regulation pathways (Fig. S13f). Further GO enrichment analysis showed that ALKBH3 is significantly enriched in processes such as NAD(P)H oxidase H_2_O_2_ formation activity and glutathione peroxidase activity (Fig. S11e). TRMT10C is enriched in processes like actin filament regulation and nitrobenzene metabolism (Fig. S15e). For the *TRMT6-61 A*, *TRMT6* and *TRMT61A* treatment groups, enrichment was observed in transcription co-regulator activity (Fig. S12d, S13e and S14e).

Further analysis of the protein interaction networks (Top 25 high-connectivity proteins) revealed that in the *TRMT6-61 A* and *TRMT61A* knockdown groups, the expression of the CTNNB1 protein was significantly downregulated (Fig. 5B and Fig. S14d). In contrast, in the *ALKBH3*, *TRMT6*, and *TRMT10C* knockdown groups, the expression of the TGEBR2 protein was generally upregulated (Fig. S11d, S13d, and S15d). Additionally, in the *TRMT61A* and *TRMT10C* knockdown groups, the expression of KRAS protein also showed an upregulation trend (Fig. S14d and S15d).

To further decipher the biological processes regulated by m^1^A-related modification enzymes in HeLa cells, we integrated the transcriptomic and proteomic data. The results showed that in the *ALKBH3* knockdown group, protein expression levels in oxidative phosphorylation processes were higher than RNA levels, while the opposite phenomenon was observed in the *TRMT10C* knockdown group. Both groups exhibited a consistent trend of protein levels being lower than RNA levels in N-glycan biosynthesis and central carbon metabolism in cancer pathways (Fig. S11g and S15g). In the *TRMT6-61 A*, *TRMT6*, and *TRMT61A* knockdown groups, protein expression levels in the PI3K-Akt signaling pathway and ATP-dependent chromatin remodeling process were generally lower than RNA levels (Fig. 5C, Fig. S13g and S14g).

## Discussion

In this study, we developed the mRQuant platform, which demonstrated outstanding performance in high-throughput and highly sensitive quantitative analysis of 84 modified ribonucleosides in mRNA, achieving sensitivity at attomole (amol) levels. This significantly expands the boundaries of RNA modification research. The mRQuant platform performs exceptionally well under various experimental conditions, accurately capturing subtle changes in RNA modifications not only in normal and cancer cells but also in different drug treatment scenarios.

Compared to existing methodologies, the mRQuant platform has the following technical improvements. First, it substantially increases the coverage of modified ribonucleosides, from less than 30 to 84, in the analysis of mRNA modifications. Second, it achieves detection and quantification of most of the 84 modified ribonucleosides from mRNA with sensitivity at attomole levels. Third, it provides a broad dynamic range (4–5 orders of magnitude) in the detection of 84 modified ribonucleosides in mRNA. Fourth, it provides high-throughput analysis of mRNA modifications, with acquisition of data from the detection of 84 modified ribonucleosides in a 25-min run. Finally, it enables unambiguous identification and quantification of challenging-to-differentiate naturally-occurring isobaric modified ribonucleosides from mRNA, e.g. m^3^C, m^4^C, m^5^C and m^6^C; m^6^A and m^8^A.

However, we also recognize that despite implementing stringent quality control measures to ensure the purity of mRNA samples during RNA extraction, slight contamination from rRNA and tRNA might still occur. Such potential contamination could lead to signal overlap, affecting the accurate interpretation of low-abundance modifications. Therefore, we recommend further optimization of sample purification and analysis workflows in future studies to more precisely distinguish low-abundance modifications from potential contaminant signals.

RNA modifications play a crucial role in tumorigenesis and cancer progression by regulating processes such as cell proliferation, differentiation, invasion, migration, stemness, metabolism, and drug resistance, thereby either promoting or suppressing tumor development. Using our newly developed mRQuant platform, we detected multiple mRNA modifications that differ significantly in abundance between noncancerous and cancer cells. These modifications include well-characterized internal mRNA modifications that have been associated with various cancers, such as 5-methylcytosine (m^5^C), 7-methylguanosine (m^7^G), pseudouridine (ψ), inosine (I), and N¹-methyladenosine (m^1^A), which were found to be mRNA modifications that differ between noncancerous and cancer cells in previous studies as well [10].

The findings from our study align with previous research on the role of specific mRNA modifications in cancer. For instance, m^5^C modification is often highly expressed in tumor tissues and circulating cancer cells [37], where it enhances the stability and expression of oncogene mRNAs, promotes metabolic reprogramming, and ultimately drives tumor cell proliferation, invasion, and migration [38–46]. The m^7^G modification and its regulatory enzymes influence tumor progression by modulating translation and miRNA biogenesis. Notably, the m^7^G cap is closely associated with tumorigenesis, as cap methylation in oncogene mRNAs facilitates their nuclear export and translation [47–49]. Additionally, ψ modification affects tumorigenesis by regulating translation or RNA stability [10], while A-to-I editing influences protein synthesis by modulating mRNA stability in tumors and can also stabilize tumor suppressor gene expression, thereby inhibiting tumor progression [50–53]. The m^1^A modification and its regulatory enzymes play significant roles in tumor cell proliferation, apoptosis, invasion, migration, cancer stem cell (CSC) self-renewal, and metabolism by modulating the stability or translation of oncogenes or tumor suppressor genes [54–59].

Beyond the well-characterized modifications, the mRQuant platform also identified several new mRNA modifications that significantly differ in abundance between cancer and noncancerous cells. These include m^1^G, m^2^G, m^2^G, and m^2, 7^G in LNCaP Clone FGC and WPMY-1 (Fig. S4); m^6^Am and hm^5^U in SNU-182 and QSG-7701 (Fig. S5); m^2, 7^G in BT-20 and Hs 578Bst (Fig. S6); and D in SNU-182, QSG-7701, BT-20, and Hs 578Bst (Fig. S5 and Fig. S6). The biological functions and mechanisms of these novel modifications remain to be explored. Future studies are needed to elucidate their roles in tumorigenesis and cancer progression.

In this study, compared to untreated HeLa cells, the mRNA of HeLa cells treated with DDP showed significantly increased abundance of m^1^A, m^2, 7^G, and D, while m^6^A, m^8^A, m^2^G and m^7^G were significantly decreased. These findings are consistent with previous studies demonstrating that cisplatin can significantly alter RNA modification patterns in cancer cells. For instance, Xie [60] reported that cisplatin treatment in bladder cancer led to increased levels of ac^4^C modifications in tumor cells, enhancing the stability of DNA repair-related genes and contributing to chemotherapy resistance. Similarly, Yu [61] found that in KRAS-mutant non-small cell lung cancer cells, cisplatin treatment increased overall m^6^A modification levels by regulating the post-translational modifications (PTMs) of ALKBH5, thereby enhancing DNA damage repair capacity and promoting resistance to platinum-based drugs. These studies corroborate our findings, further validating the impact of cisplatin on RNA modification profiles.

In HeLa cells resistant to DDP, compared to those treated with DDP, we observed significantly increased abundance of m^6^A, m^8^A, ms^2^m^6^A, m^6^G, m^7^G, and m^2, 7^G, while m^6^A, I, Gm, m^2^G, hm^5^C, and D were significantly decreased. Notably, ψ and D were detected in the mRNA of DDP-treated HeLa cells but were undetectable in DDP-resistant cells. This suggests that these specific nucleoside modifications may be associated with cellular resistance to DDP. Wei [62] found that m^6^A levels are higher in cisplatin-resistant cells than in normal cells, with m^6^A-related regulatory proteins enhancing the m^6^A methylation levels of specific genes, thereby affecting mRNA stability. These results indicate that RNA modification profiles may play a crucial role in mediating clinical responses during DDP treatment.

Our findings on mRNA modifications in PTX-Treated HeLa cells align with previous research showing that PTX can modulate cancer cell drug resistance by altering mRNA m^6^A modifications. Yuan [63] demonstrated that RBM15 regulates m^6^A modifications by binding to target RNAs and recruiting the methyltransferase complex, thereby influencing ovarian cancer cell resistance to PTX. This suggests that PTX may alter RNA modification patterns to impact on drug resistance in cancer cells.

Based on the differential modifications identified in this study, these RNA modifications may influence biological functions such as DNA repair and gene expression stability under cisplatin and paclitaxel treatment by affecting RNA modification levels or the activity of related modifying enzymes. Consequently, these modifications may play a role in determining cell sensitivity or resistance to these drugs. The discovery of these modifications not only deepens our understanding of the mechanisms underlying cisplatin and paclitaxel action but also provides potential targets for developing new therapeutic strategies.

Additionally, after knocking down m^1^A-modifying enzymes *TRMT6*, *TRMT61A*, *TRMT6-61 A*, and *TRMT10C*, m^1^A levels decreased significantly, while knocking down the demethylase *ALKBH3* led to a significant increase in m^1^A levels. Other modifications, such as m^1^G and m^3^C, exhibited similar trends. *ALKBH3* is known to be a demethylase for m^1^A and m^3^C [55], while *TRMT10C* is involved not only in m^1^A methylation but also in m^1^G methylation [64]. These findings suggest that RNA-modifying enzymes might have cross-reactivity for other modifications or influence other modifications indirectly, influencing the overall regulation of RNA metabolism and modification networks.

By integrating proteomic and transcriptomic data, we found that m¹A regulatory enzymes are involved in multiple cancer-related signaling pathways, such as the Wnt signaling pathway and the PI3K-Akt signaling pathway, both of which play critical roles in tumor initiation, progression, and drug resistance [63, 65]. For example, TRMT61A-mediated tRNA-m¹A modification regulates the synthesis of MYC protein, thereby influencing tumor cell proliferation. Additionally, it can modulate PD-L1 synthesis, affecting the immune evasion capacity of tumor cells. This suggests that TRMT61A inhibitors may serve as a novel strategy to enhance tumor immunotherapy and oncolytic virus therapy sensitivity [66]. Moreover, these modification enzymes are also associated with processes such as extracellular matrix organization, cell adhesion, and oxidative phosphorylation, all of which are crucial in cancer invasion, metastasis, and drug resistance [32]. Therefore, m^1^A regulatory enzymes constitute excellent therapeutic targets for cancer treatment and cancer drug resistance.

## Supplementary information

Below is the link to the electronic supplementary material.

ESM 1 (DOCX 14.2 MB)

## Data Availability

All data are available in the main text or supplementary materials and the raw mass spectrometry data have been deposited to the ProteomeXchange Consortium via PRIDE.

## Declarations

### Authors' information

State Key Laboratory of Reproductive Regulation and Breeding of Grassland Livestock, Institute of Biomedical Sciences, School of Life Sciences, Inner Mongolia University, 010020, Hohhot, China.

Ying Feng, Xiaoli He, Mingxin Guo, Ying Tang, Guantong Qi, Qian Huang, Wenran Ma, Hong Chen, Yifan Qin, Ruiqi Li, Jin Wang & Yu Liu.
