# Supplementary material for: Quantifying the mRNA epitranscriptome reveals epitranscriptome signatures and roles in cancer
Source: Cell Mol Life Sci. 2025 Jul 28;82(1):290. doi: 10.1007/s00018-025-05805-7 (PMC12304408; doi:10.1007/s00018-025-05805-7)
Supplement: Supplementary file 1 — (DOCX 14.2 MB) [file 18_2025_5805_MOESM1_ESM.docx]

**Supplementary Materials**

**Quantifying the mRNA epitranscriptome** **reveals epitranscriptome signatures and roles in cancer**

Ying Feng, Xiaoli He, Mingxin Guo, Ying Tang, Guantong Qi, Qian Huang, Wenran Ma, Hong Chen, Yifan Qin, Ruiqi Li, Jin Wang and Yu Liu

**Contents:**

• Figure S1: Bioanalyzer analysis of total RNA using the RNA 6000 Pico LabChips.

• Figure S2: Bioanalyzer analysis of poly(A) RNA using the RNA 6000 Pico LabChips.

• Figure S3: Calibration curves for the quantification of modified nucleosides.

• Figure S4: Quantification of modified nucleosides in mRNA from WPMY-1 and LNCAP clone FGC.

• Figure S5: Quantification of modified nucleosides in mRNA from QSG-7701 and SNU-182.

• Figure S6: Quantification of modified nucleosides in mRNA from Hs 578Bst and BT-20.

• Figure S7: Quantification of modified nucleosides in mRNA from HeLa, DDP-treated HeLa, and DDP-resistant HeLa.

• Figure S8: Quantification of modified nucleosides in mRNA from HeLa, PTX-treated HeLa, and PTX-resistant HeLa.

• Figure S9: Quantification of modified nucleosides in mRNA from HeLa cells after knocking down the m^1^A modification enzymes *TRMT6*, *TRMT61A*, *TRMT6-61A*, *TRMT10C*, and the demodification enzyme *ALKBH3*.

• Figure S10: Flow cytometry analysis of the impact on the cell cycle after 72h of knockdown of m^1^A regulatory enzymes *TRMT10C*, *ALKBH3*, *TRMT6*, *TRMT61A*, and *TRMT6-61A*.

• Figure S11: Transcriptomic and proteomic analysis of *ALKBH3* treated HeLa cells.

• Figure S12: Transcriptomic and proteomic analysis of *TRMT6-61A* treated HeLa cells.

• Figure S13: Transcriptomic and proteomic analysis of *TRMT6* treated HeLa cells.

• Figure S14: Transcriptomic and proteomic analysis of *TRMT61A* treated HeLa cells.

• Figure S15: Transcriptomic and proteomic analysis of *TRMT10C* treated HeLa cells.

• Figure S16: Quantitative analysis using single-standard spike-in of m^3^C, m^4^C, m^5^C, and m^6^C.

• Figure S17: Product Spectrum of isobaric nucleosides.

• Table S1: siRNA used for transfection.

• Table S2: Primers used for qPCR analysis.

• Table S3: Cellular composition at different cell cycle phases after knocking down m^1^A regulatory enzymes in HeLa cells.

• Table S4: Detection limits of known and potentially existing modified nucleosides.

• Table S5: Dynamic MRM parameters for ribonucleosides based on optimizer results.

**Figure S1.** Bioanalyzer analysis of total RNA using the RNA 6000 Pico LabChips. (a) total RNA from human prostate cancer cell LNCAP clone FGC, (b) total RNA from human normal prostate stromal immortalized cell WPMY-1, (c) total RNA from human liver cancer cell SNU-182, (d) total RNA from human normal hepatocyte QSG-7701, (e) total RNA from human breast cancer cell BT-20, (f) total RNA from human normal breast cell Hs 578Bst total RNA, (g) total RNA from HeLa cells, (h) total RNA from HeLa cells treated with the anticancer drug cisplatin (DDP), (i) total RNA from HeLa cells treated with the anticancer drug paclitaxel (PTX), (j) total RNA from HeLa cells resistant to cisplatin (DDP), (k) total RNA from HeLa cells resistant to paclitaxel (PTX), (l) total RNA from HeLa cells after transfection with NC-siRNA, (m) total RNA from HeLa cells after knocking down the m^1^A modification enzyme TRMT6, (n) total RNA from HeLa cells after knocking down the m^1^A modification enzyme TRMT61A, (o) total RNA from HeLa cells after knocking down the m^1^A modification enzyme TRMT6-61A, (p) total RNA from HeLa cells after knocking down the m^1^A modification enzyme TRMT10C, (q) total RNA from HeLa cells after knocking down the m^1^A modification enzyme ALKBH3, (r) 6000 Pico RNA ladder.


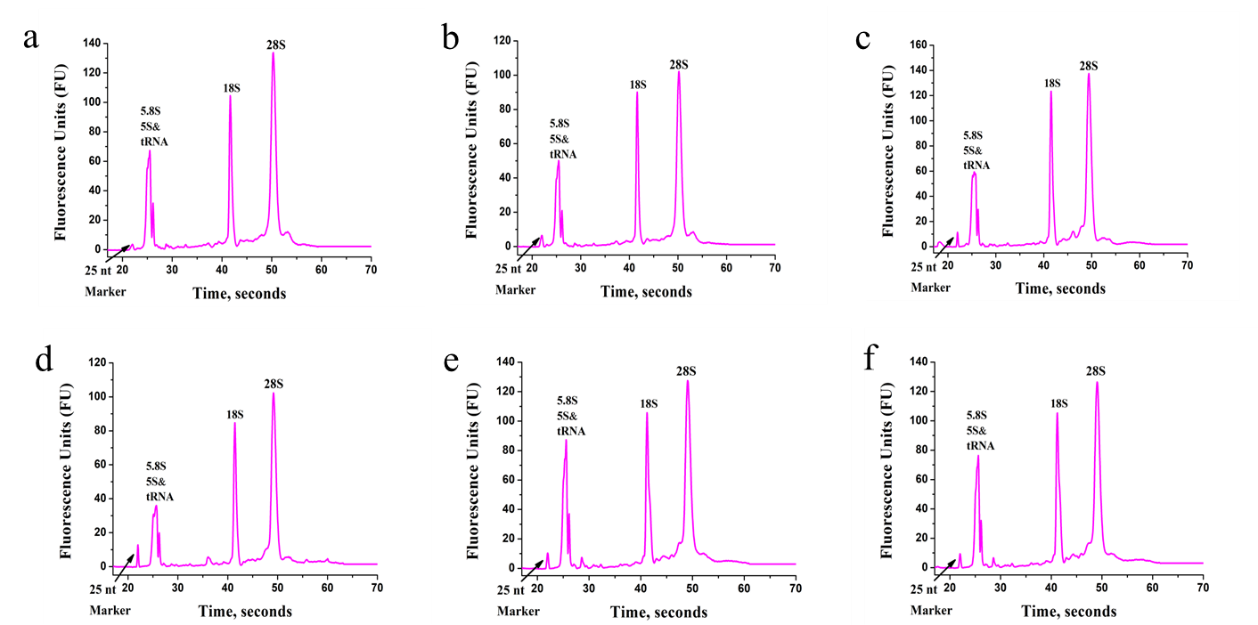

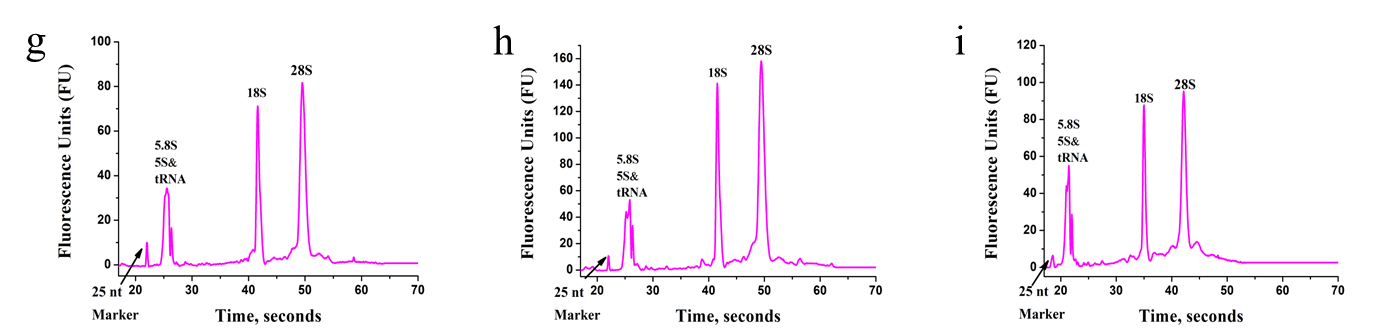


**Figure S1, continued**


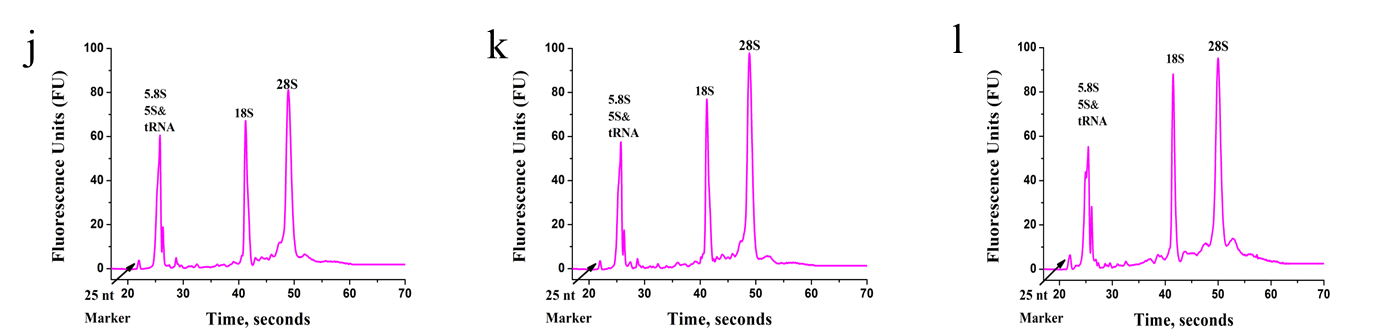

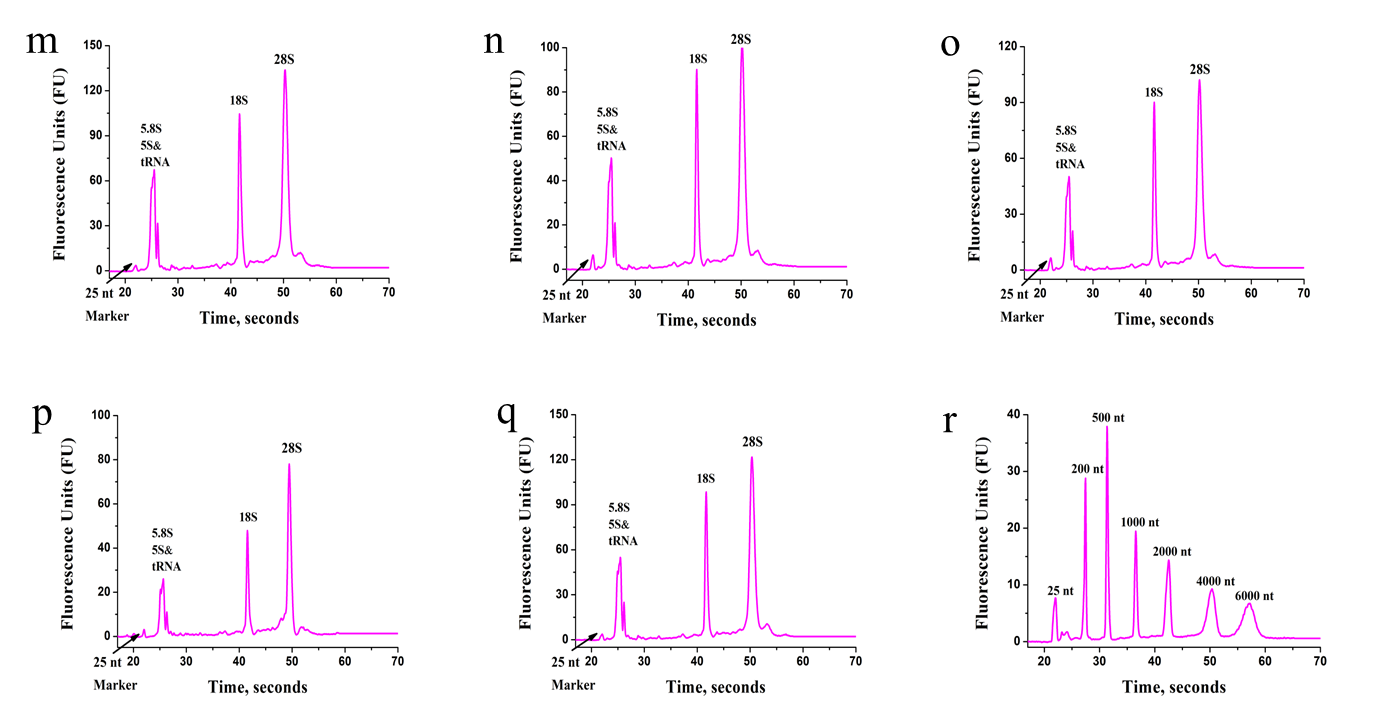


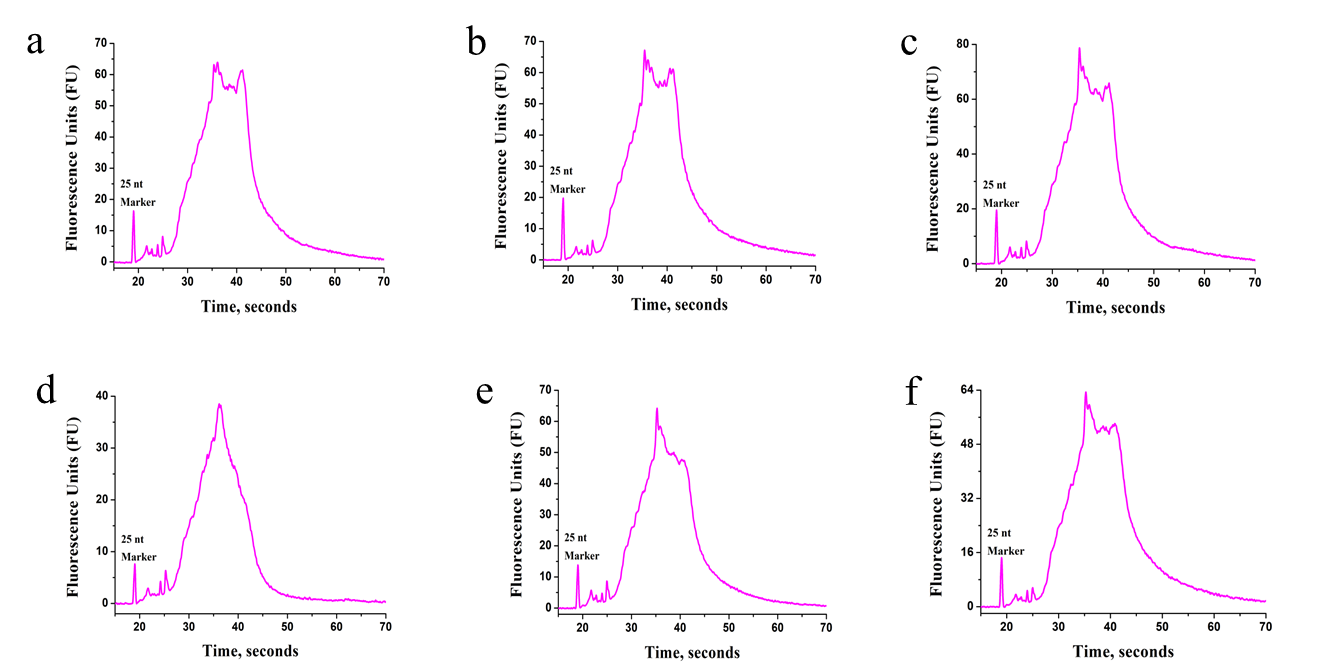
**Figure S2.** Bioanalyzer analysis of rRNA-depleted poly(A) RNA using the RNA 6000 Pico LabChips. (a) rRNA-depleted poly(A) RNA from human prostate cancer cell LNCAP clone FGC, (b) rRNA-depleted poly(A) RNA from human normal prostate stromal immortalized cell WPMY-1, (c) rRNA-depleted poly(A) RNA from human liver cancer cell SNU-182, (d) rRNA-depleted poly(A) RNA from human normal hepatocyte QSG-7701, (e) rRNA-depleted poly(A) RNA from human breast cancer cell BT-20, (f) rRNA-depleted poly(A) RNA from human normal breast cell Hs 578Bst, (g) rRNA-depleted poly(A) RNA from HeLa cells, (h) rRNA-depleted poly(A) RNA from HeLa cells treated with the anticancer drug cisplatin (DDP), (i) rRNA-depleted poly(A) RNA from HeLa cells treated with the anticancer drug paclitaxel (PTX), (j) rRNA-depleted poly(A) RNA from HeLa cells resistant to cisplatin (DDP), (k) rRNA-depleted poly(A) RNA from HeLa cells resistant to paclitaxel (PTX), (l) rRNA-depleted poly(A) RNA from HeLa cells after transfection with NC-siRNA, (m) rRNA-depleted poly(A) RNA from HeLa cells after knocking down the m^1^A modification enzyme TRMT6, (n) rRNA-depleted poly(A) RNA from HeLa cells after knocking down the m^1^A modification enzyme TRMT61A, (o) rRNA-depleted poly(A) RNA from HeLa cells after knocking down the m^1^A modification enzyme TRMT6-61A, (p) rRNA-depleted poly(A) RNA from HeLa cells after knocking down the m^1^A modification enzyme TRMT10C, (q) rRNA-depleted poly(A) RNA from HeLa cells after knocking down the m^1^A modification enzyme ALKBH3, (r) 6000 Pico RNA ladder.

**Figure S2, continued**


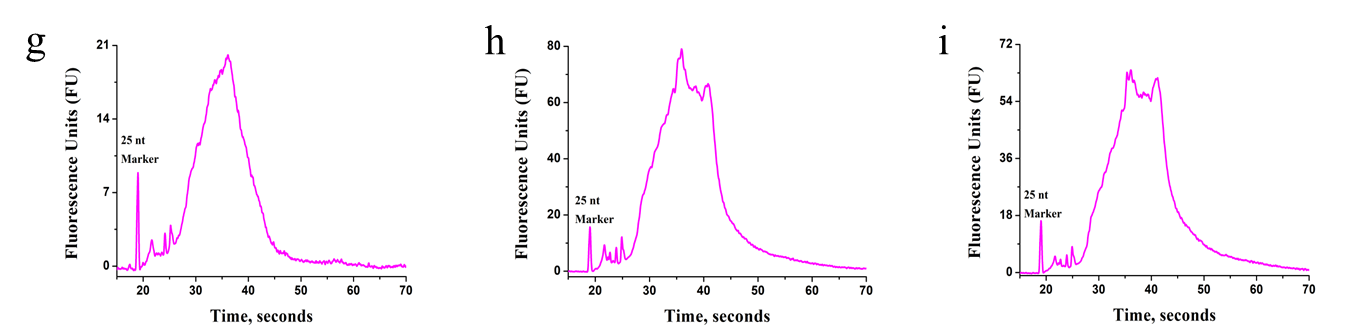

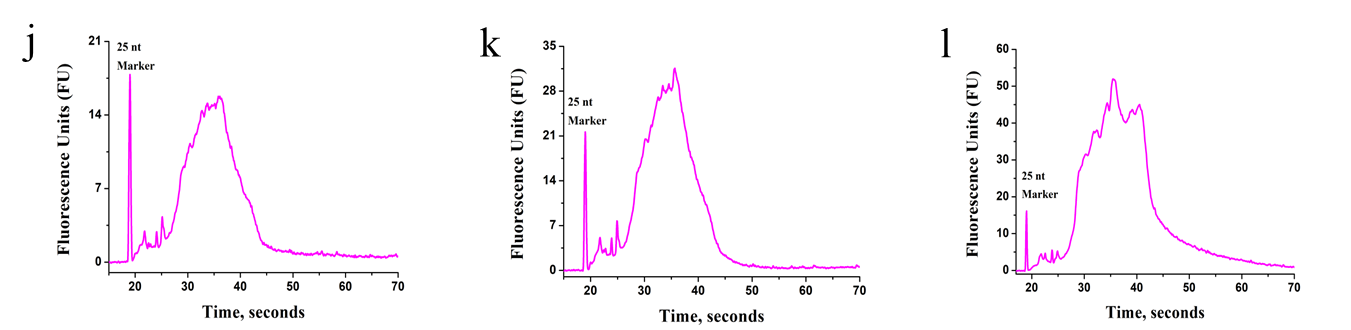

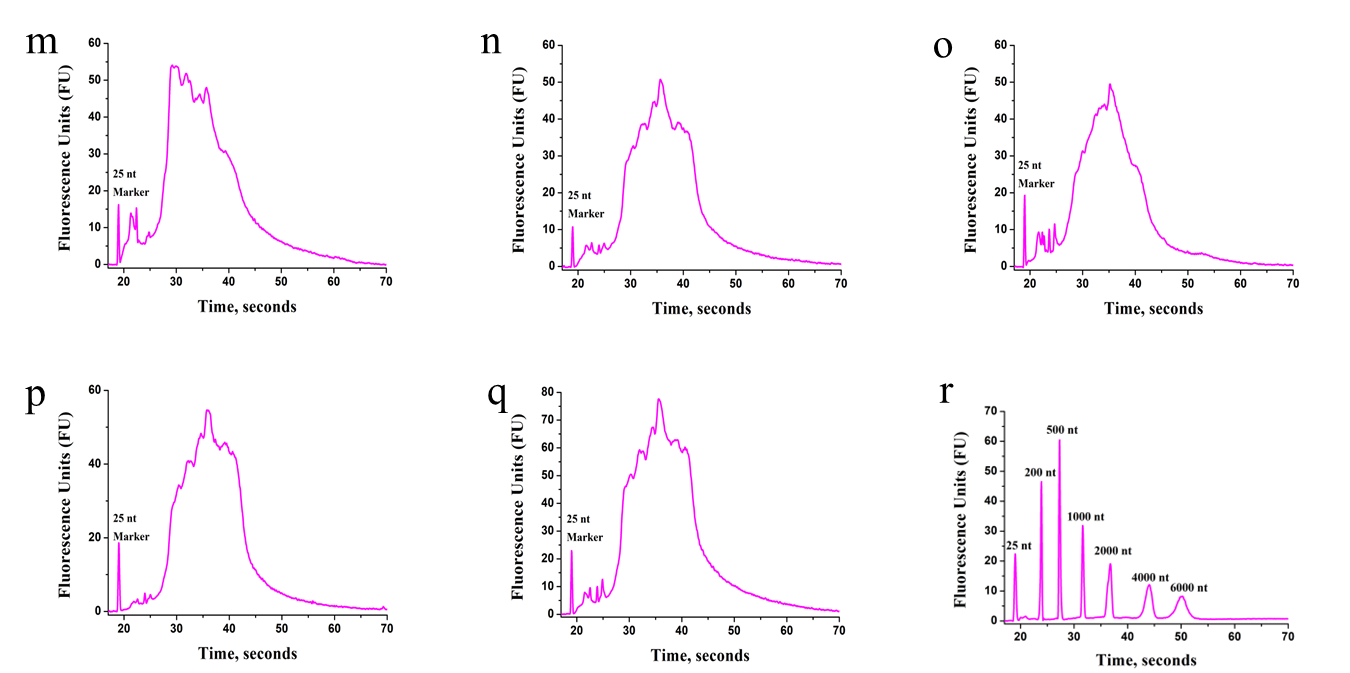


**Figure S3.** Calibration curves for the quantification of modified nucleosides. (a-ah) Am, m^1^A, m^2^A, m^6^A, m^6^Am, m^8^A, m^6,6^A, ms^2^m^6^A, i^6^A, t^6^A, Um, m^3^Um, m^5^Um, hm^5^U, ncm^5^U, ψ, mchm^5^U, mnm^5^s^2^U, Cm, m^5^C, hm^5^C, ca^5^C, ac^4^C, Gm, m^1^G, m^2^G, m^6^G, m^7^G, m^2,2^G, m^2,7^G, m^2,2,7^G, D, I, imG-14。

**Figure S3, continued**


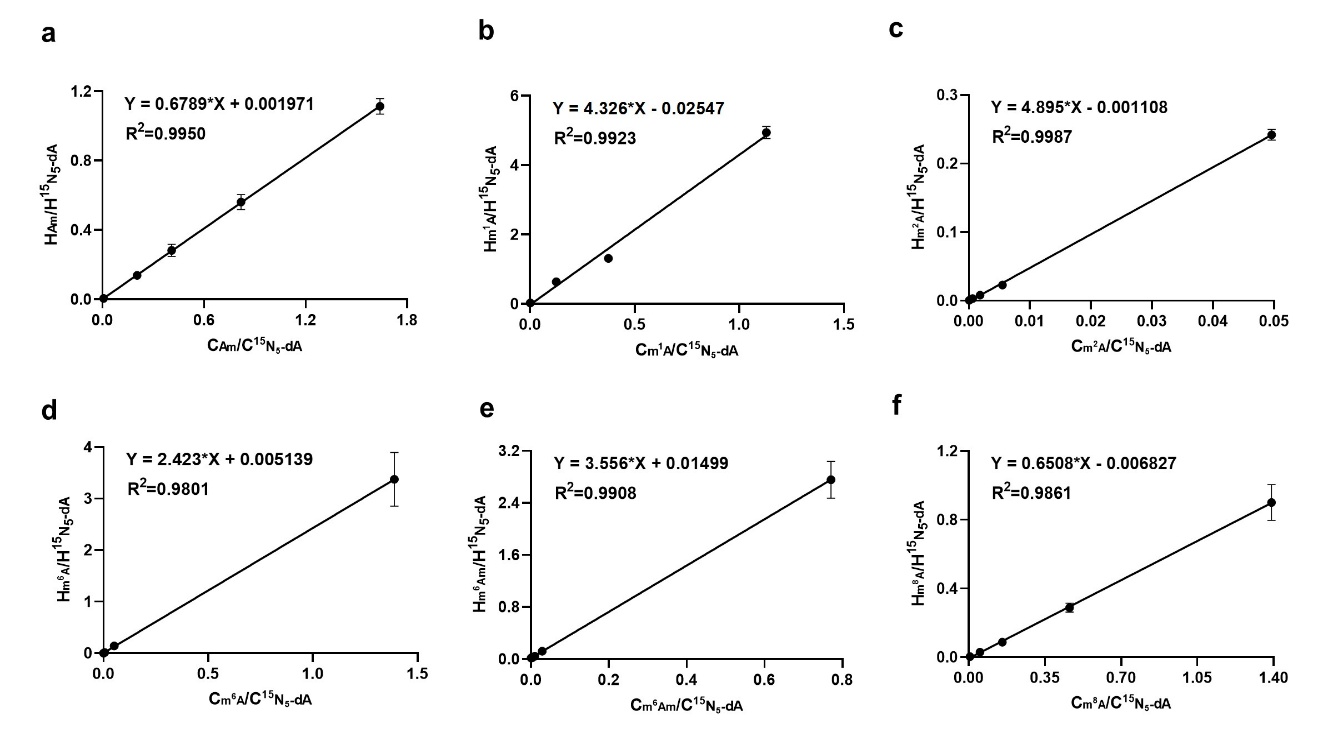

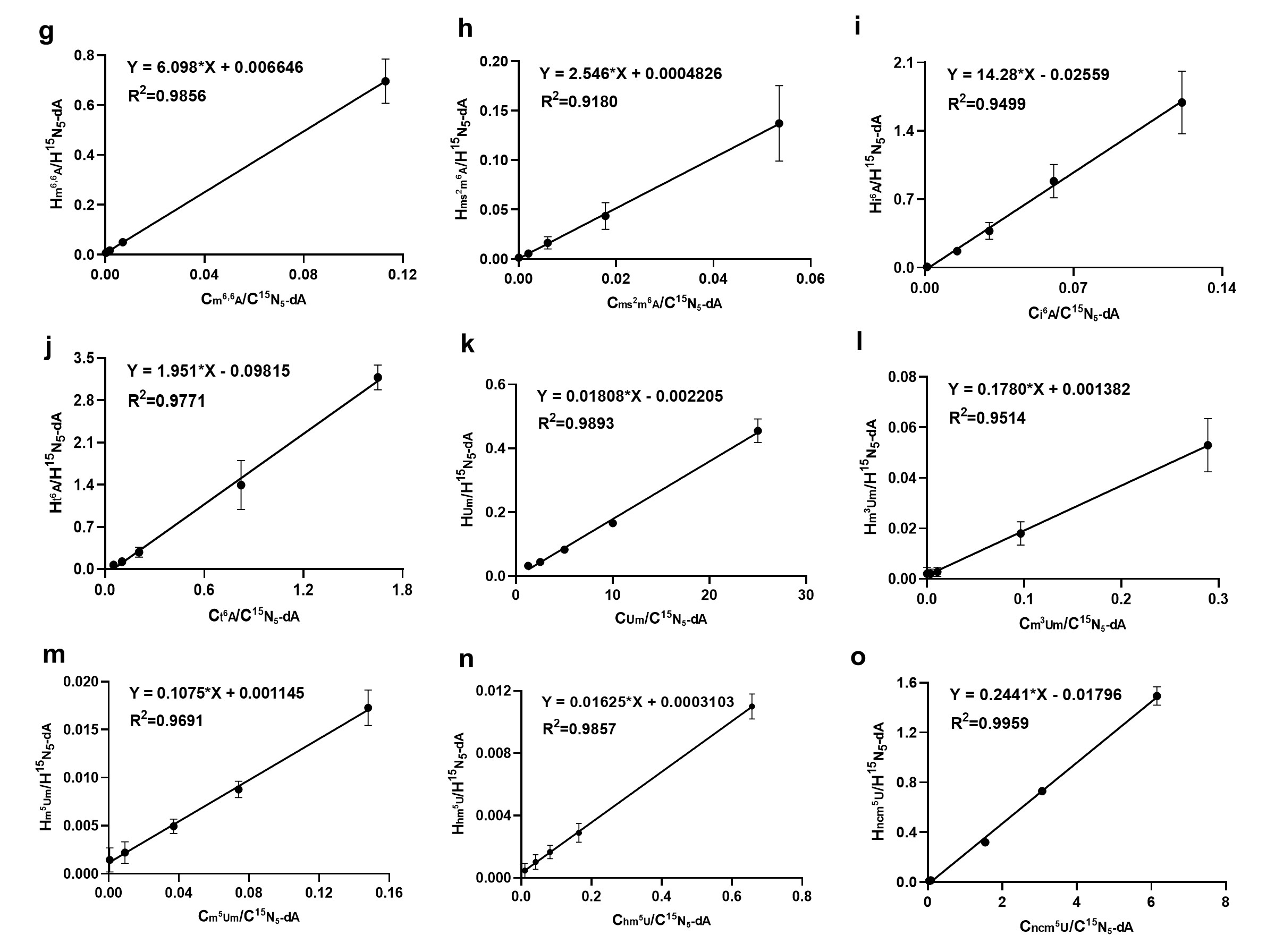

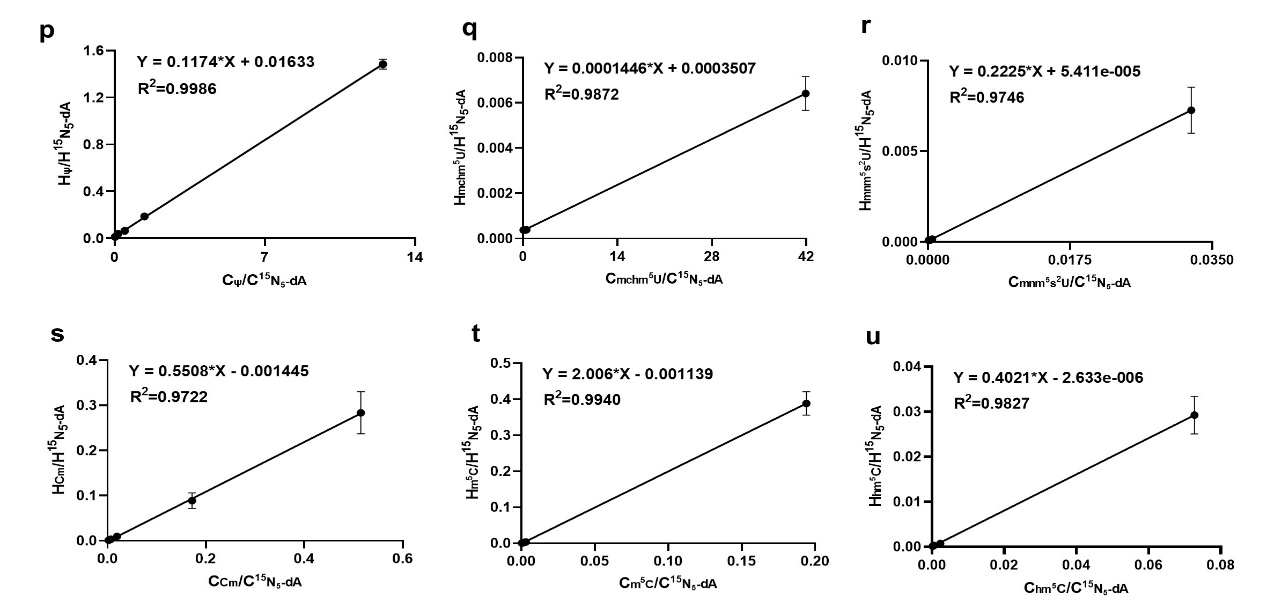

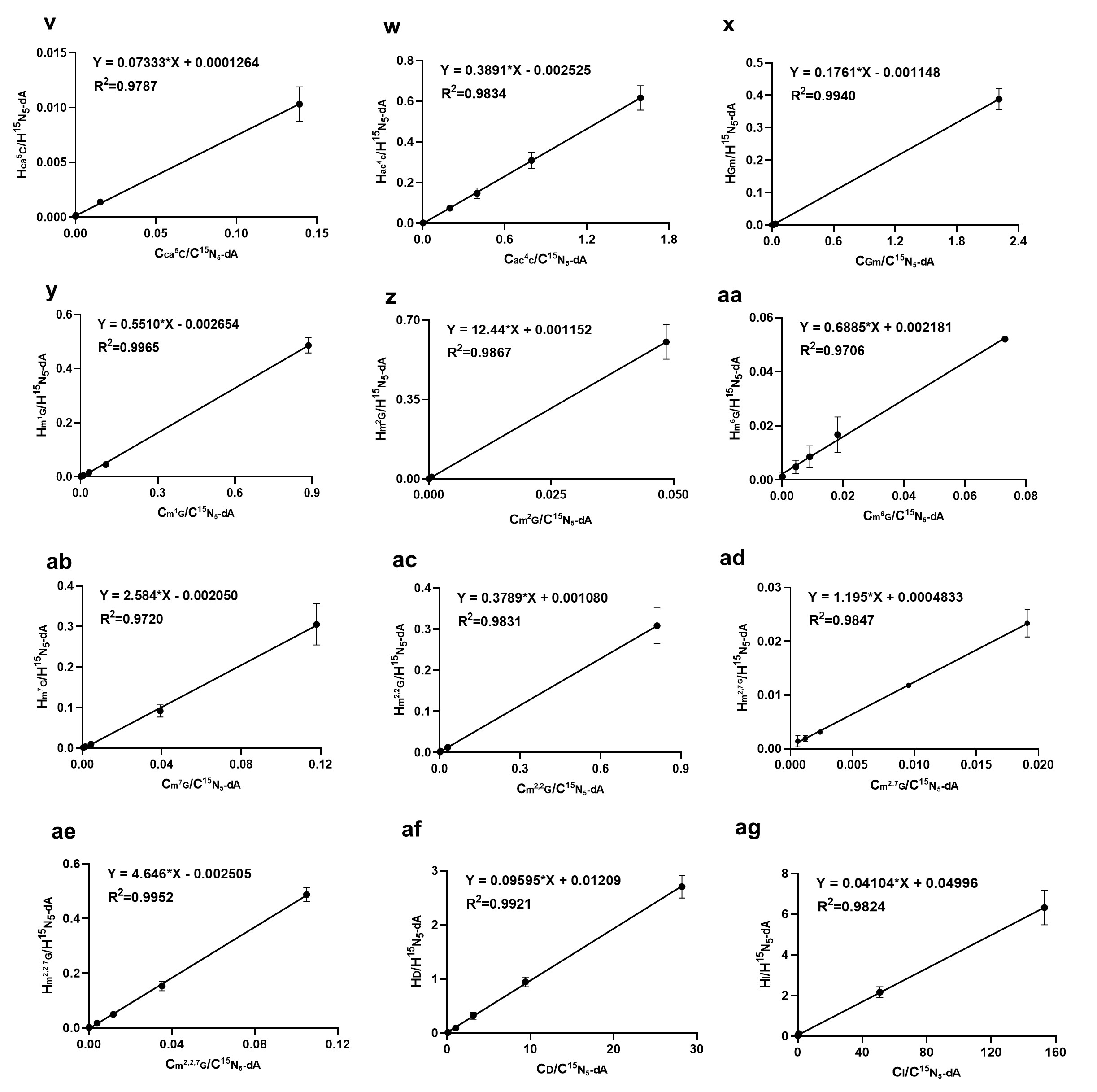

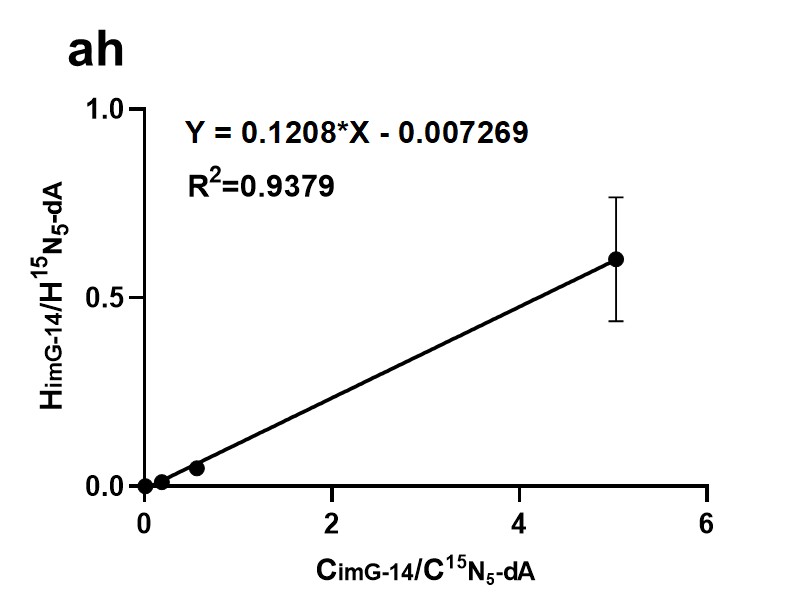


**Figure S4.** Quantification of the release of modified nucleosides during mRNA digestion of WPMY-1 and LNCAP clone FGC. (a-ah) Am, m^1^A, m^2^A, m^6^A, m^8^A, m^6^Am, m^6,6^A, i^6^A, ms^2^m^6^A, t^6^A, I, Gm, m^1^G, m^2^G, m^6^G, m^7^G, m^2,7^G, m^2,2^G, m^2,2,7^G, imG-14, Cm, m^5^C, ac^4^C, ca^5^C, hm^5^C, Um, m^3^Um, m^5^Um, mchm^5^U, ncm^5^U, mnm^5^s^2^U, hm^5^U, ψ, D. Values represent the mean ± SD of three biological experiments. "nd" indicates not detected. * p < 0.05, ** p < 0.01, *** p < 0.001.


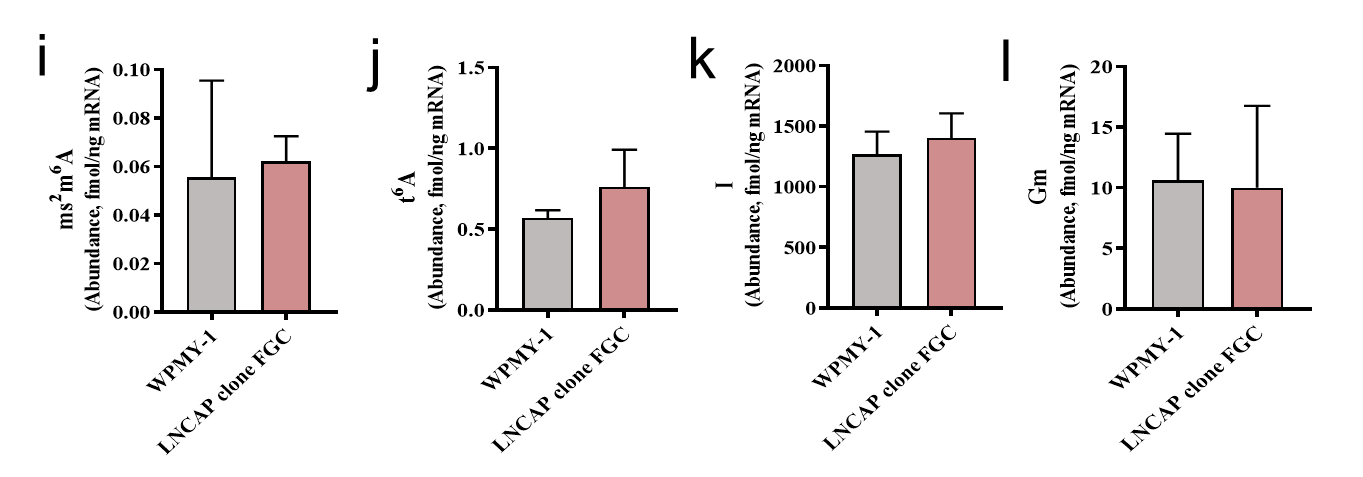

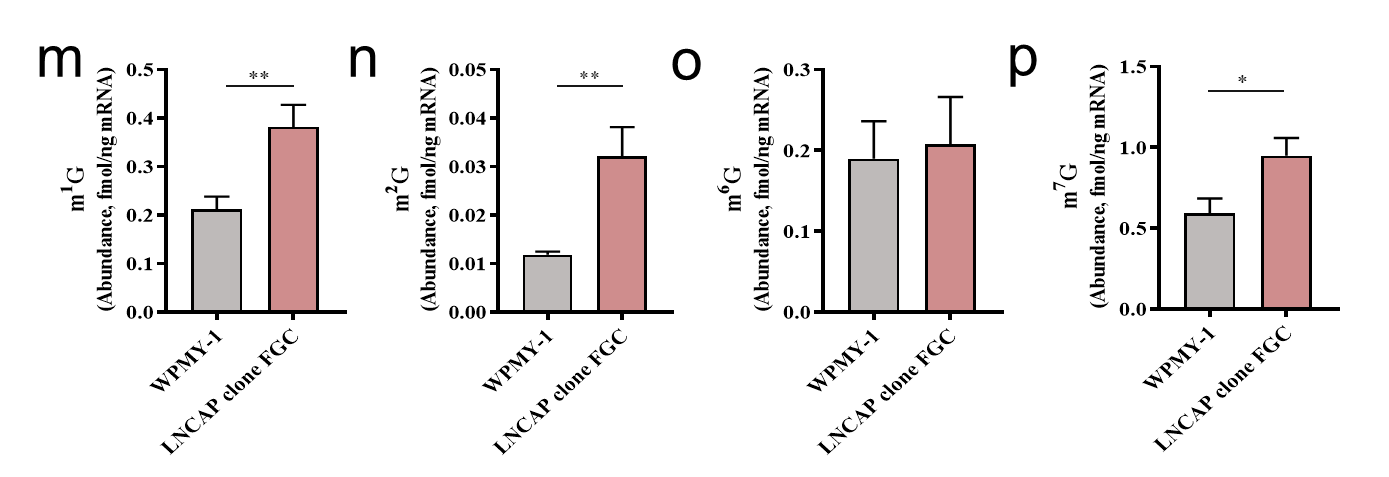

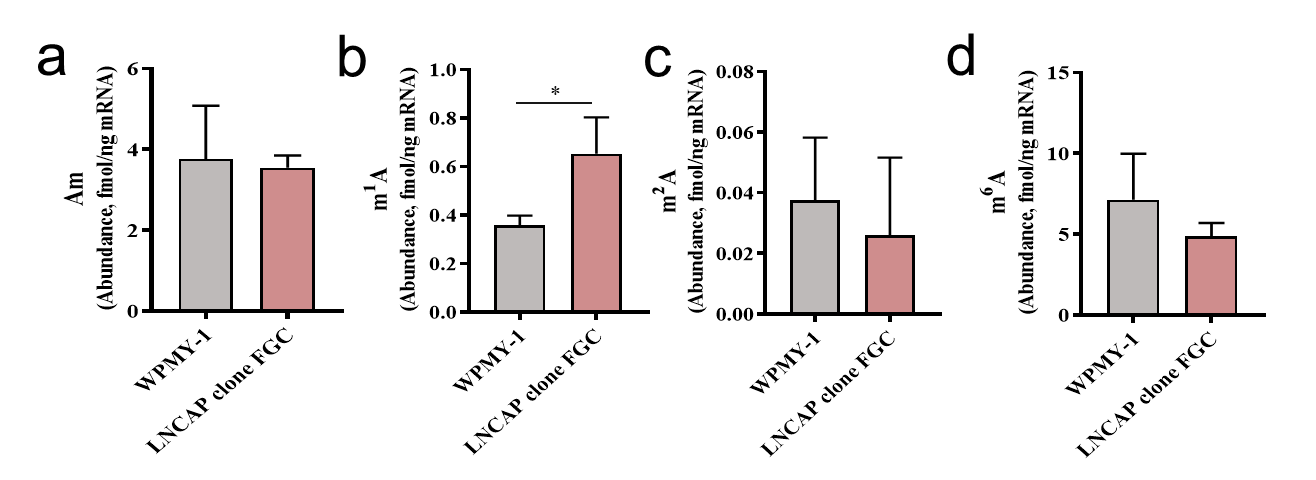

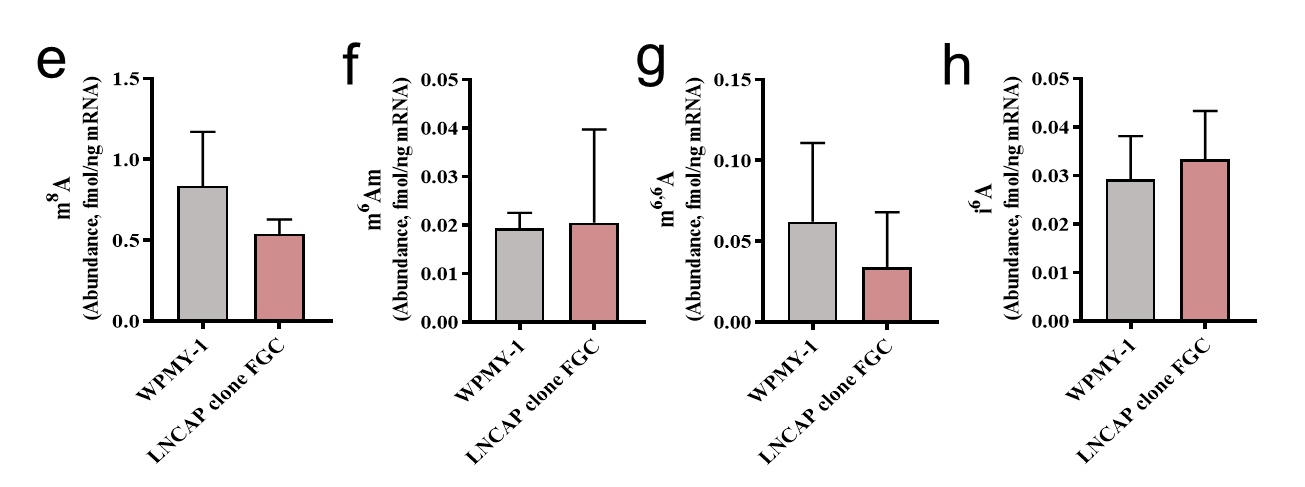


**Figure S4, continued**


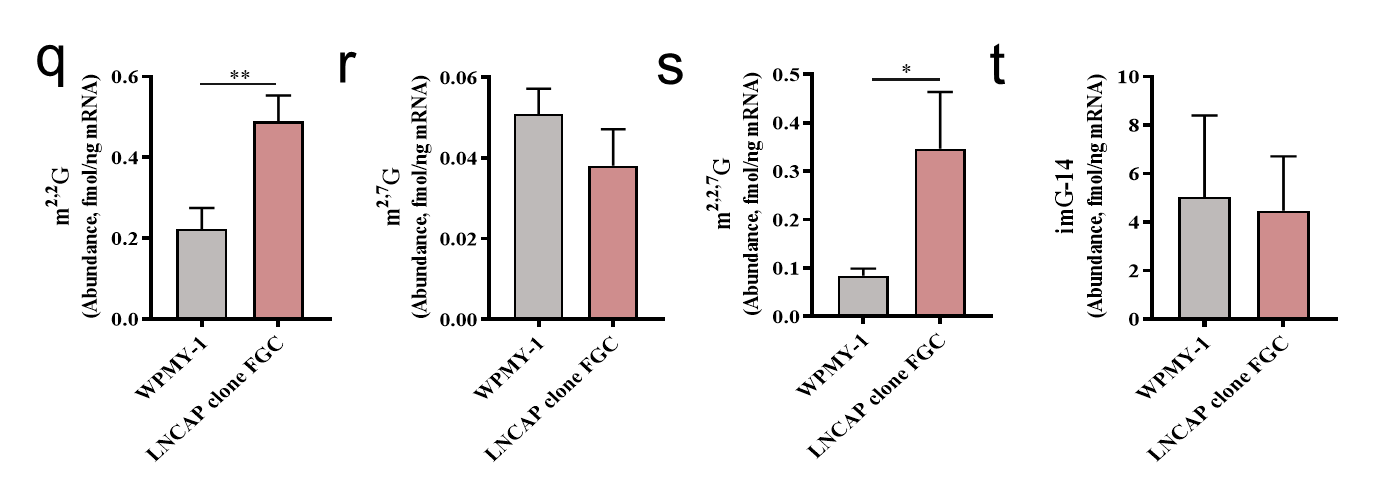

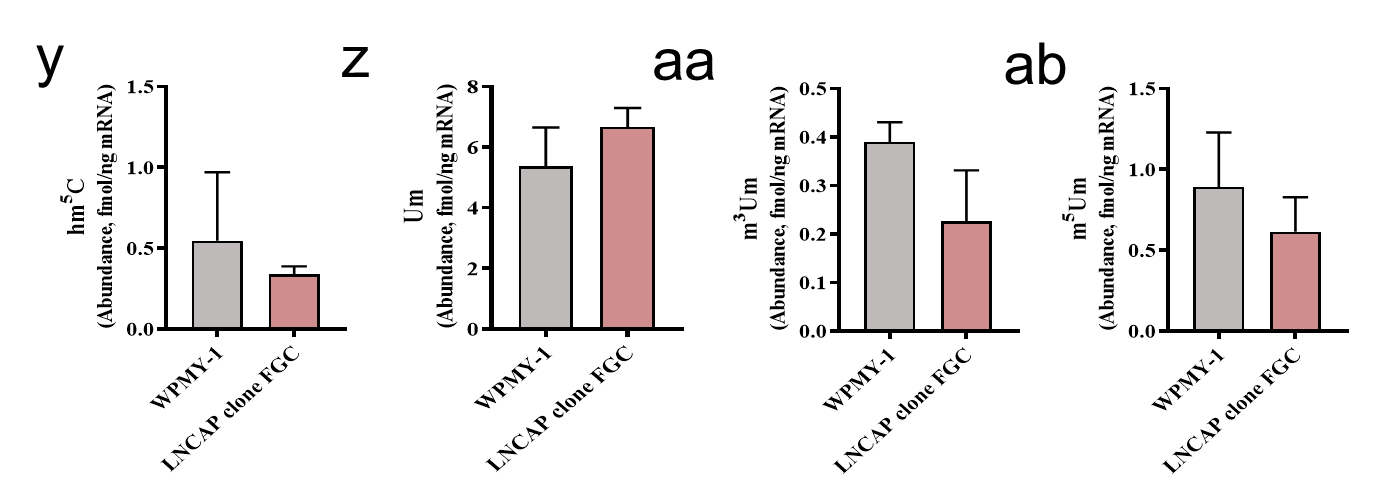

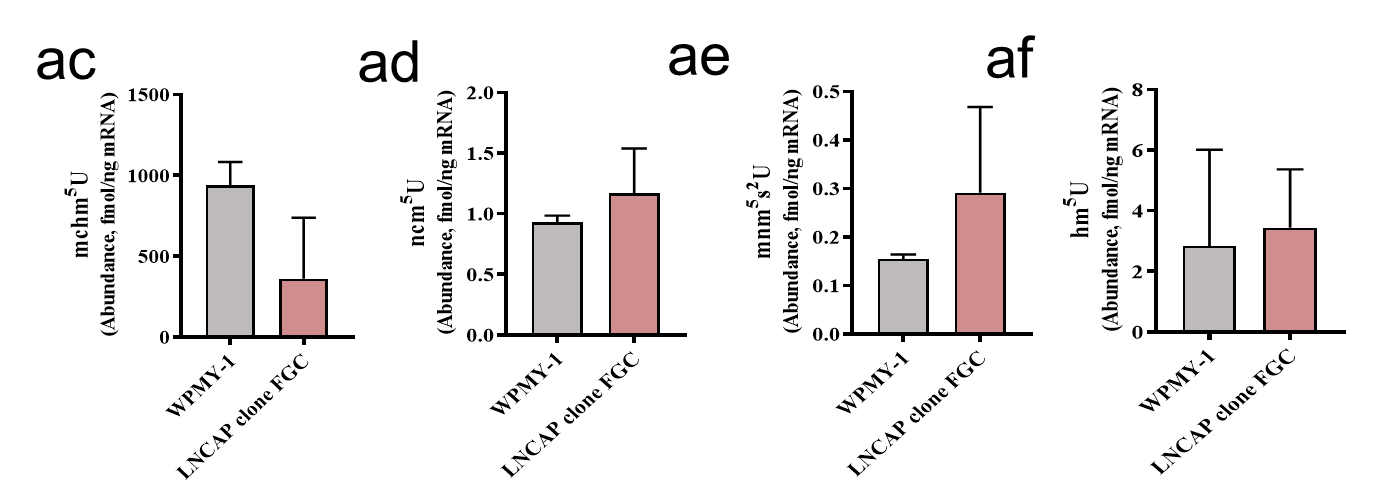

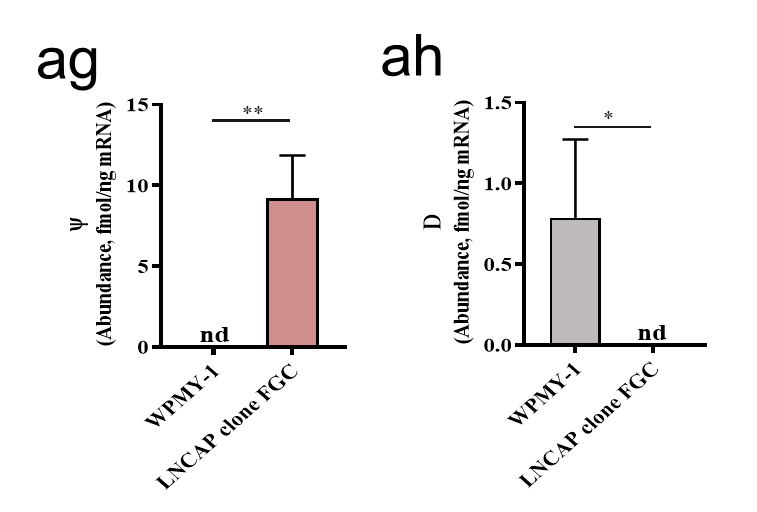

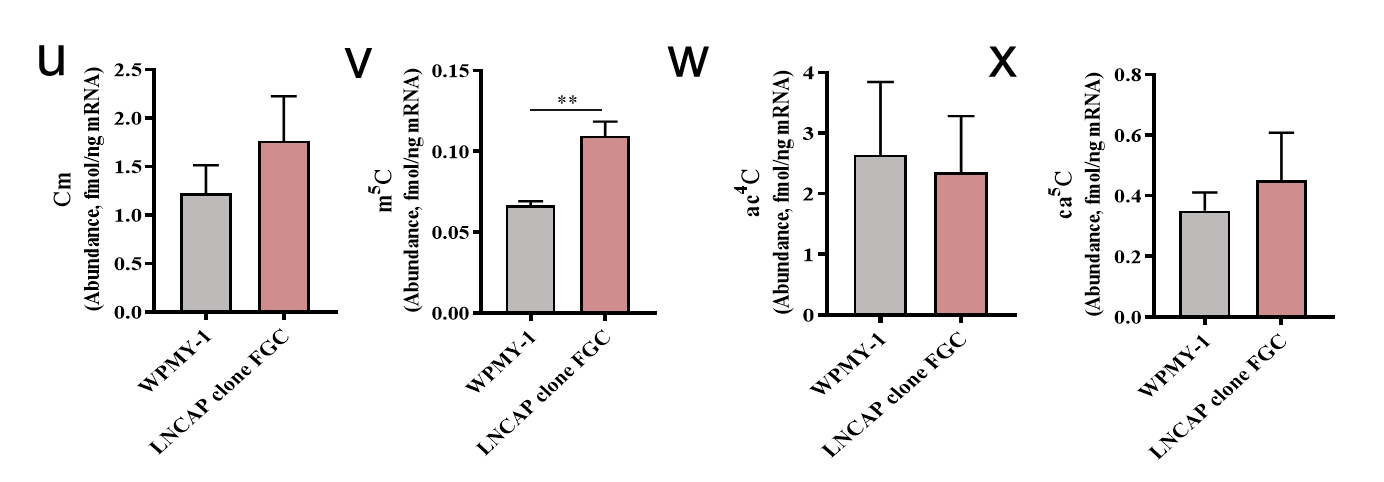


**Figure S5.** Quantification of the release of modified nucleosides during mRNA digestion of QSG-7701 and SNU-182. (a-ah) Am, m^1^A, m^2^A, m^6^A, m^8^A, m^6^Am, m^6,6^A, i^6^A, ms^2^m^6^A, t^6^A, I, Gm, m^1^G, m^2^G, m^6^G, m^7^G, m^2,7^G, m^2,2^G, m^2,2,7^G, imG-14, Cm, m^5^C, ac^4^C, ca^5^C, hm^5^C, Um, m^3^Um, m^5^Um, mchm^5^U, ncm^5^U, mnm^5^s^2^U, hm^5^U, ψ, D. Values represent the mean ± SD of three biological experiments. "nd" indicates not detected. * p < 0.05, ** p < 0.01, *** p < 0.001.


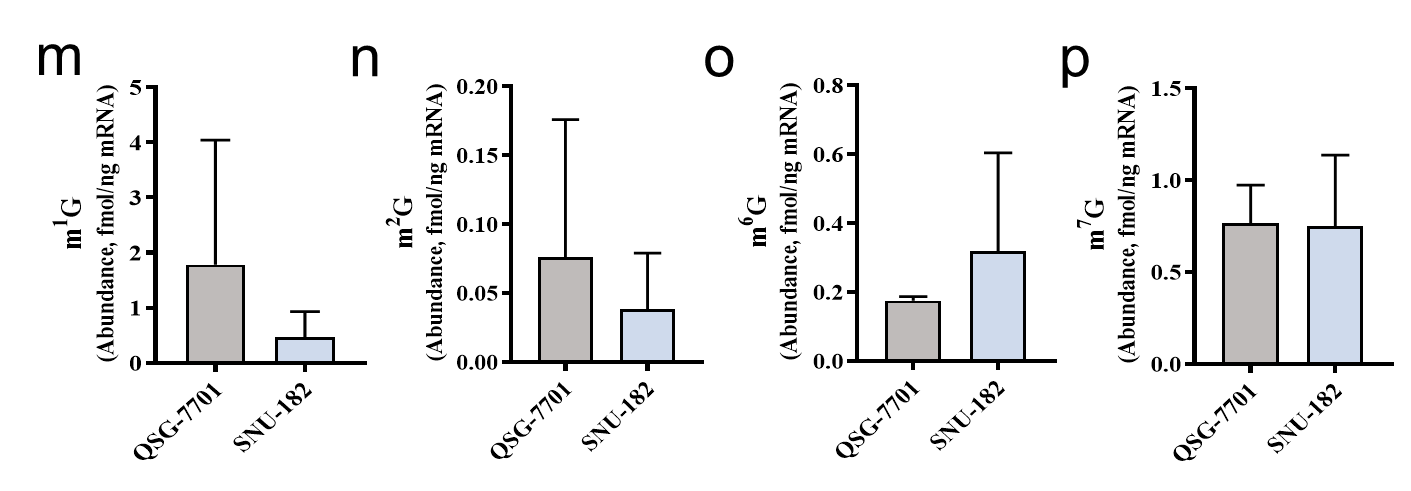

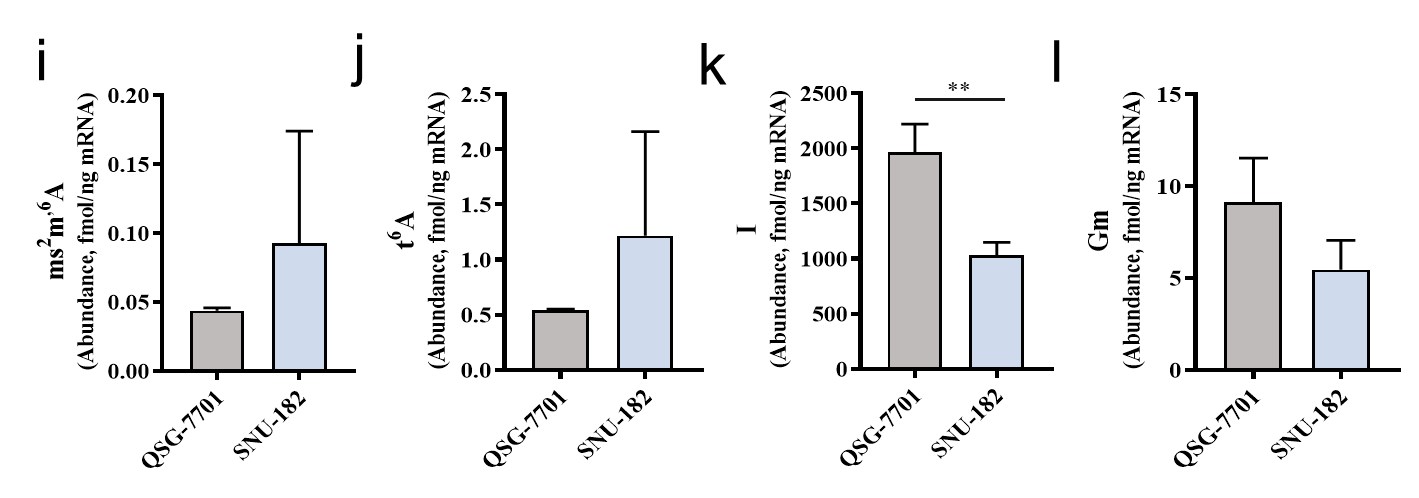

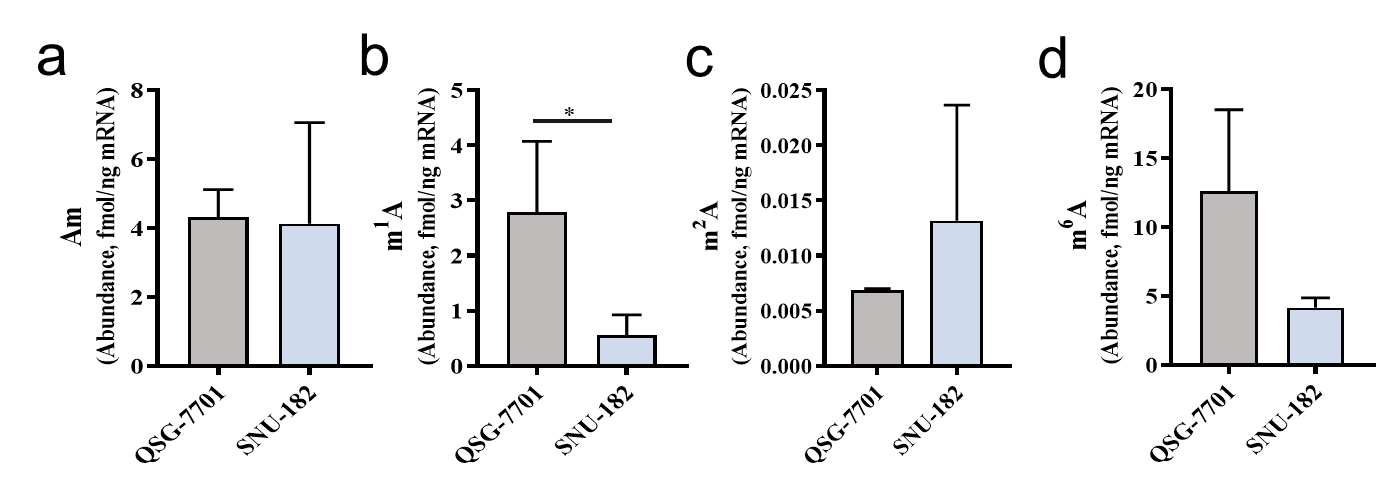

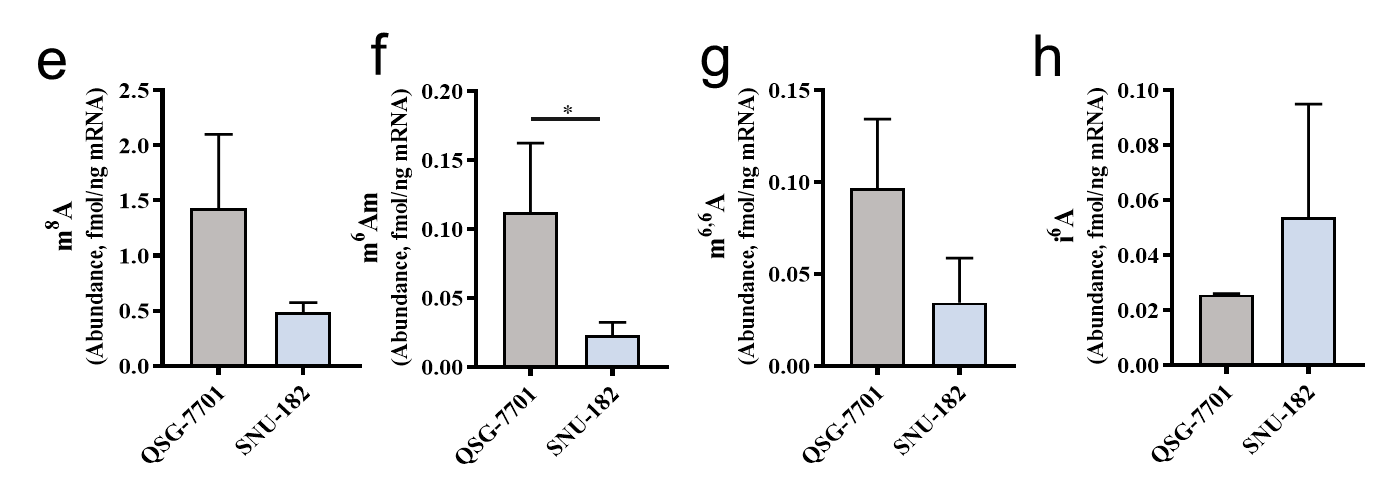


**Figure S5, continued**

**Figure S6.** Quantification of the release of modified nucleosides during mRNA digestion of Hs 578Bst and BT-20 (a-ag) Am, m^1^A, m^2^A, m^6^A, m^8^A, m^6^Am, m^6,6^A, i^6^A, ms^2^m^6^A, t^6^A, I, Gm, m^1^G, m^2^G, m^6^G, m^7^G, m^2,7^G, m^2,2^G, m^2,2,7^G, imG-14, Cm, m^5^C, ac^4^C, ca^5^C, hm^5^C, Um, m^3^Um, m^5^Um, mchm^5^U, ncm^5^U, mnm^5^s^2^U, hm^5^U, ψ. Values represent the mean ± SD of three biological experiments. "nd" indicates not detected. * p < 0.05, ** p < 0.01, *** p < 0.001.


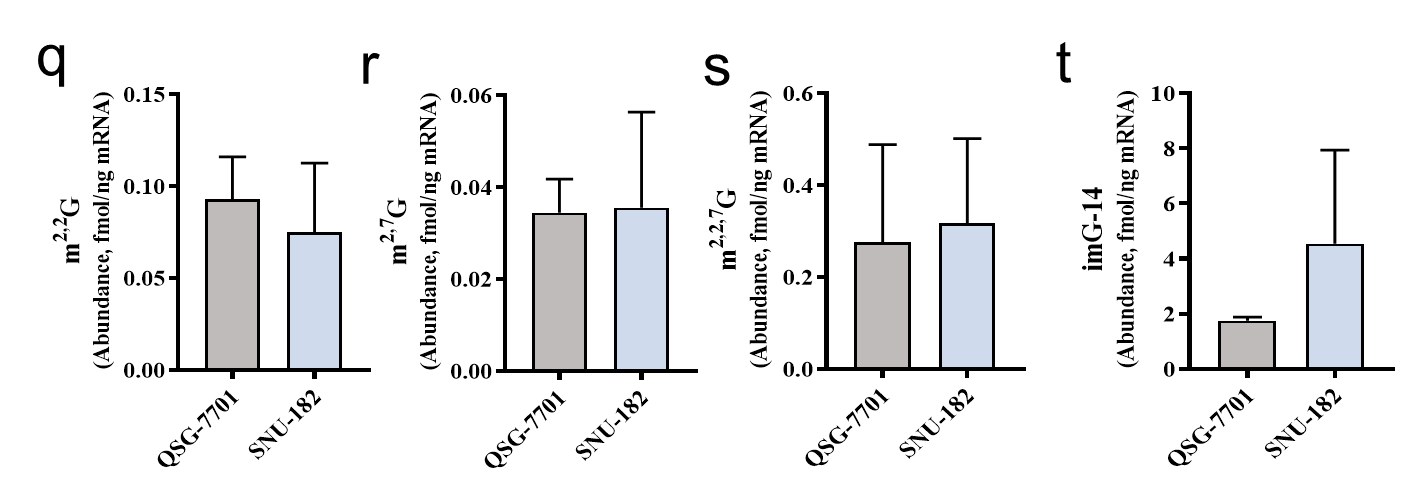

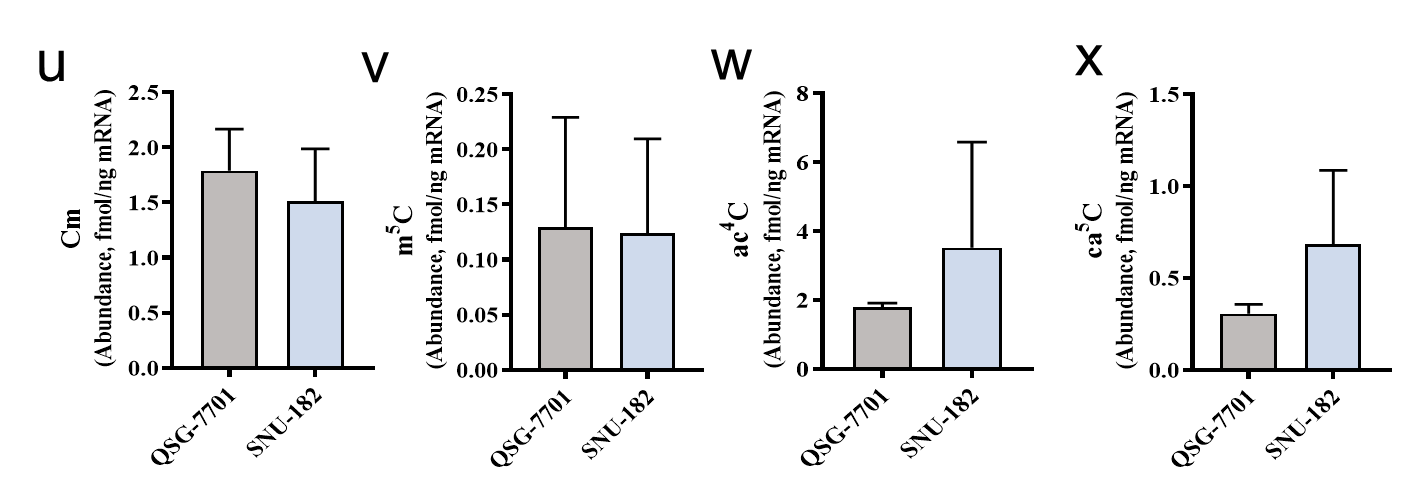

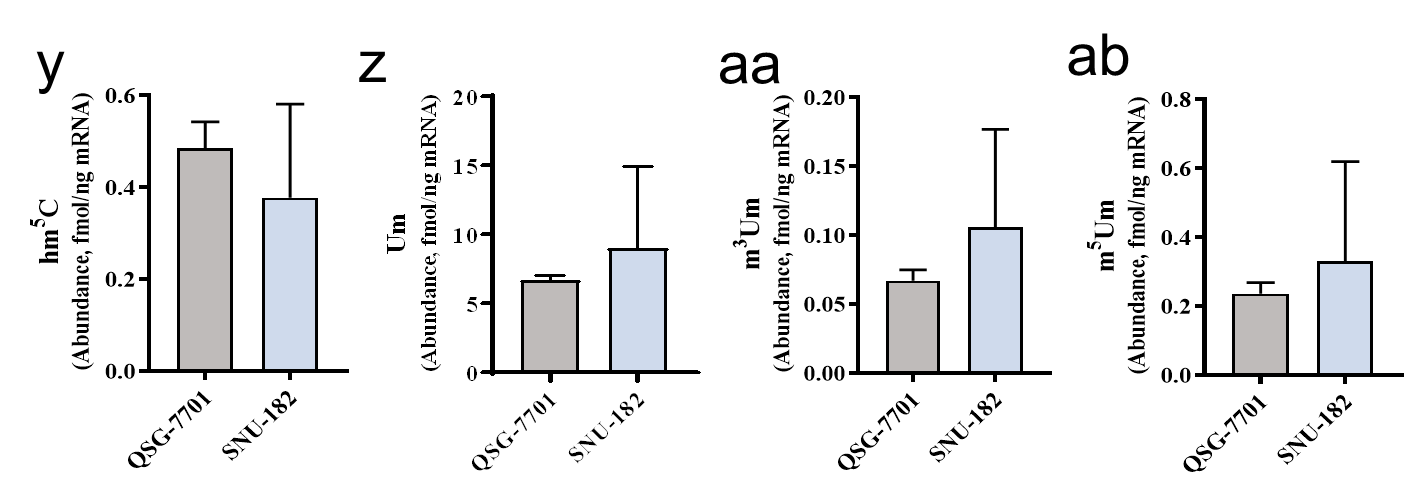

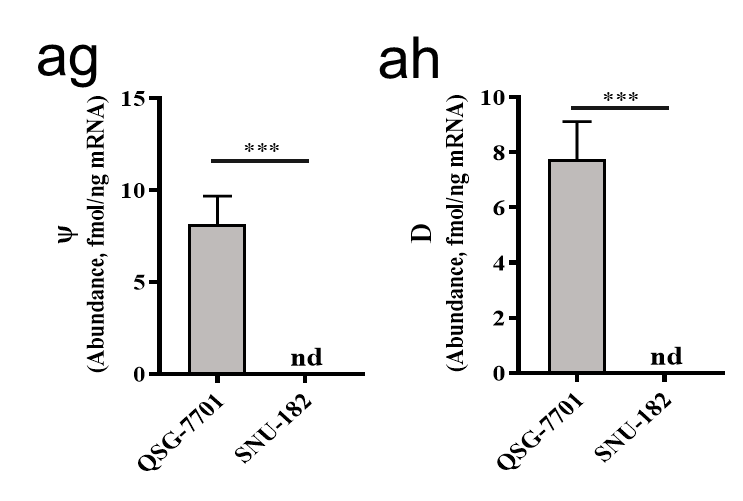

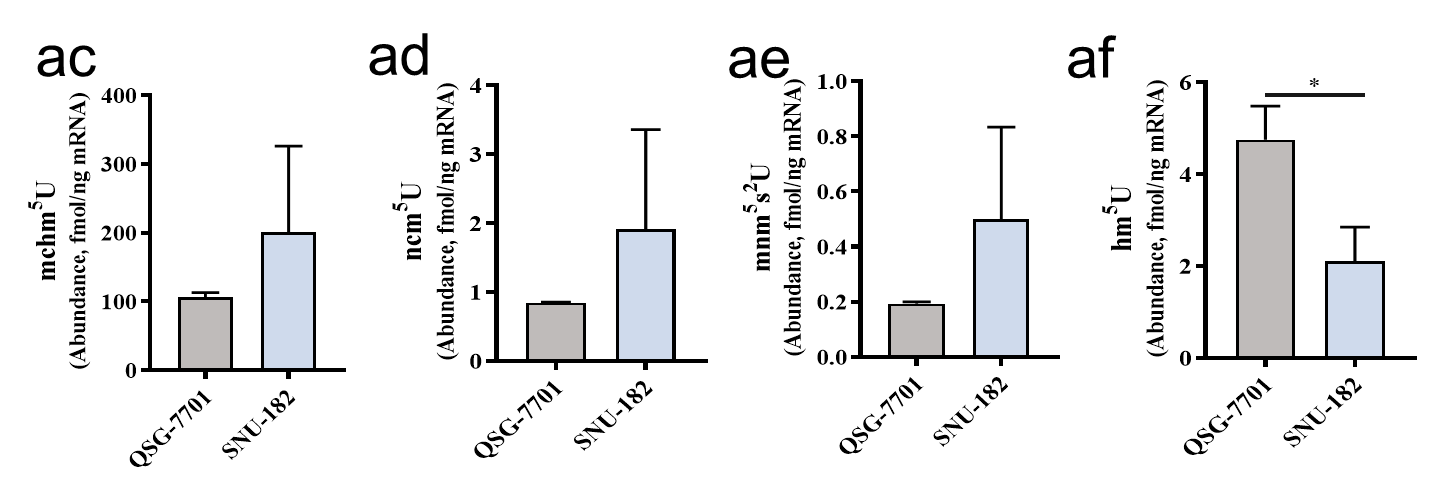

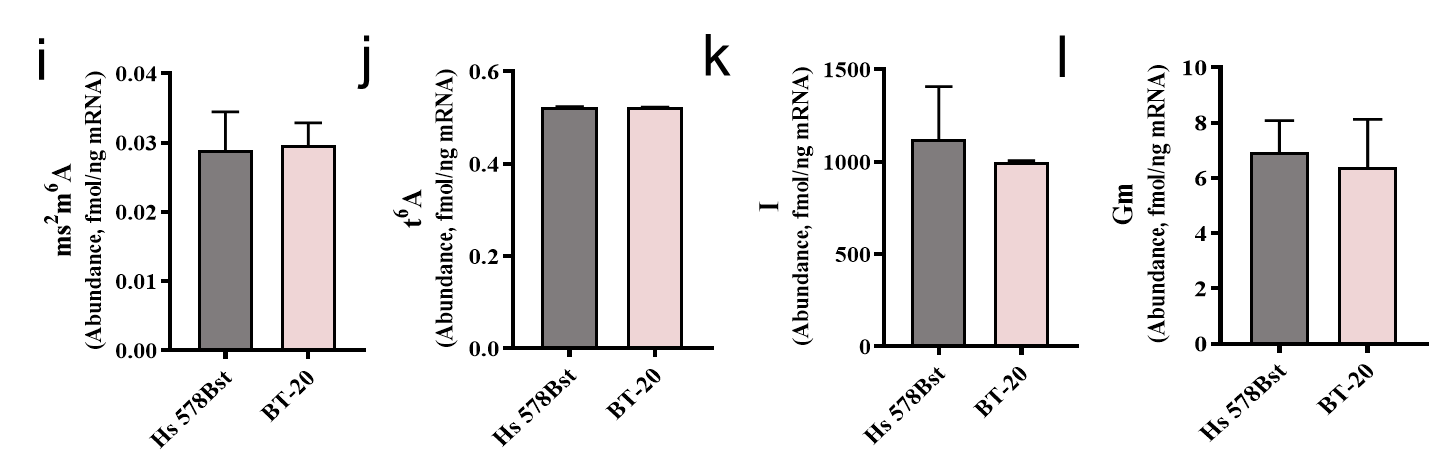

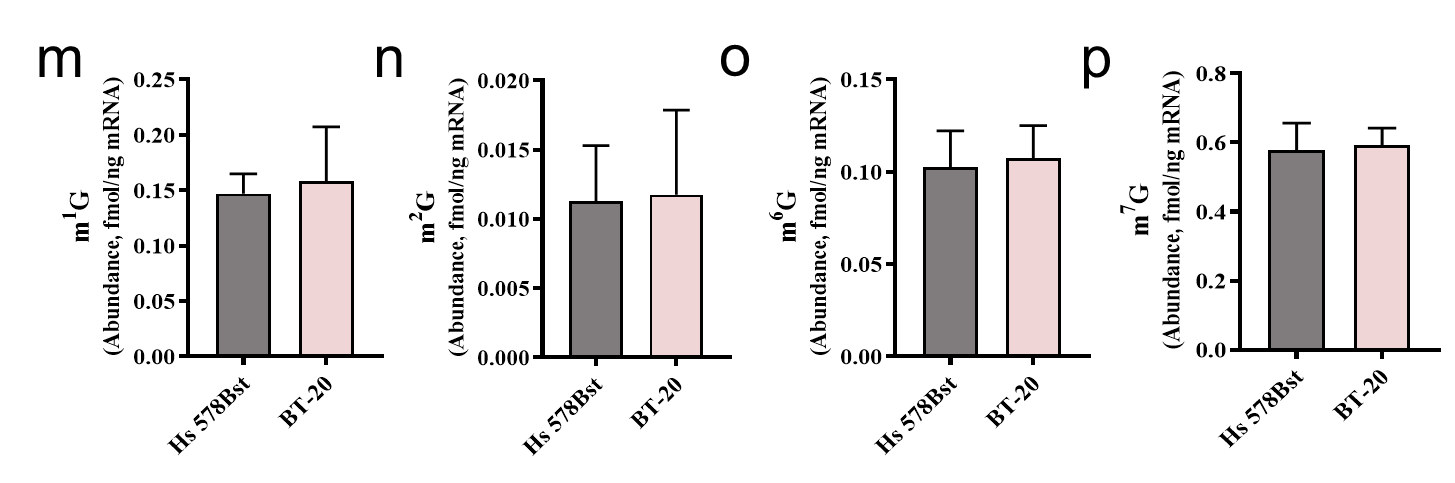

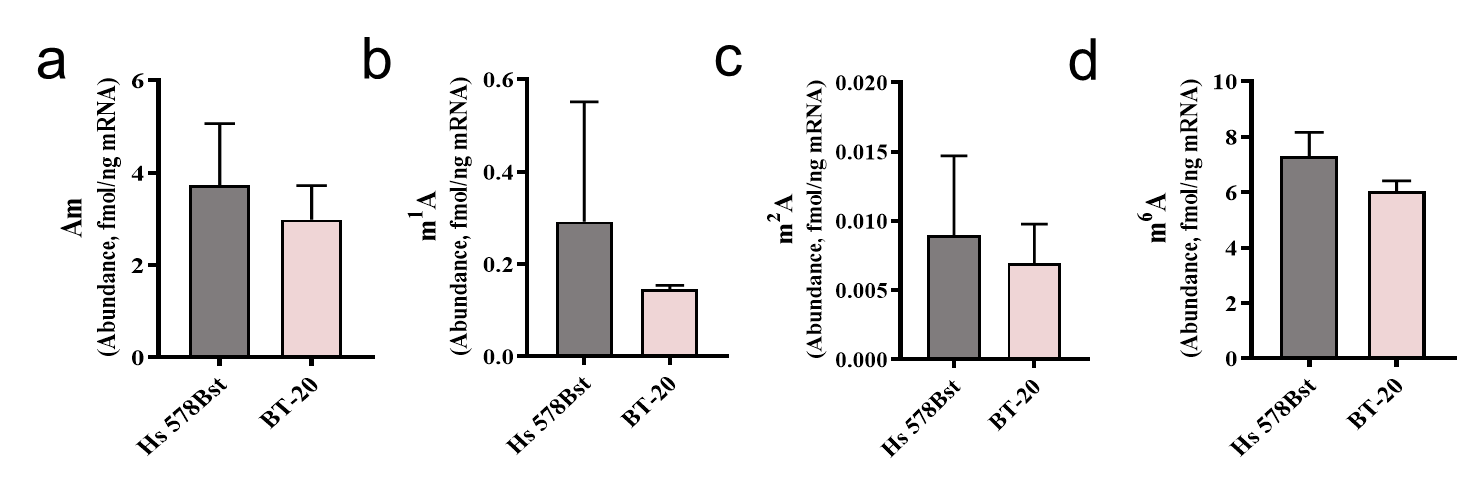

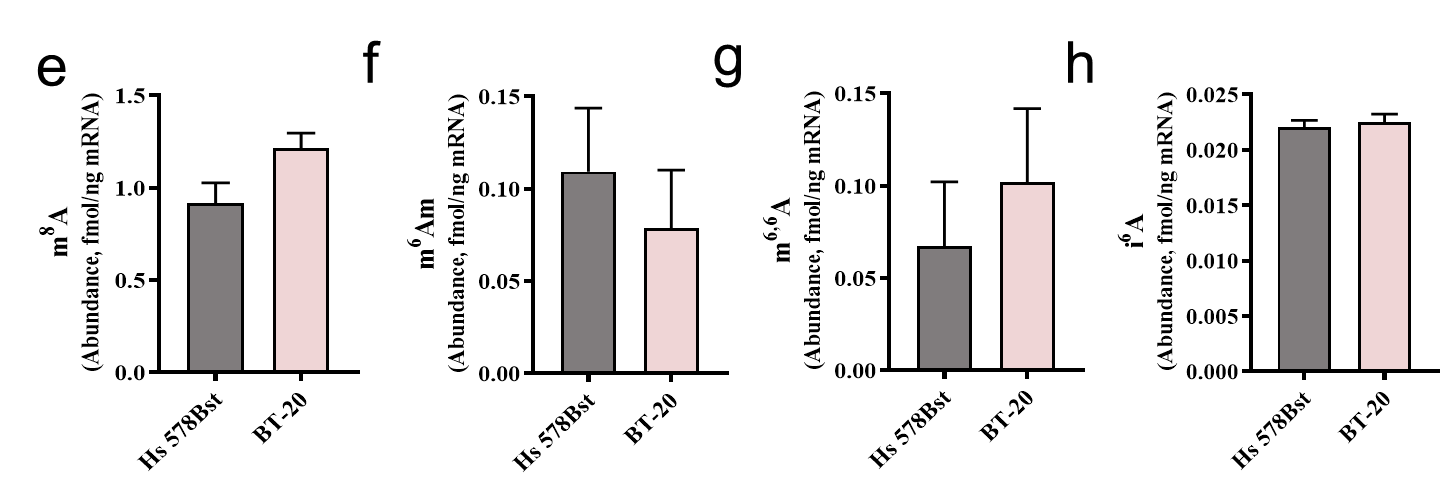


**Figure S6, continued**


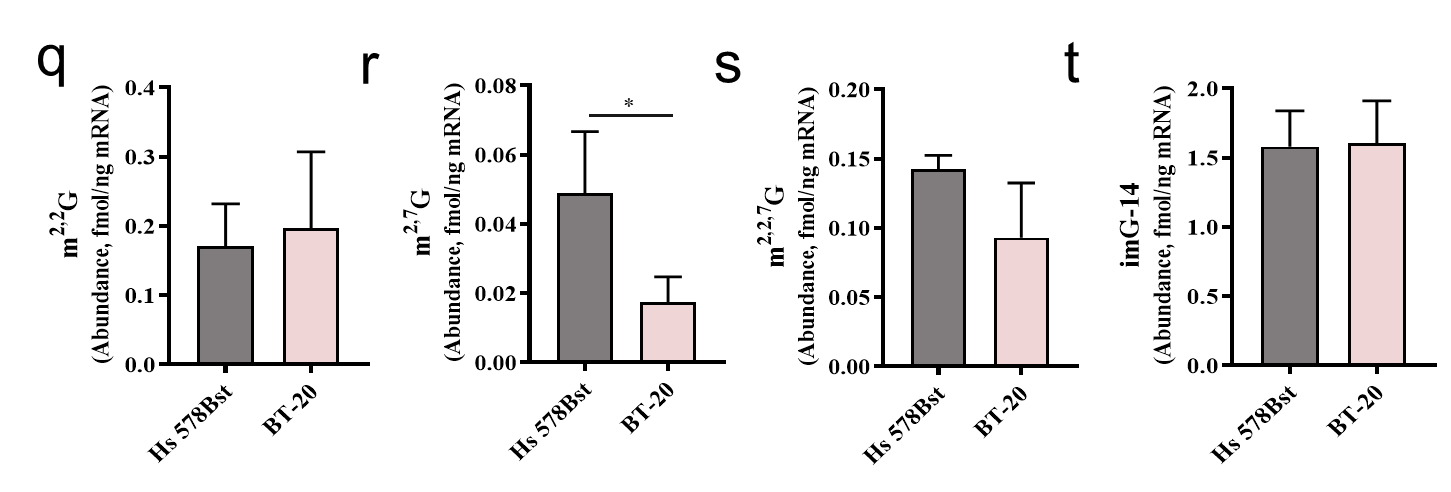

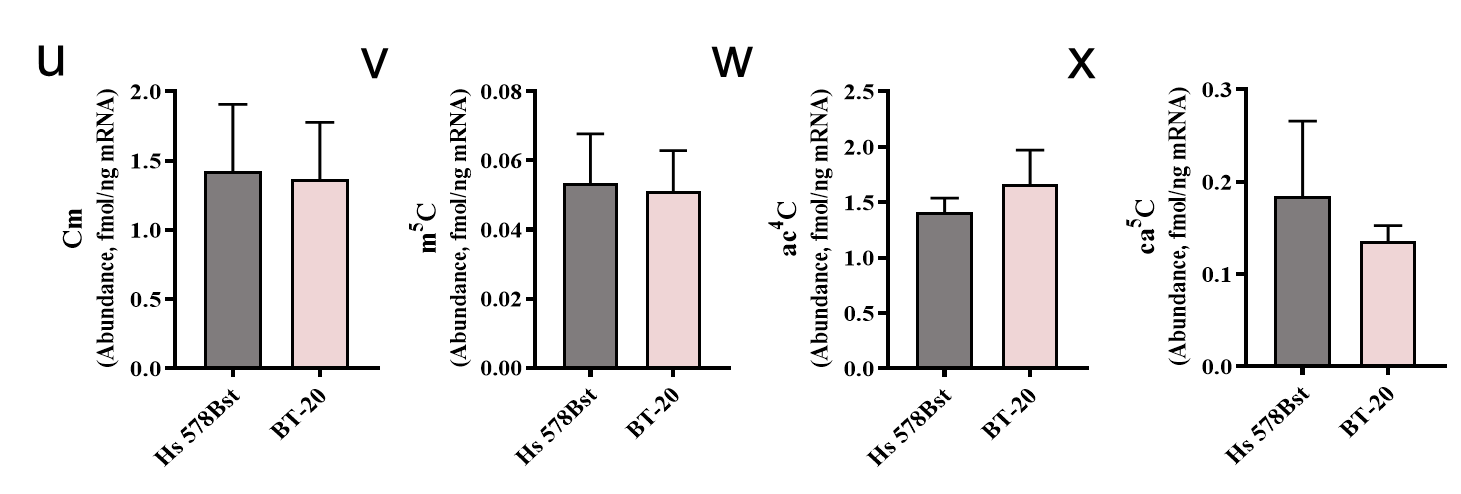

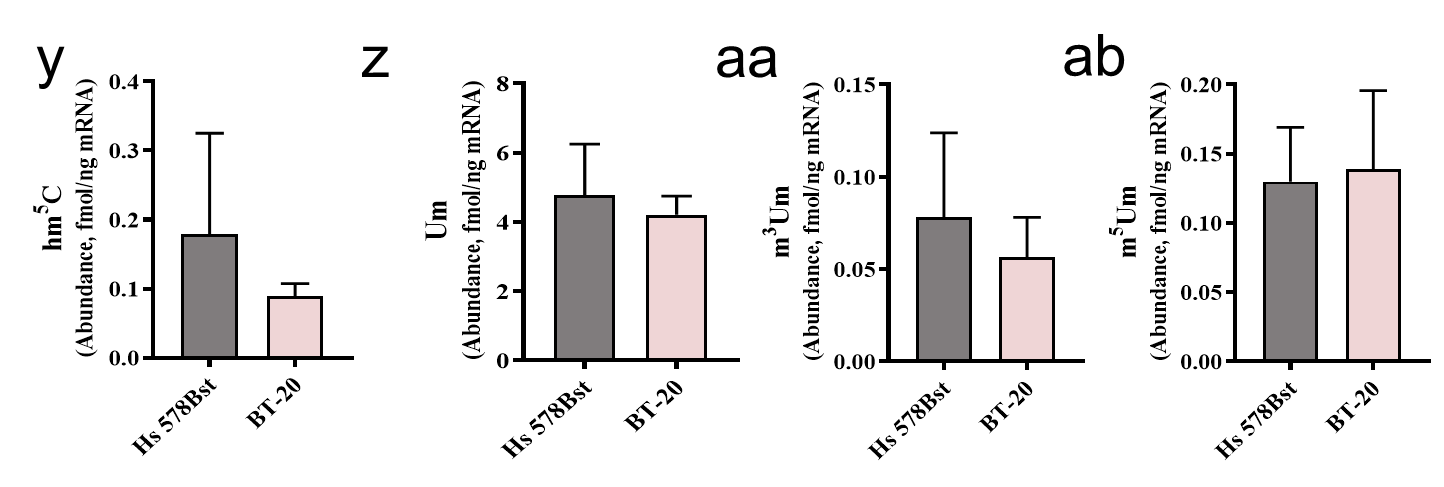

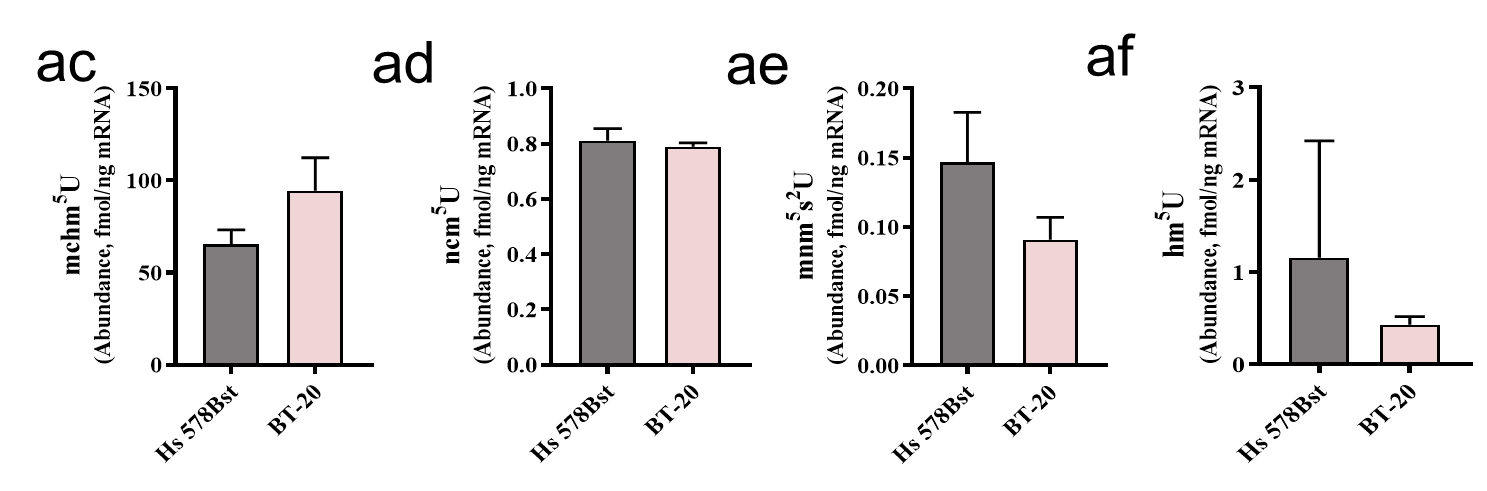

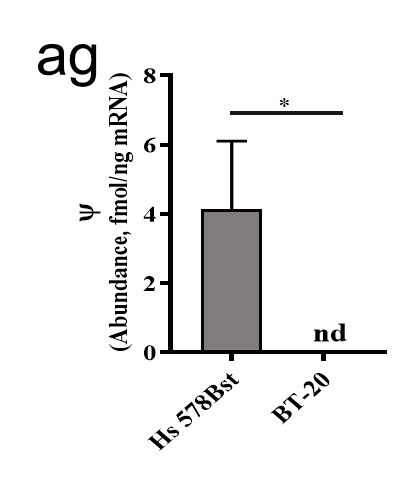


**Figure S7.** Quantification of the release of modified nucleosides during mRNA digestion of HeLa, DDP-treated HeLa, and DDP-resistant HeLa. (a-ab) Am, m^1^A, m^2^A, m^6^A, m^8^A, m^6,6^A, i^6^A, ms^2^m^6^A, t^6^A, I, Gm, m^1^G, m^2^G, m^6^G, m^7^G, m^2,7^G, m^2,2^G, m^2,2,7^G, imG-14, Cm, m^5^C, ac^4^C, ca^5^C, hm^5^C, Um, ncm^5^U, D, ψ. Values represent the mean ± SD of three biological experiments. "nd" indicates not detected. * p < 0.05, ** p < 0.01, *** p < 0.001.


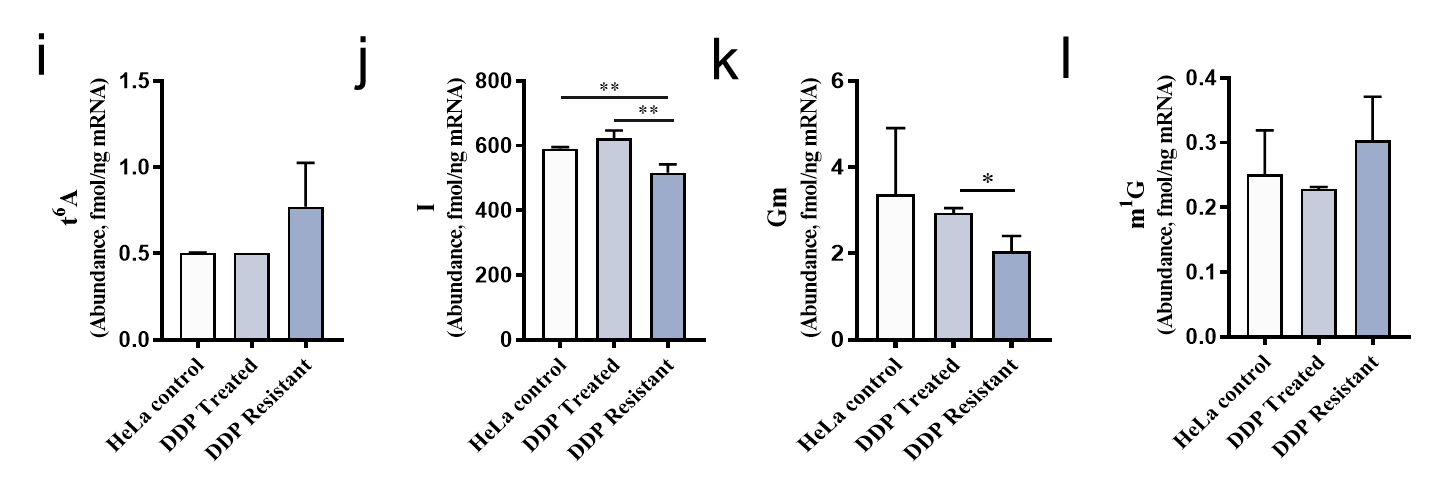

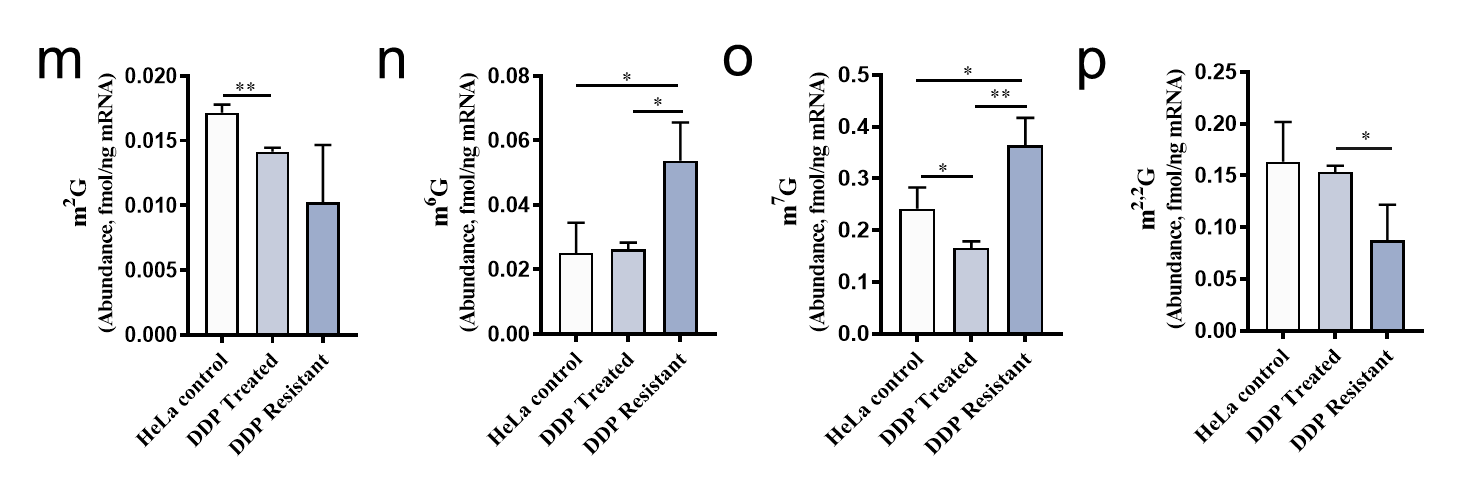

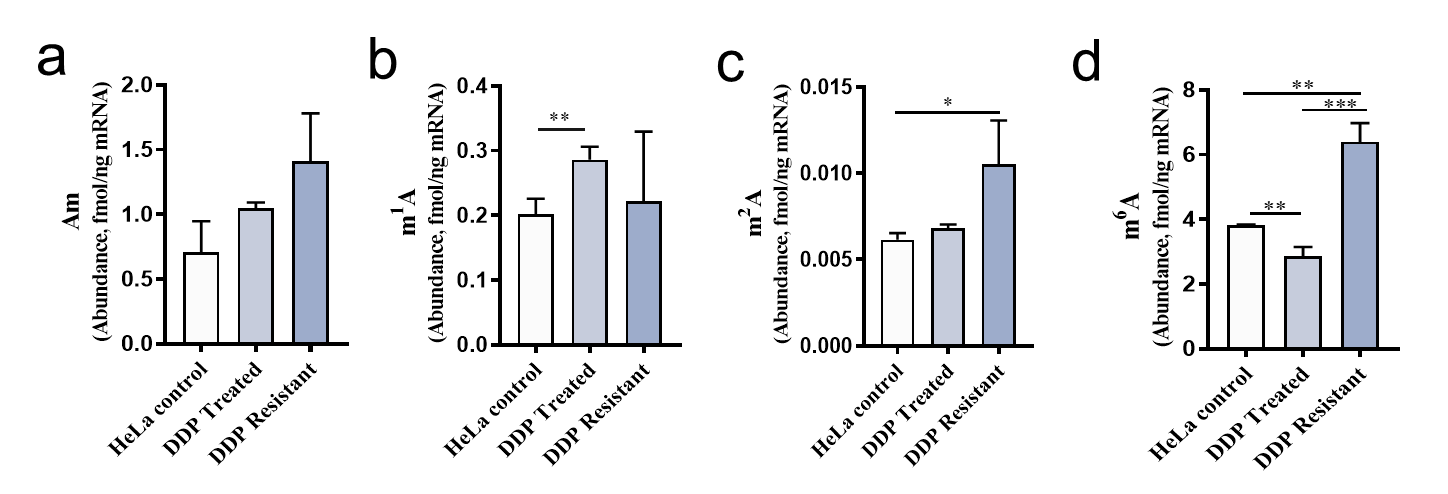

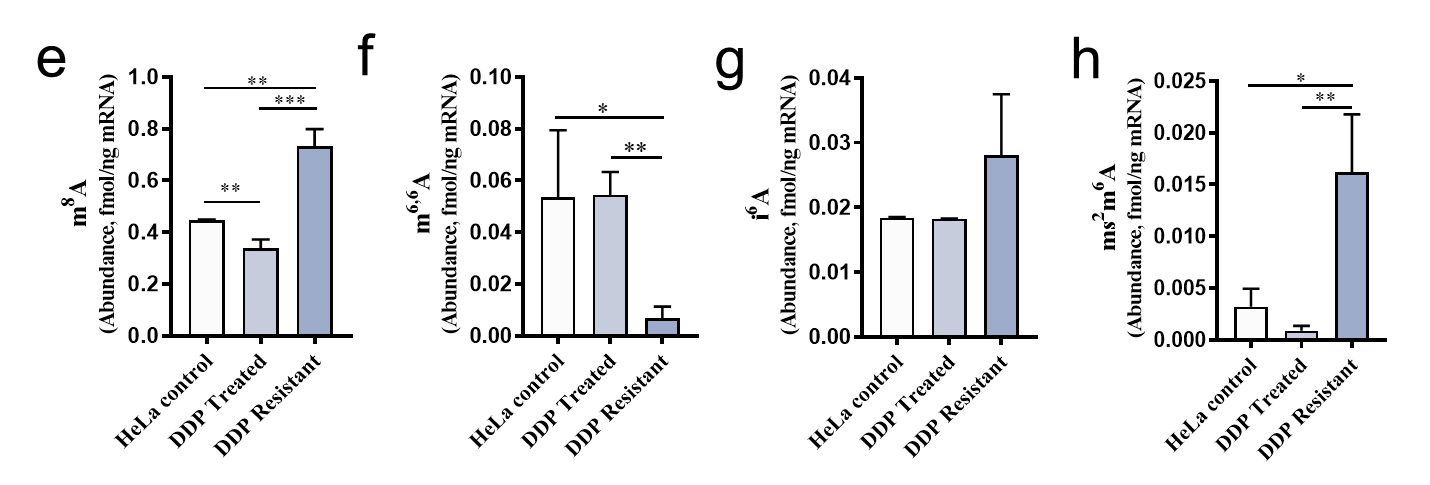


**Figure S7, continued**


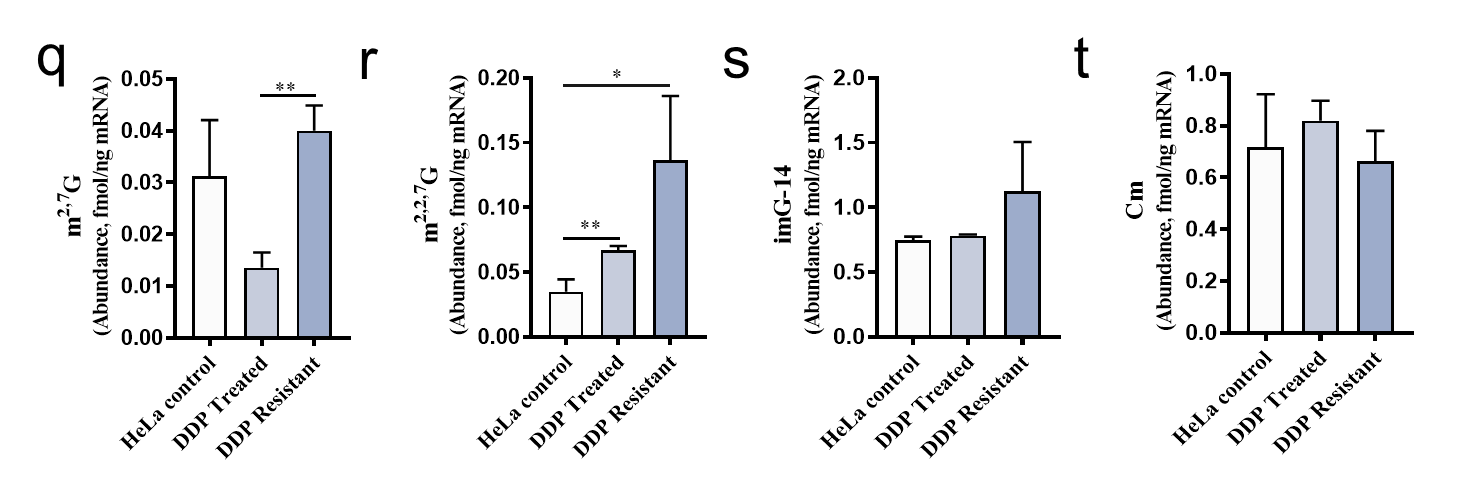

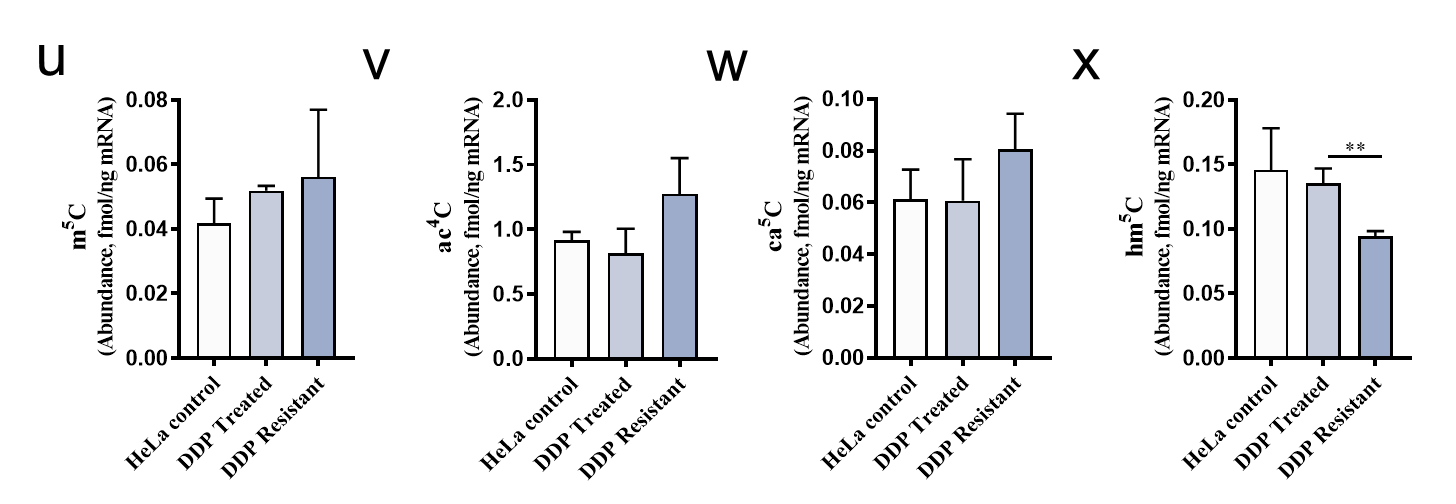

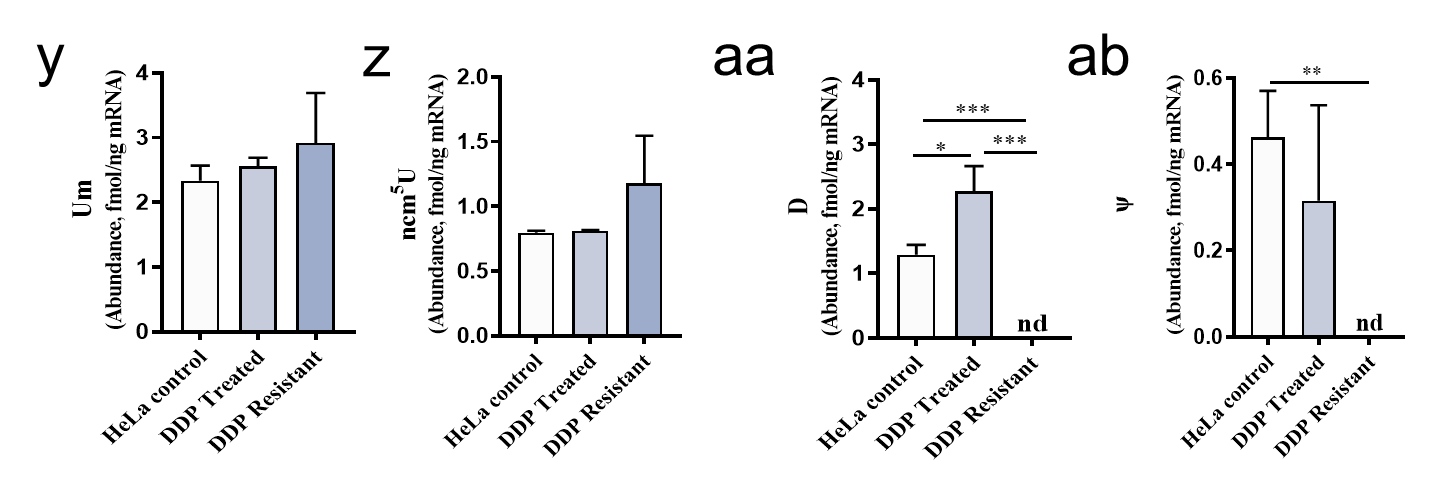


**Figure S8.** Quantification of the release of modified nucleosides during mRNA digestion of HeLa, PTX-treated HeLa, and PTX-resistant HeLa. (a-ab) Am, m^1^A, m^2^A, m^6^A, m^8^A, m^6,6^A, i^6^A, ms^2^m^6^A, t^6^A, I, Gm, m^1^G, m^2^G, m^6^G, m^7^G, m^2,7^G, m^2,2^G, m^2,2,7^G, imG-14, Cm, m^5^C, ac^4^C, ca^5^C, hm^5^C, Um, ncm^5^U, D, ψ. Values represent the mean ± SD of three biological experiments. "nd" indicates not detected. * p < 0.05, ** p < 0.01, *** p < 0.001.


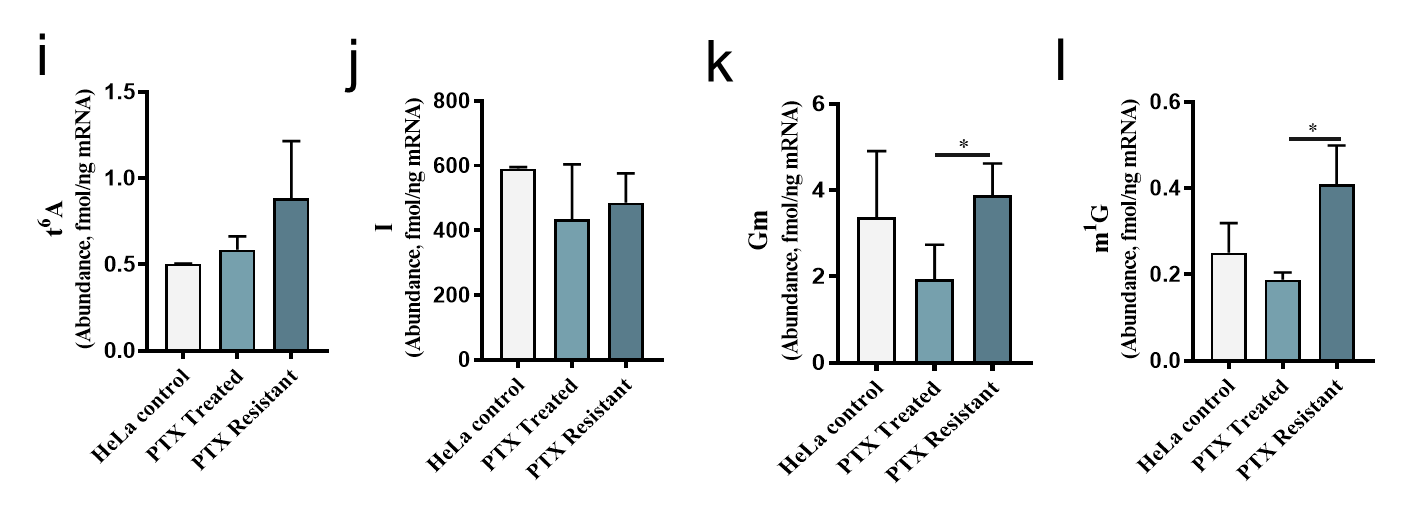

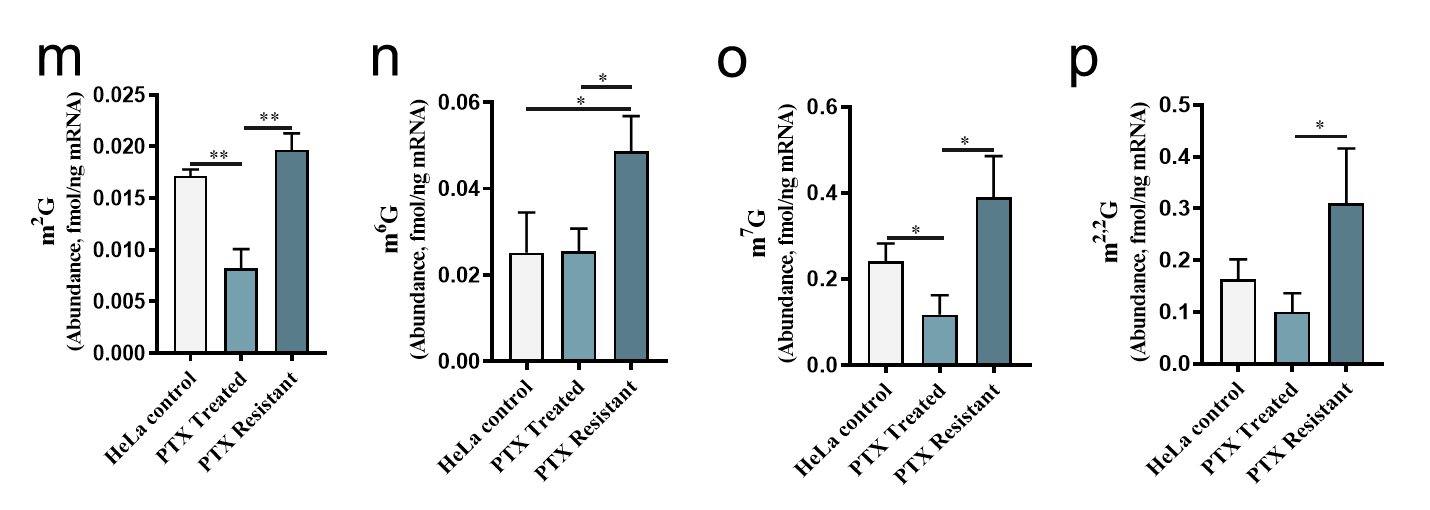

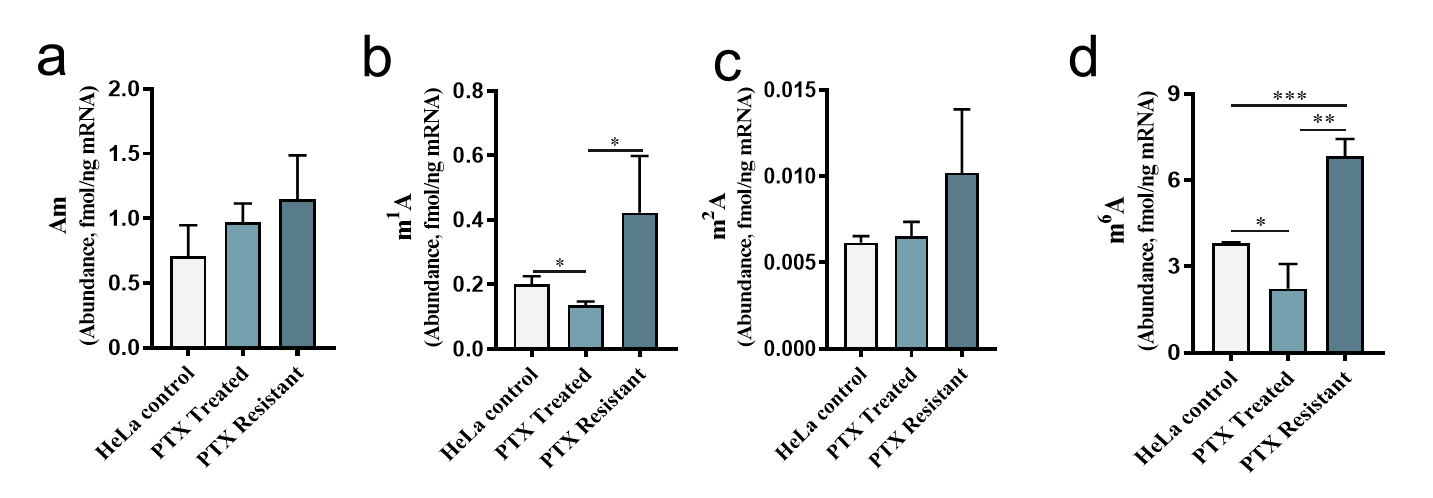

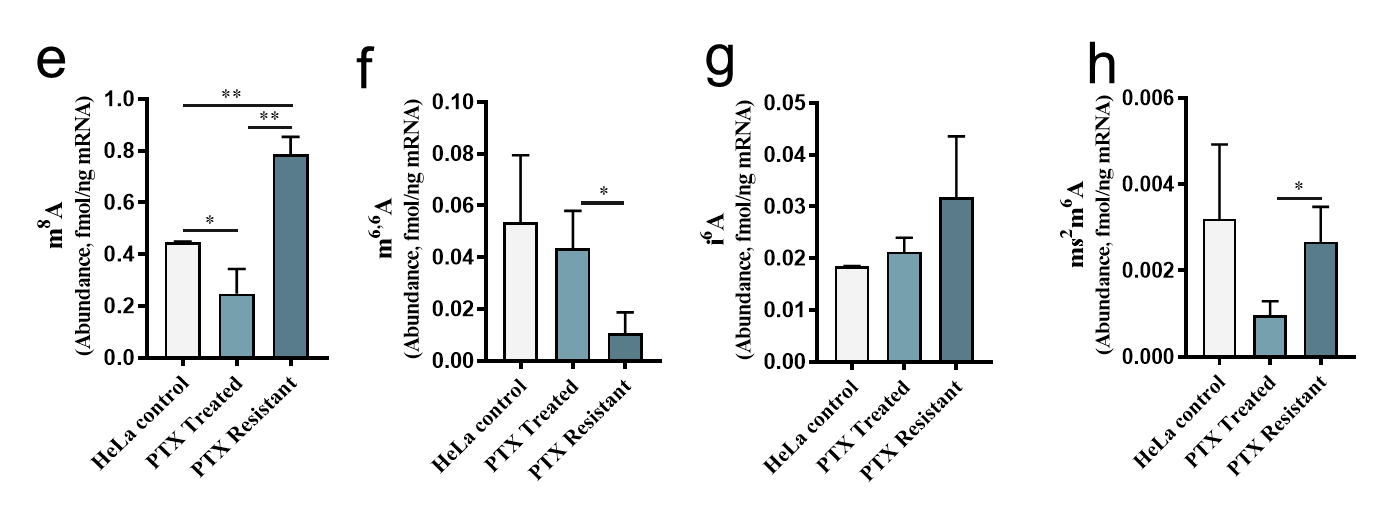


**Figure S8, continued**


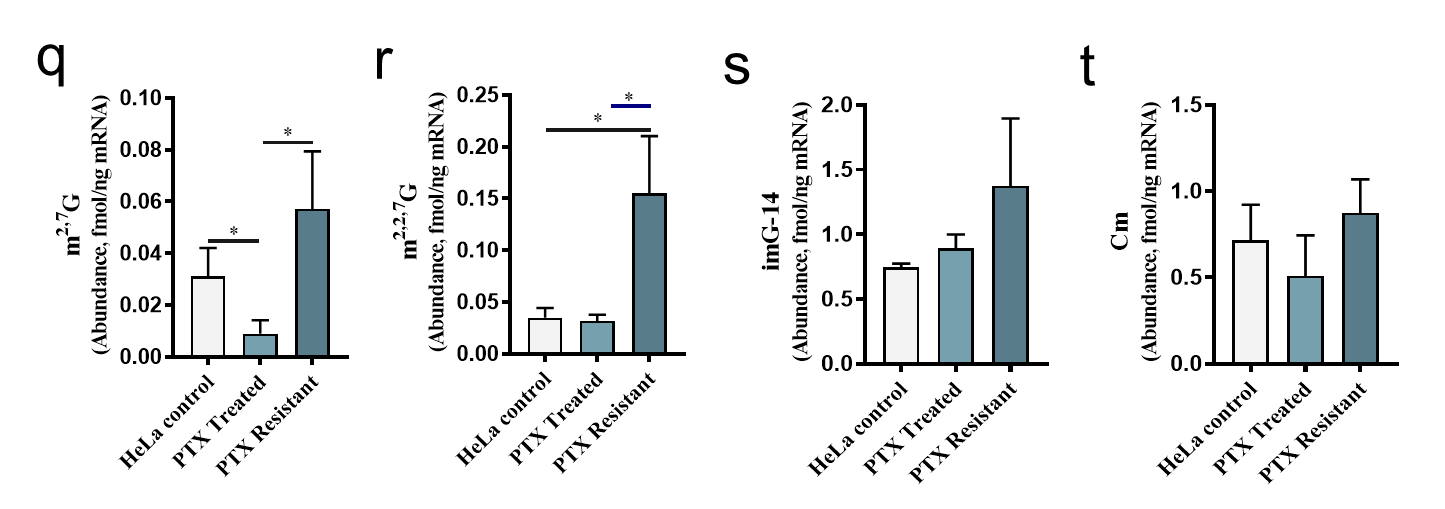

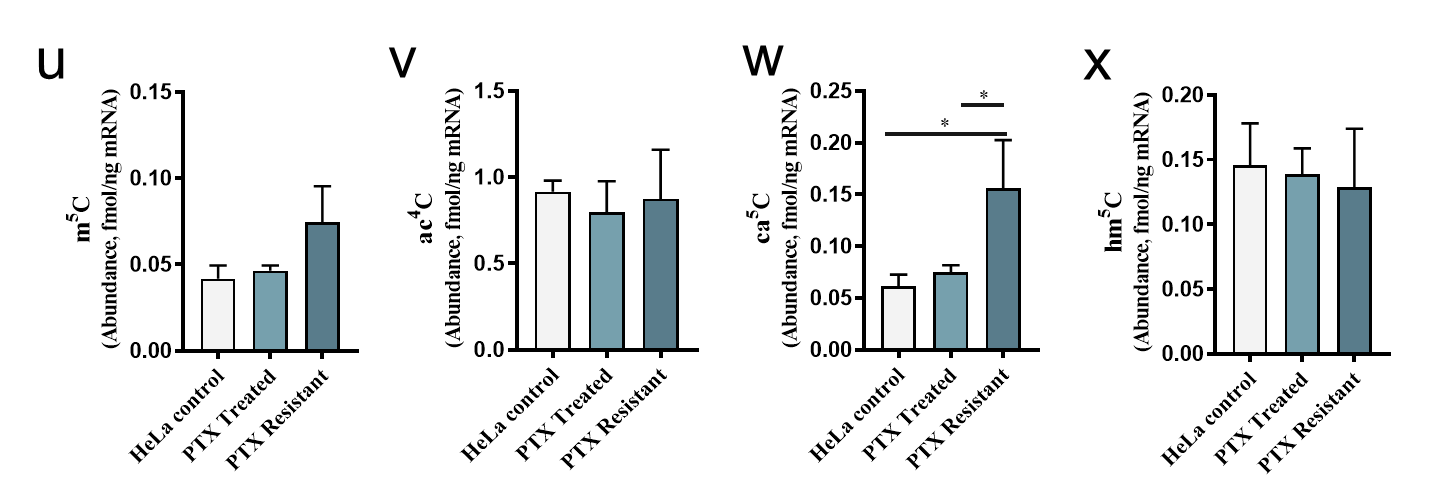

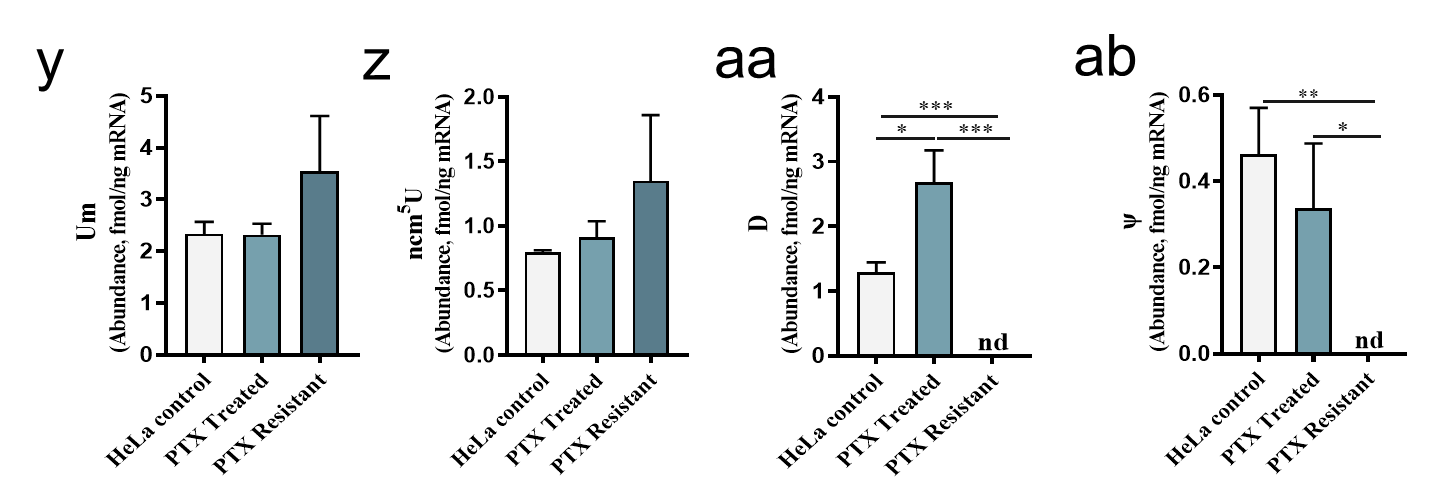


**Figure S9.** Quantification of the release of modified nucleosides during mRNA digestion of HeLa cells after knocking down the m^1^A modification enzymes TRMT6, TRMT61A, TRMT6-61A, TRMT10C, and the demodification enzyme ALKBH3. (a-ab) Am, m^1^A, m^2^A, m^6^A, m^8^A, m^6,6^A, i^6^A, ms^2^m^6^A, t^6^A, I, Gm, m^1^G, m^2^G, m^6^G, m^7^G, m^2,7^G, m^2,2^G, m^2,2,7^G, imG-14, Cm, m^5^C, ac^4^C, ca^5^C, hm^5^C, Um, ncm^5^U, D, ψ. Values represent the mean ± SD of three biological experiments. "nd" indicates not detected. * p < 0.05, ** p < 0.01, *** p < 0.001, **** p < 0.0001.

**Figure S9, continued**


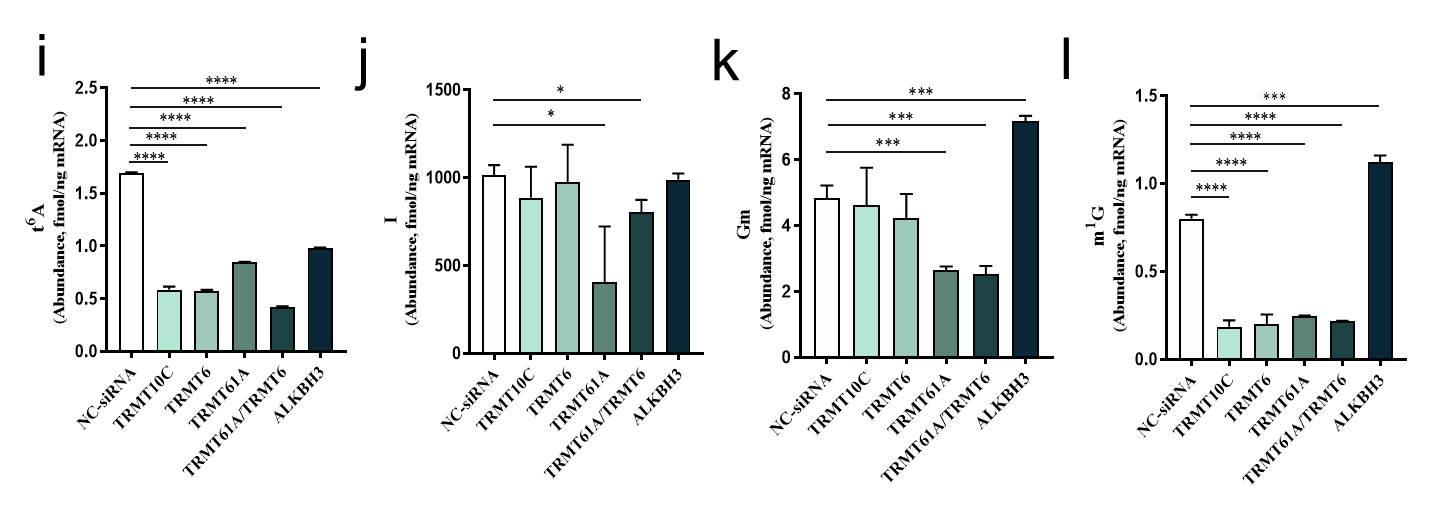

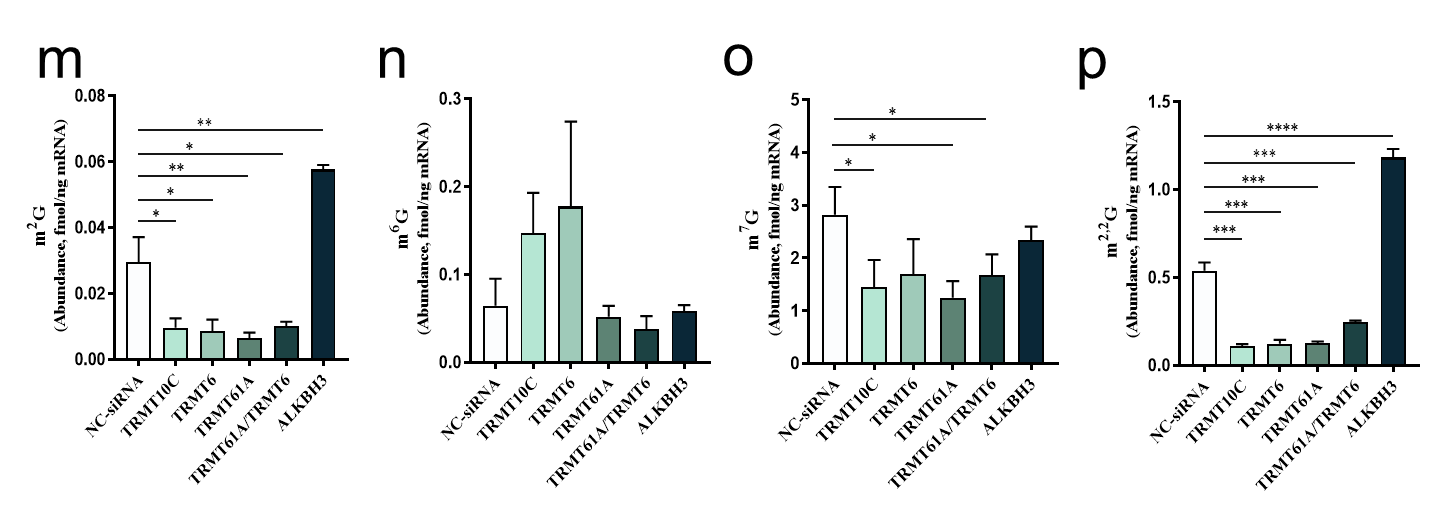

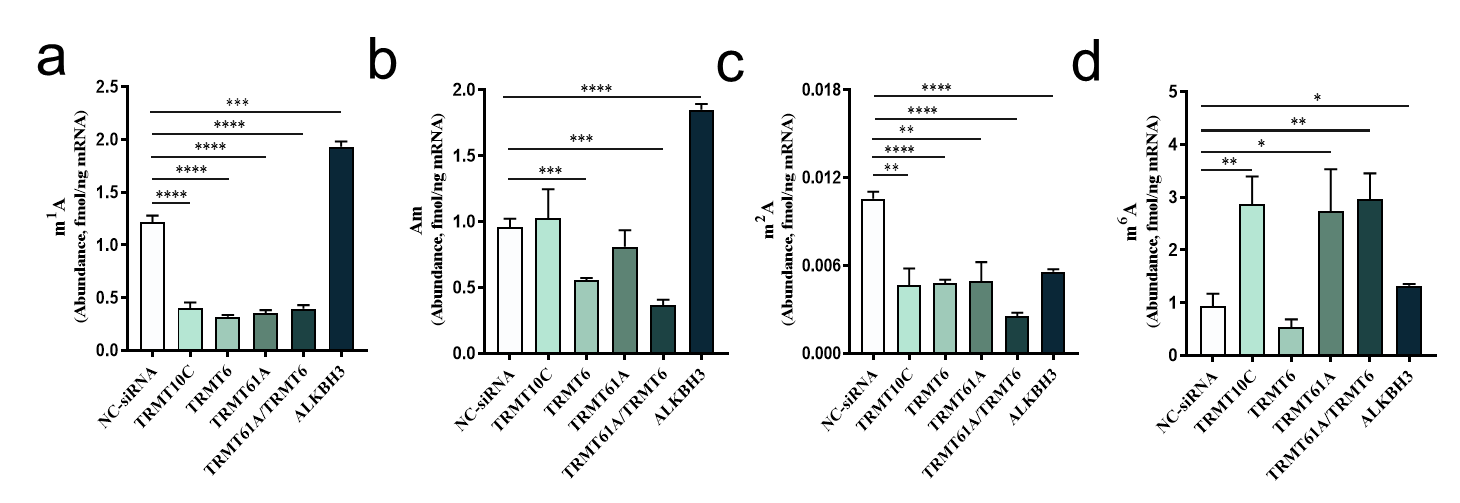

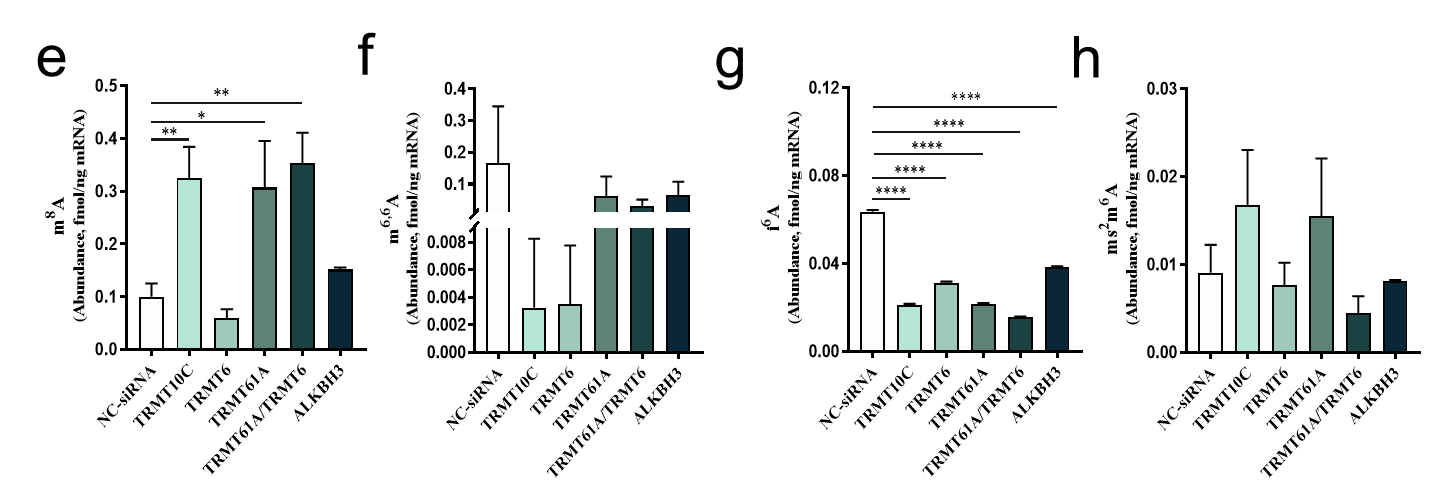

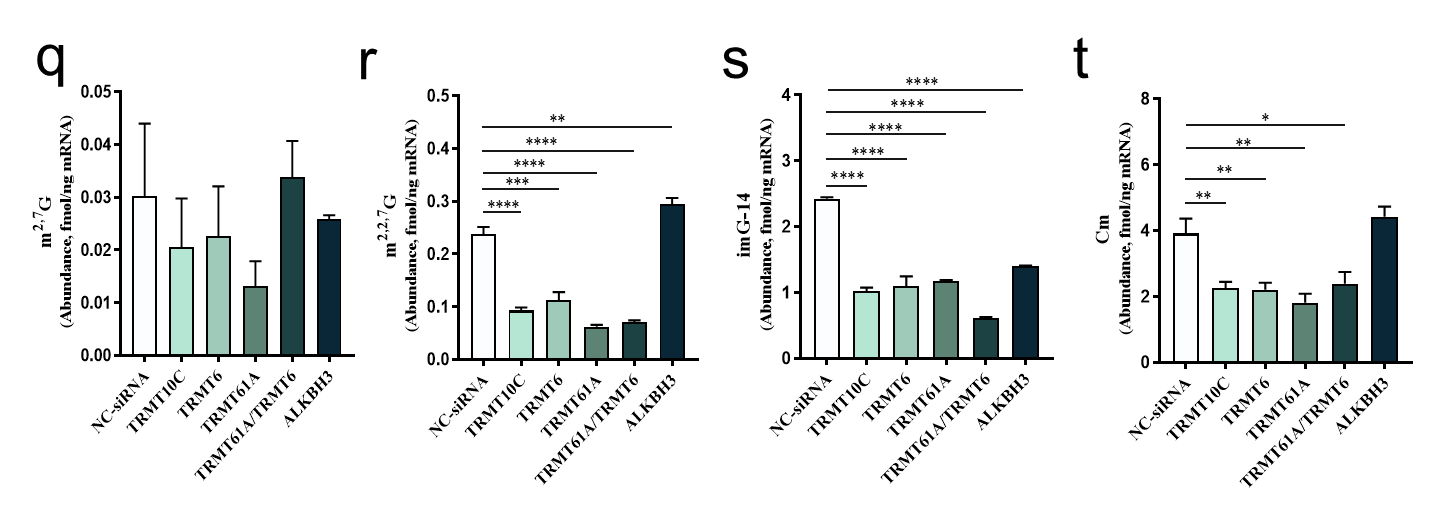

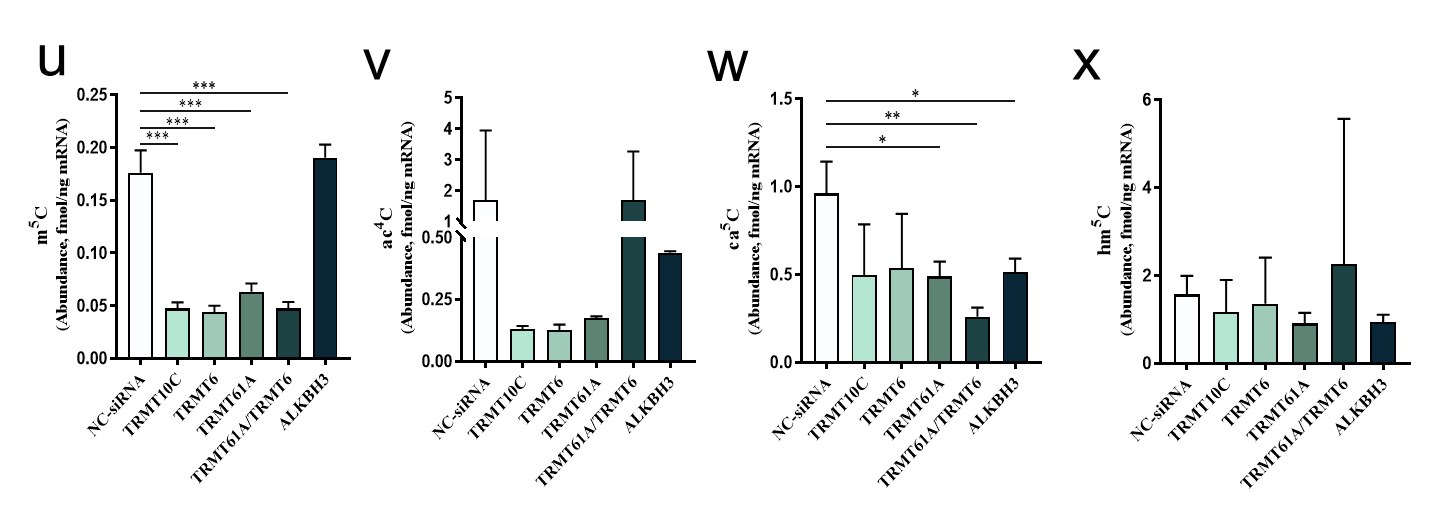

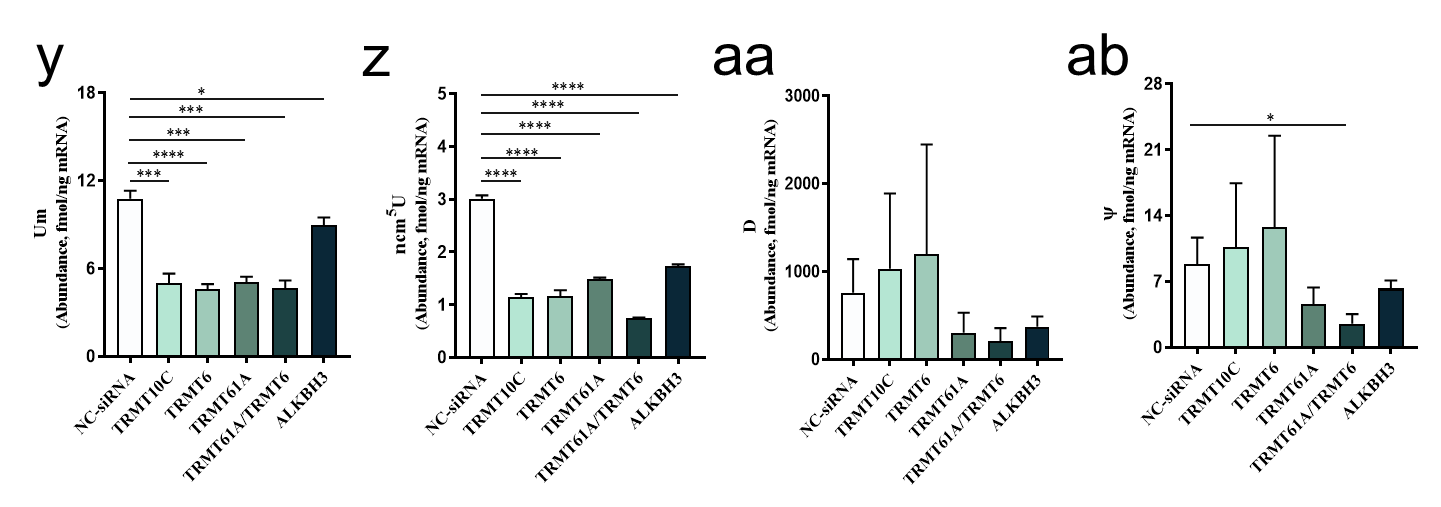


**Figure S10.** Flow cytometry analysis of the impact on the cell cycle after 72h of knockdown of m^1^A regulatory enzymes TRMT10C, ALKBH3, TRMT6, TRMT61A, and TRMT6-61A.


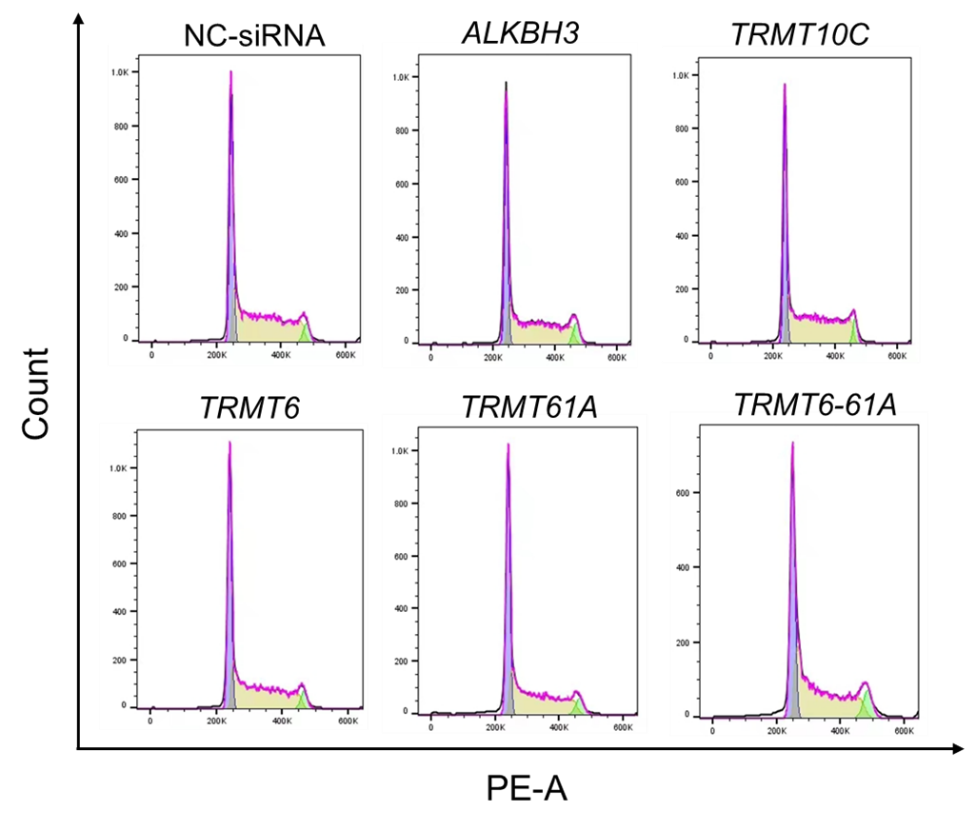


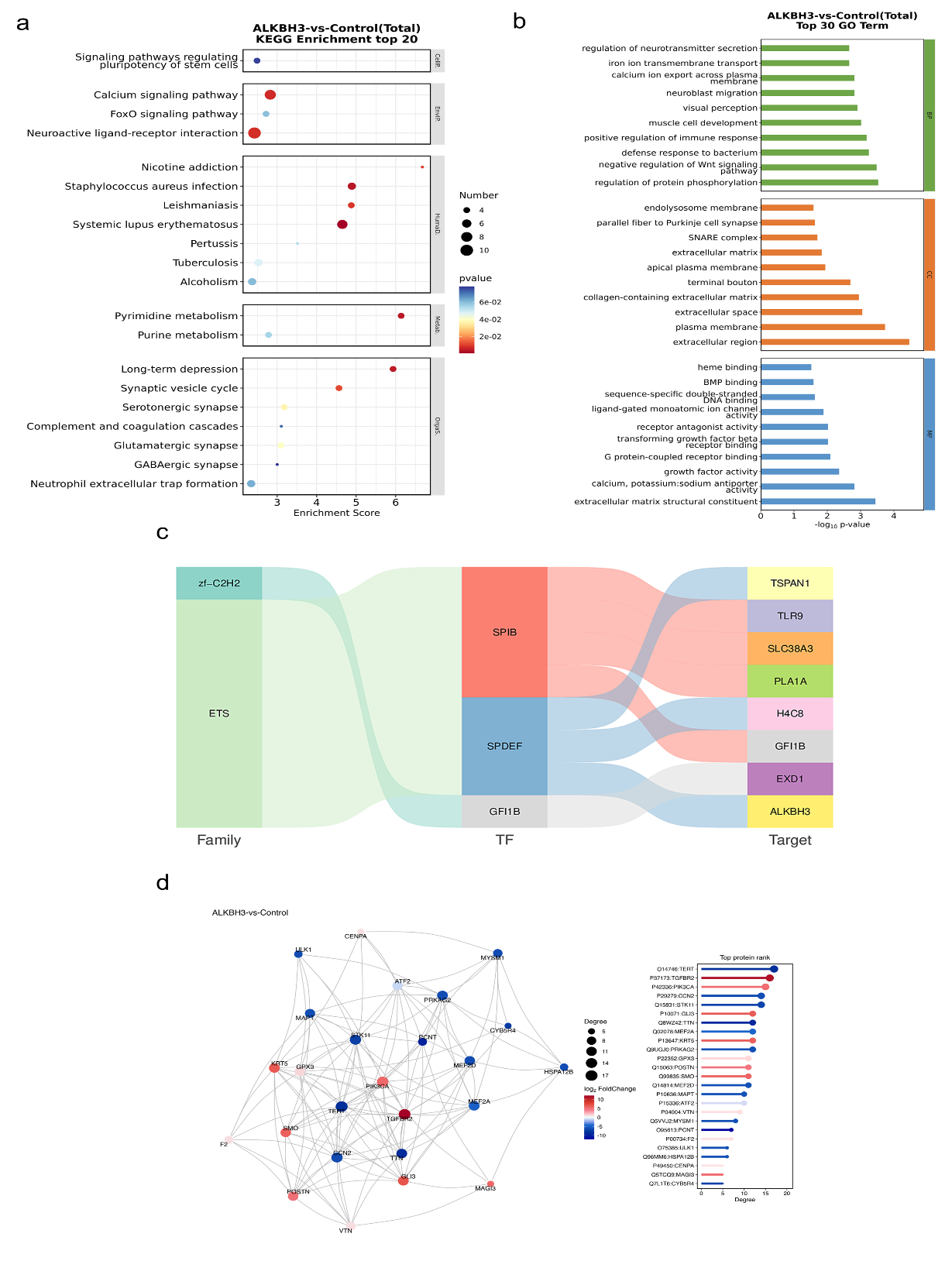
**Figure S11.** Transcriptomic and proteomic analysis of *ALKBH3* treated HeLa cells. Transcriptome: (a) Top 20 KEGG enrichment bubble chart, (b) GO enrichment of the top 30 pathways, (c) Differential transcription factor-target gene Sankey diagram. Proteome: (d) Top 25 connectivity protein interaction network diagram, (e) GO enrichment of the top 15 pathways, (f) Top 20 KEGG enrichment bubble chart. Combined transcriptomic and proteomic analysis: (g) Bar chart of the top 30 KEGG (GSEA) pathways shared across different omics.


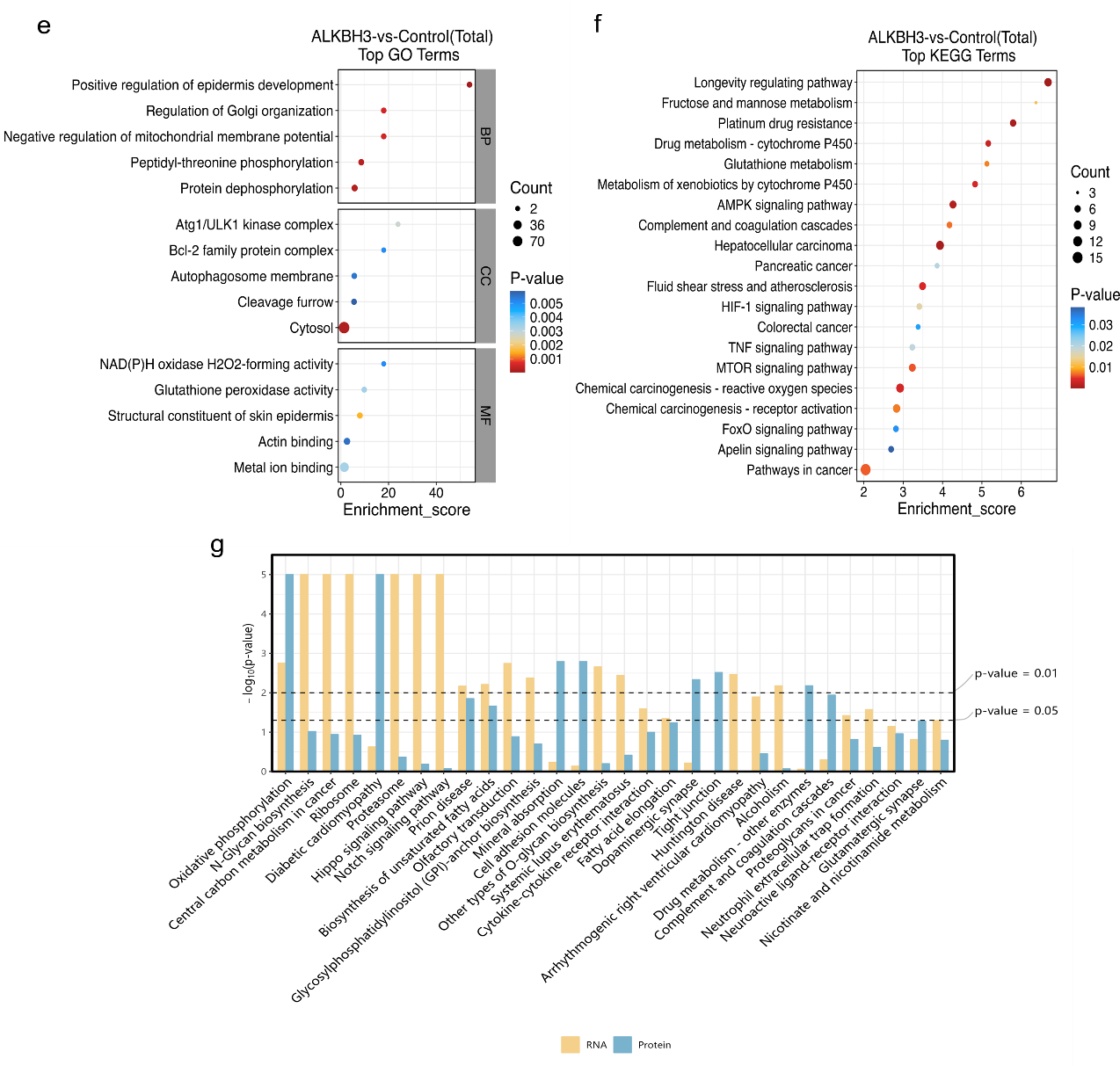
**Figure S11, continued**


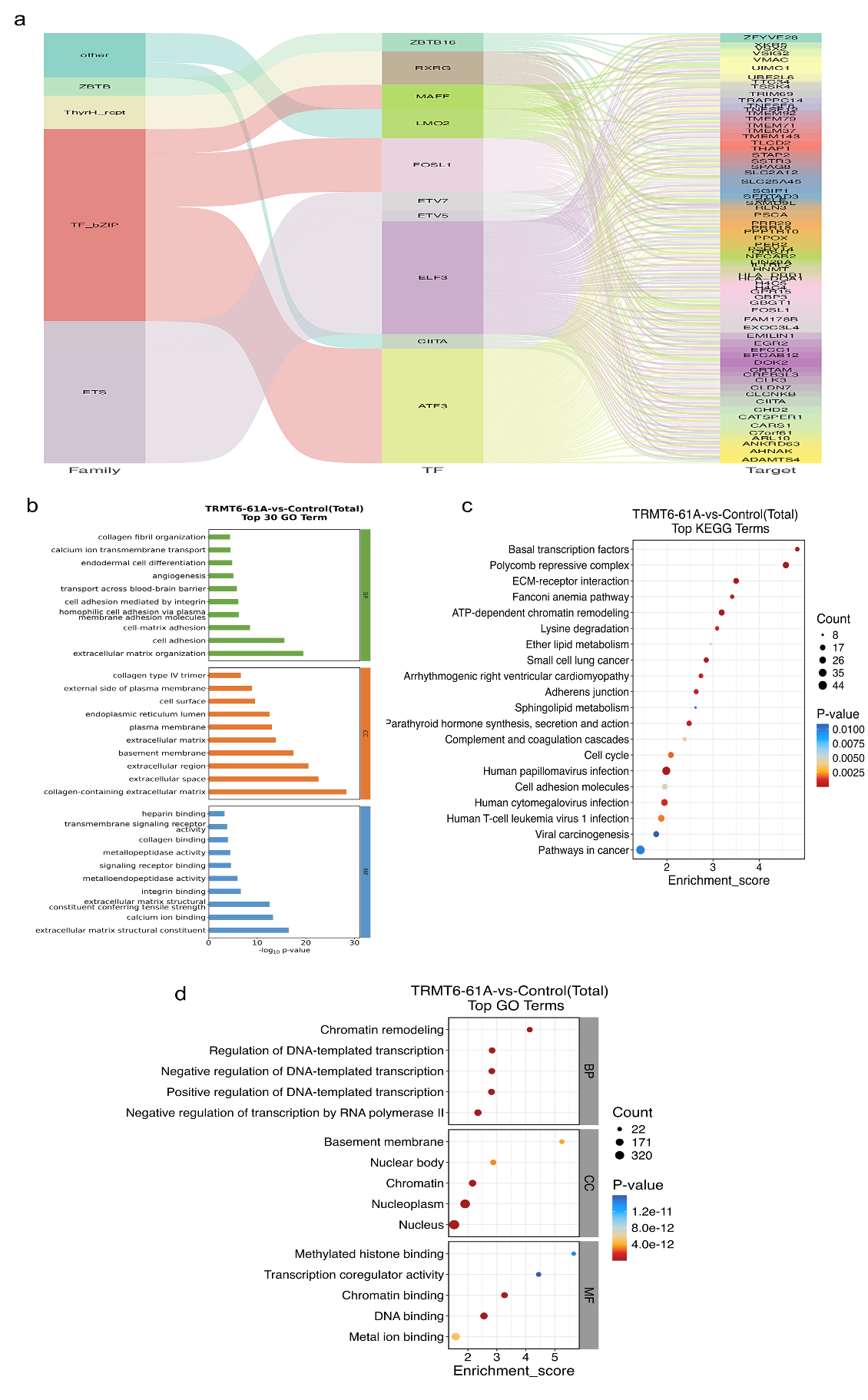
**Figure S12.** Transcriptomic and proteomic analysis of *TRMT6-61A* treated HeLa cells. Transcriptome: (a) Differential transcription factor-target gene Sankey diagram, (b) GO enrichment of the top 30 pathways. Proteome: (c) Top 20 KEGG enrichment bubble chart, (d) GO enrichment of the top 15 pathways.

**Figure S13.** Transcriptomic and proteomic analysis of *TRMT6* treated HeLa cells. Transcriptome: (a) Top 20 KEGG enrichment bubble chart, (b) GO enrichment of the top 30 pathways, (c) Differential transcription factor-target gene Sankey diagram. Proteome: (d) Top 25 connectivity protein interaction network diagram, (e) GO enrichment of the top 15 pathways, (f) Top 20 KEGG enrichment bubble chart. Combined transcriptomic and proteomic analysis: (g) Bar chart of the top 30 KEGG
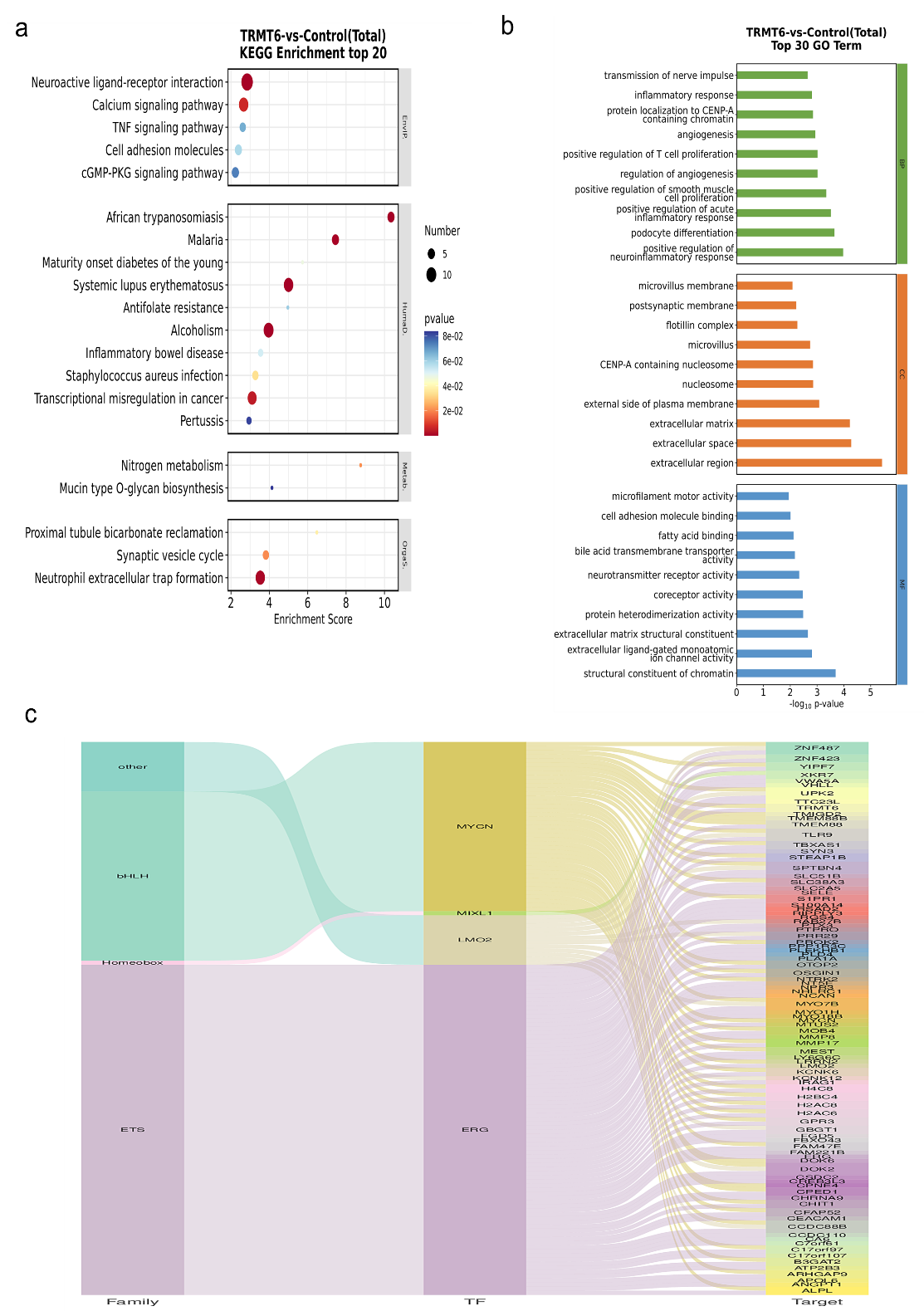
(GSEA) pathways shared across different omics.


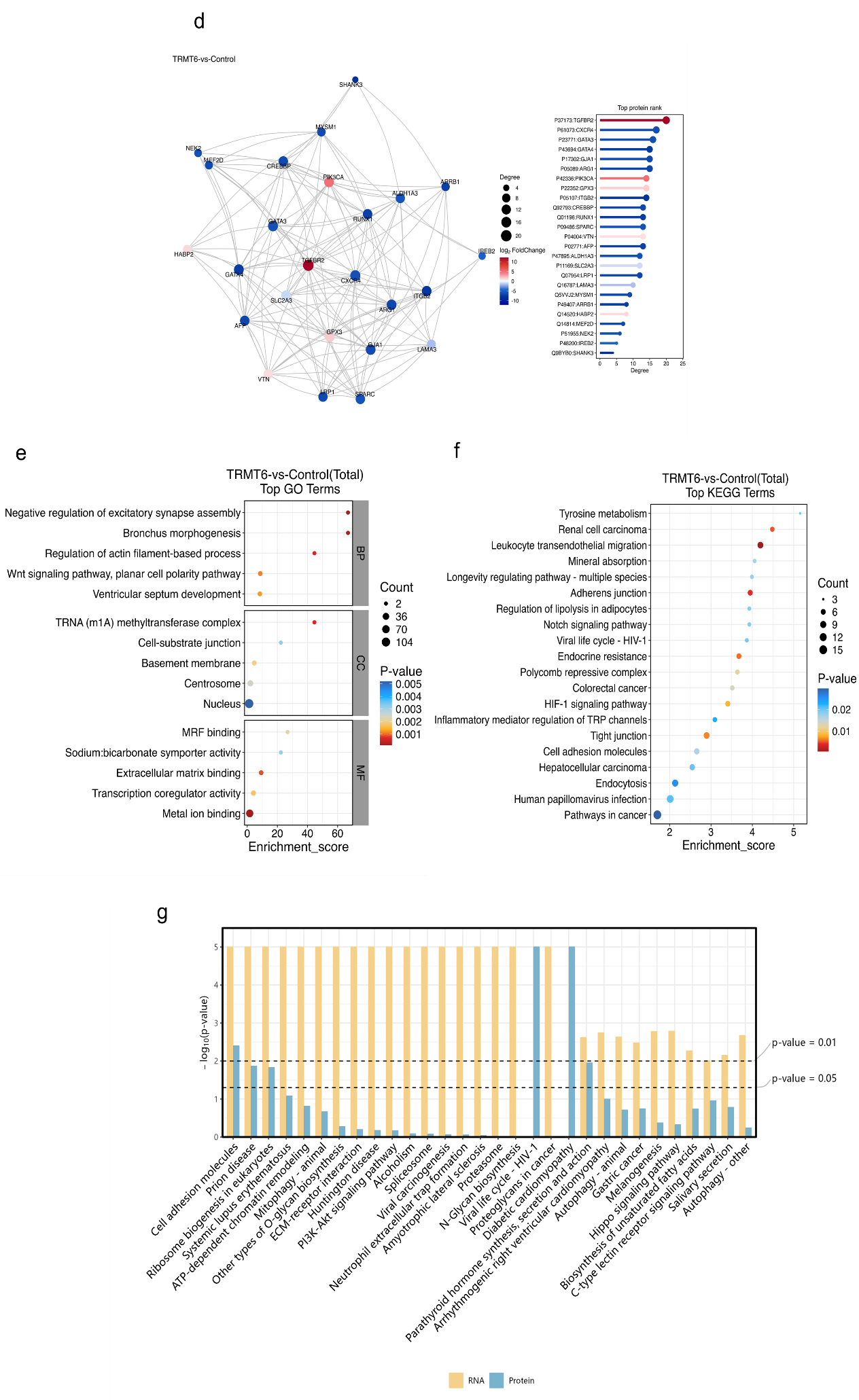
**Figure S13, continued**

**Figure S14.** Transcriptomic and proteomic analysis of *TRMT61A* treated HeLa cells. Transcriptome: (a) Top 20 KEGG enrichment bubble chart, (b) GO enrichment of the top 30 pathways, (c) Differential transcription factor-target gene Sankey diagram. Proteome: (d) Top 25 connectivity protein interaction network diagram, (e) GO enrichment of the top 15 pathways, (f) Top 20 KEGG enrichment bubble chart. Combined transcriptomic and proteomic analysis: (g) Bar chart of the top 30 KEGG (GSEA) pathways shared across different omics.


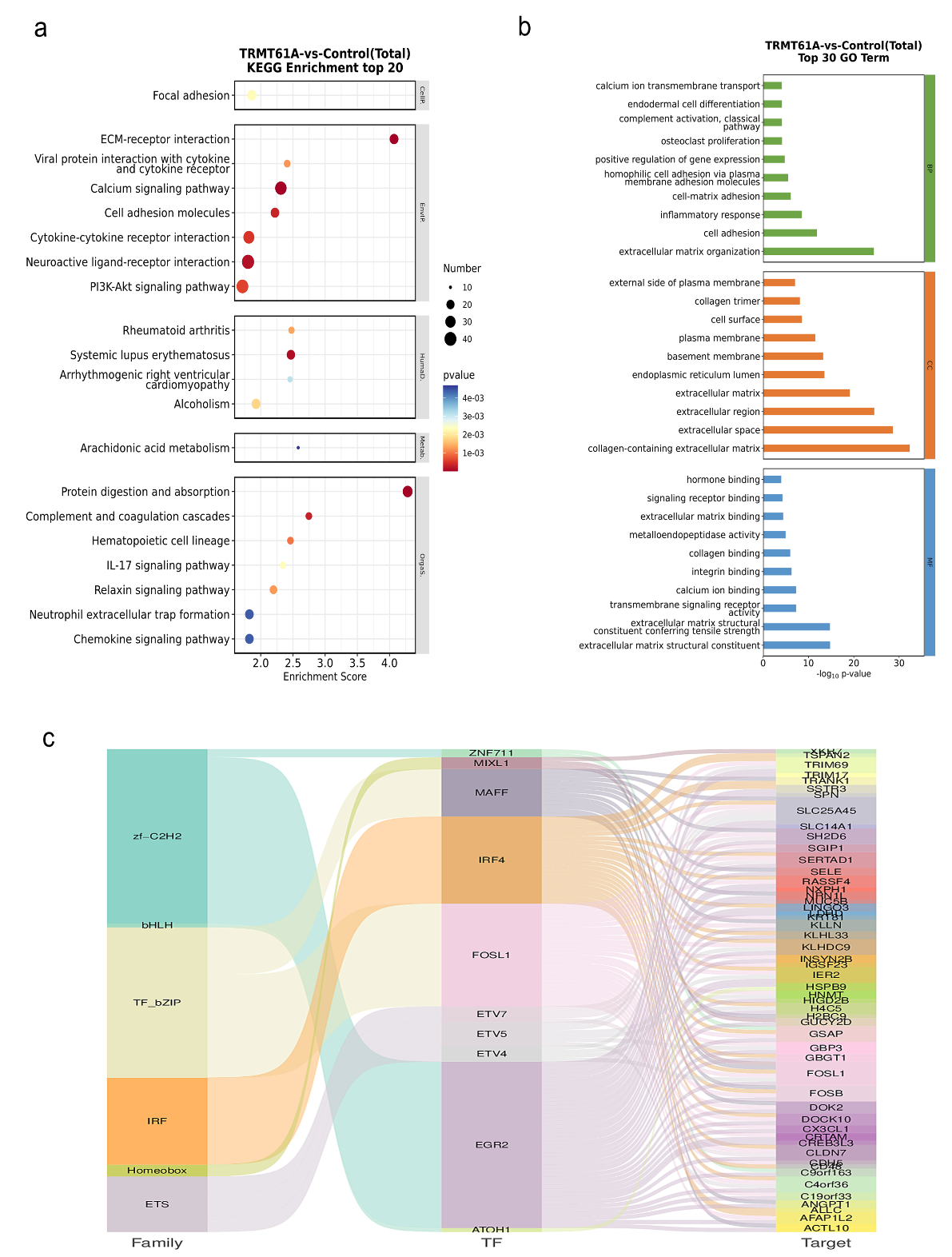
**
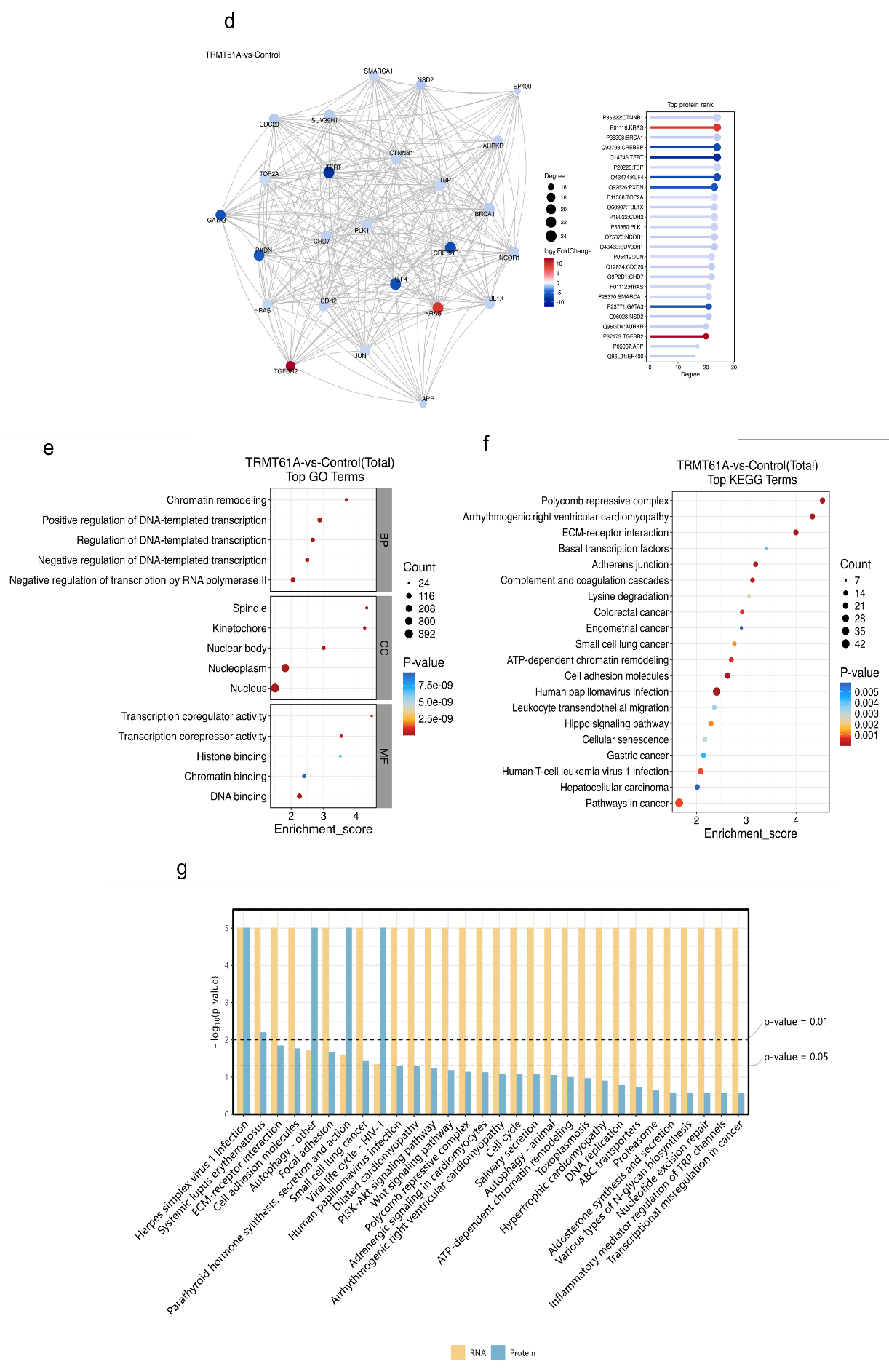
****Figure S14, continued**

**Figure S15.** Transcriptomic and proteomic analysis of *TRMT10C* treated HeLa cells. Transcriptome: (a) Top 20 KEGG enrichment bubble chart, (b) GO enrichment of the top 30 pathways, (c) Differential transcription factor-target gene Sankey diagram. Proteome: (d) Top 25 connectivity protein interaction network diagram, (e) GO enrichment of the top 15 pathways, (f) Top 20 KEGG enrichment bubble chart. Combined transcriptomic and proteomic analysis: (g) Bar chart of the top 30 KEGG
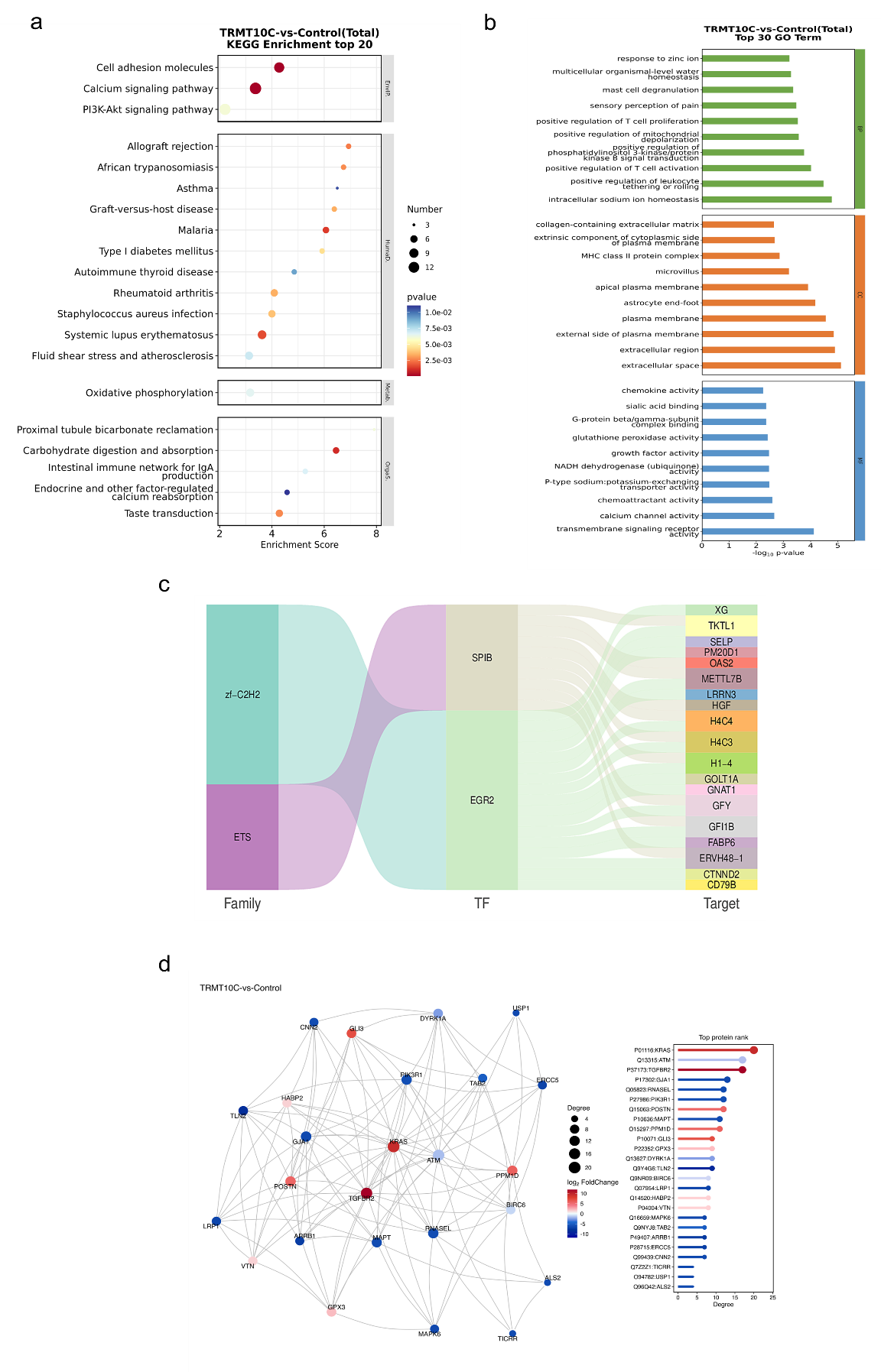
(GSEA) pathways shared across different omics.

**
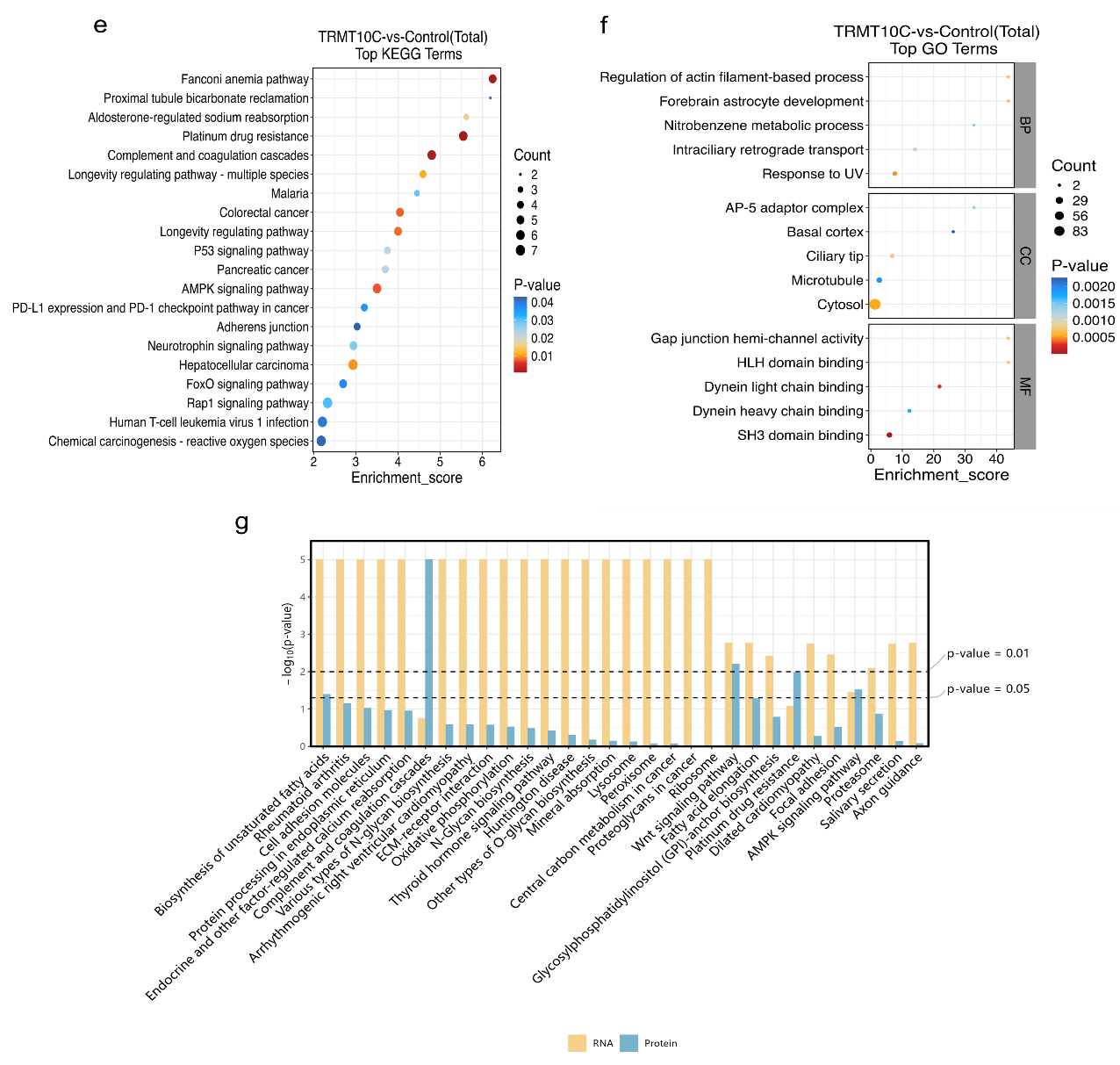
Figure S15, continued**

**Figure S16.** Quantitative analysis using single-standard spike-in of m^3^C, m^4^C, m^5^C, and m^6^C (exemplified in HeLa cells). (a) m^3^C, (b) m^4^C, (c) m^5^C, (d) m^6^C.


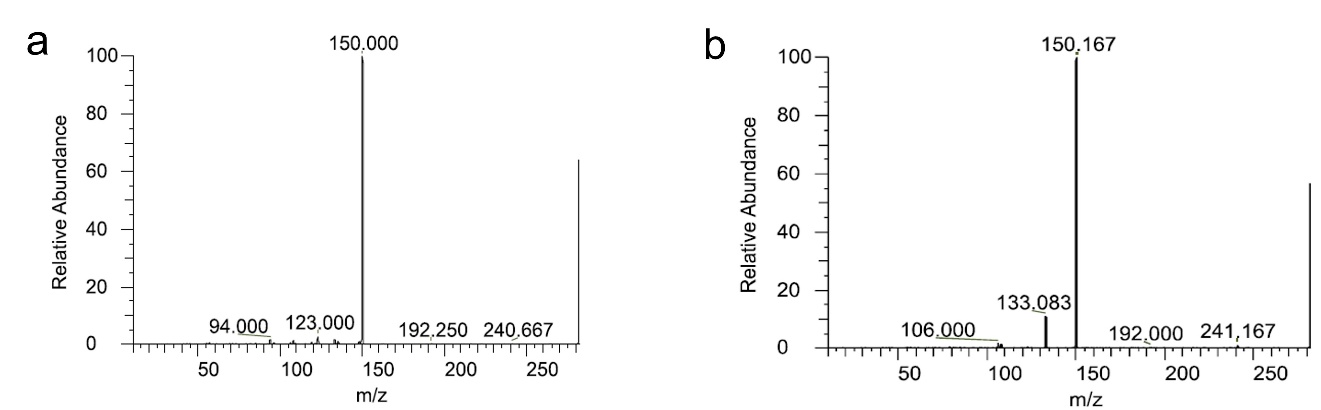
**Figure S17.** Product Spectrum of isobaric nucleosides: (a) m^6^A, (b) m^8^A


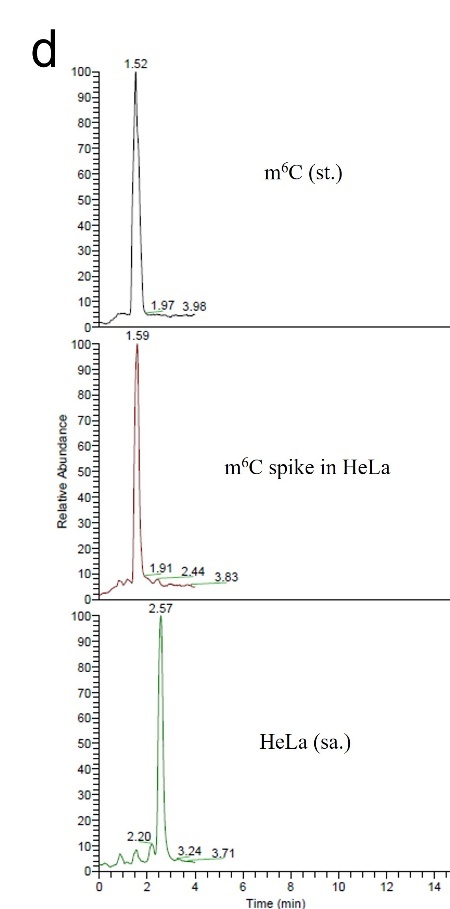

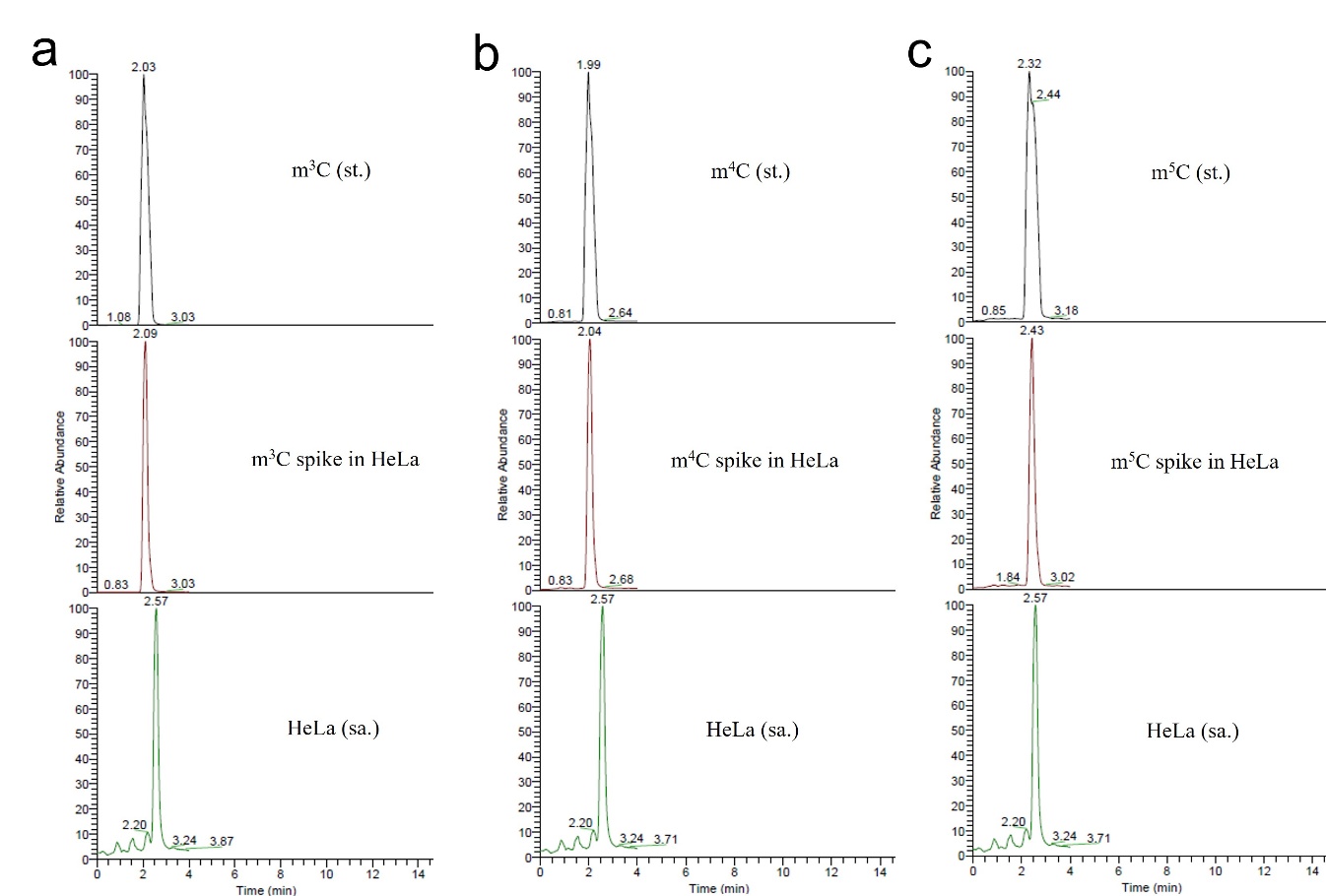


**Table S1.** siRNA used for transfection. S stands for Sense, and A stands for Antisense.

| ***NC-siRNA*** | |
| --- | --- |
| S | UUGUACUACACAAAAGUACUG |
| A | GUACUUUUGUGUAGUACAAUU |
| ***TRMT6-siRNA (NM_015939)*** | |
| S | GGGAAAUCUCAGAAACUUATT |
| A | UAAGUUUCUGAGAUUUCCCTT |
| ***TRMT61A-siRNA (NM_152307)*** | |
| S | GACAGUGCCUAUGUGAUCATT |
| A | UGAUCACAUAGGCACUGUCTT |
| ***TRMT10C-siRNA (NM_017819)*** | |
| S | GAGUGUUAGUGUCAAUUUCTT |
| A | GAAAUUGACACUAACACUCTT |
| ***ALKBH3-siRNA (NM_139178)*** | |
| S | UGGCUUUGUUGACGUGAAATT |
| A | UUUCACGUCAACAAAGCCATT |

**Table S2.** Primers used for qPCR analysis.

| **Gapdh Human qPCR Primer Pair (NM_002046)** | | |
| --- | --- | --- |
| F | TGACATCAAGAAGGTGGTGAAGCAGG | product length = 100 |
| R | GCGTCAAAGGTGGAGGAGTGGGT |  |
| **TRMT6 Human qPCR Primer Pair (NM_015939)** | | |
| F | CTGTCTTTGCTGGACTTTGTGGC | product length = 104 |
| R | AGACAGCCTGAGGTTGATGACC |  |
| **TRMT61A Human qPCR Primer Pair (NM_152307)** | | |
| F | TCCTCTACTCCACAGACATCGC | product length = 98 |
| R | CAATGGTGCGGATGATGGCGTG |  |
| **TRMT10C Human qPCR Primer Pair (NM_017819)** | | |
| F | CAAAACGGCTGAACCTGGCAAC | product length = 122 |
| R | GCAGAGCCTCTTGCCAATTACC |  |
| **ALKBH3 Human qPCR Primer (NM_139178)** | | |
| F | CCACTGCTAAGAGCCATCTCCA | product length = 114 |
| R | TCAATCACTCGTGGCTCAGGAG |  |

**Table S3**. Cellular composition at different cell cycle phases after knocking down m^1^A regulatory enzymes in HeLa cells.

| groups | G1% | S% | G2/M% |
| --- | --- | --- | --- |
| *NC-siRNA* | 32.9±2.0 | 56.4±2.5 | 4.2±0.5 |
| *ALKBH3* | 37.0±0.6 | 51.0±0.6 | 6.0±0.8 |
| *TRMT10C* | 32.9±0.3 | 56.1±0.5 | 4.9±0.8 |
| *TRMT6* | 38.9±0.4 | 50.6±0.3 | 4.5±0.5 |
| *TRMT61A* | 38.6±1.1 | 47.4±1.3 | 5.5±1.3 |
| *TRMT6+TRMT61A* | 35.9±1.5 | 47.5±2.1 | 8.1±0.8 |

**Table S4.** Detection limits of known and potentially existing modified nucleosides studied.

| Compound | Limit of quantification (LOQ), amol | Limit of detection (LOD), amol |
| --- | --- | --- |
| hm^5^C | 8.07 | 2.42 |
| ψ | 2310 | 694 |
| ca^5^C | 77.2 | 23.2 |
| s^2^ψ | 22200 | 6670 |
| ncm^5^U | 179 | 53.7 |
| hm^5^U | 1850 | 412 |
| m^1^A | 0.391 | 0.117 |
| m^2^A | 34.3 | 10.3 |
| m^6^A | 5.29 | 1.59 |
| m^8^A | 11.5 | 3.46 |
| D | 2650 | 794 |
| mnm^5^s^2^U | 229 | 68.6 |
| m^3^C | 27.4 | 8.23 |
| m^4^C | 9.80 | 2.94 |
| m^5^C | 41.2 | 12.4 |
| m^6^C | 6.24 | 1.87 |
| s^2^C | 123 | 37.0 |
| cm^5^U | 187 | 56.1 |
| Cm | 95.0 | 28.5 |
| m^1^ψ | 18500 | 5560 |
| m^3^ψ | 2650 | 794 |
| ho^5^U | 726 | 218 |
| m^5^D | 125 | 37.4 |
| s^2^U | 2180 | 654 |
| s^4^U | 8550 | 2560 |
| Um | 19600 | 5880 |
| c^7^A | 10.6 | 3.17 |
| m^1^G | 309 | 92.6 |
| m^2^G | 12.5 | 3.74 |
| m^6^G | 337 | 101 |
| m^7^G | 31.7 | 9.50 |
| f^5^C | 247 | 74.1 |
| I | 114 | 34.3 |
| m^1^Am | 4.57 | 1.37 |
| m^6^Am | 22.9 | 6.86 |
| m^3^U | 412 | 123 |
| m^5^U | 11.7 | 3.52 |
| mo^5^U | 882 | 265 |
| m^5^s^2^U | 374 | 112 |
| c^7^G | 7940 | 2380 |
| m^1^I | 19600 | 5880 |
| m^5^Cm | 68.6 | 20.6 |
| m^4,4^Cm | 274 | 82.3 |
| mchm^5^U | 100000 | 30000 |
| m^2,7^G | 41.2 | 12.4 |
| mcm^5^U | 317 | 95.0 |
| Gm | 27.4 | 8.23 |
| OHyW | 5.08 | 1.52 |
| yW | 1.41 | 0.423 |
| OHyWx | 0.0306 | 0.00918 |
| Im | 2.12 | 0.635 |
| Ar(P) | 0.635 | 0.191 |
| ms^2^io^6^A | 0.339 | 0.102 |
| tm^5^U | 3.63 | 1.09 |
| cmnm^5^U | 16.9 | 5.08 |
| Q | 10.2 | 3.05 |
| ncm^5^Um | 16.9 | 5.08 |
| ms^2^i^6^A | 926 | 381 |
| tm^5^s^2^U | 3090 | 926 |
| ms^2^t^6^A | 0.267 | 0.0802 |
| manQ | 16.9 | 5.08 |
| galQ | 16.9 | 5.08 |
| i^6^A | 0.318 | 0.0953 |
| ms^2^m^6^A | 0.254 | 0.0762 |
| cmo^5^U | 1.75 | 0.526 |
| m^1^acp^3^ψ | 374 | 112 |
| mcm^5^s^2^U | 2310 | 694 |
| chm^5^U | 5.08 | 1.52 |
| O^2^yW | 11100 | 3700 |
| m^6,6^Am | 337 | 41.2 |
| t^6^A | 1.69 | 0.191 |
| imG-14 | 1.02 | 0.305 |
| PreQ0 | 28.6 | 8.57 |
| m^6,6^A | 112 | 33.7 |
| ac^4^Cm | 61.7 | 18.5 |
| mcm^5^Um | 374 | 112 |
| m^2,2^G | 58.8 | 13.7 |
| m^5^Um | 1230 | 370 |
| m^3^Um | 514 | 103 |
| m^2,2,7^G | 4.16 | 1.25 |
| f^5^Cm | 142 | 19.5 |
| mcmo^5^U | 650 | 195 |
| ac^4^C | 112 | 33.7 |
| Am | 168 | 41.2 |

**Table S5.** Dynamic MRM parameters for ribonucleosides based on optimizer results.

| rN name^a^ | RT (min) | RT Window (min) | Precursor (m/z) | Product (m/z) | CE (V) | Min Dwell Time (ms) | RF Lens (V) |
| --- | --- | --- | --- | --- | --- | --- | --- |
| hm^5^C | 1.6 | 3 | 274 | 142 | 11 | 31.125 | 36 |
| m^6^C | 1.47 | 2 | 258 | 126 | 12 | 29.799 | 76 |
| ho^5^U | 1.50 | 2 | 261 | 129 | 10 | 29.799 | 57 |
| m^5^D | 1.80 | 2 | 261 | 129 | 10 | 29.799 | 57 |
| m^4^C | 1.92 | 2 | 258 | 126 | 12 | 29.799 | 76 |
| m^3^C | 1.97 | 2 | 258 | 126 | 12 | 29.799 | 76 |
| ψ | 2.00 | 4 | 245 | 209 | 9 | 29.799 | 34 |
| m^5^C | 2.27 | 2 | 258 | 126 | 12 | 29.799 | 76 |
| ca^5^C | 2.29 | 4 | 288 | 156 | 11 | 29.799 | 36 |
| s^2^ψ | 2.50 | 4 | 261 | 225 | 10 | 29.799 | 38 |
| ncm^5^U | 2.50 | 4 | 302 | 170 | 10 | 29.799 | 40 |
| hm^5^U | 2.51 | 3 | 275 | 125 | 14 | 29.799 | 47 |
| m^1^A | 2.51 | 3 | 282 | 150 | 18 | 29.799 | 53 |
| D | 2.60 | 4 | 247 | 115 | 9 | 29.799 | 55 |
| m^3^ψ | 2.60 | 5 | 259 | 223 | 10 | 29.799 | 39 |
| mnm^5^s^2^U | 2.81 | 4 | 304 | 141 | 18 | 29.799 | 42 |
| s^2^C | 3.00 | 3.5 | 260 | 128 | 12 | 29.799 | 37 |
| cm^5^U | 3.00 | 3 | 303 | 171 | 9 | 29.799 | 40 |
| m^1^ψ | 3.39 | 5 | 259 | 223 | 10 | 29.799 | 39 |
| m^5^U | 3.58 | 7 | 259 | 127 | 11 | 29.799 | 34 |
| Cm | 3.59 | 3 | 258 | 112 | 11 | 29.799 | 37 |
| Um | 4.00 | 4 | 259 | 113 | 8 | 29.799 | 46 |
| s^2^U | 4.12 | 7 | 261 | 129 | 10 | 29.799 | 57 |
| c^7^A | 4.14 | 8 | 267 | 135 | 19 | 29.799 | 56 |
| m^7^G | 4.40 | 6 | 298 | 166 | 14 | 29.799 | 39 |
| f^5^C | 4.63 | 4 | 272 | 140 | 11 | 29.799 | 33 |
| I | 4.71 | 4 | 269 | 137 | 11 | 29.799 | 40 |
| c^7^G | 4.74 | 6 | 283 | 151 | 18 | 29.799 | 57 |
| s^4^U | 4.76 | 7 | 261 | 129 | 10 | 29.799 | 57 |
| m^1^Am | 4.9 | 4 | 296 | 150 | 17 | 29.799 | 55 |
| m^3^U | 5.34 | 7 | 259 | 127 | 11 | 29.799 | 34 |
| m^2^A | 5.62 | 6 | 282 | 150 | 18 | 23.57 | 49 |
| mo^5^U | 5.66 | 4 | 275 | 143 | 11 | 29.799 | 36 |
| m^5^Cm | 6.73 | 9 | 272 | 126 | 13 | 28.576 | 37 |
| ^15^N^5^-dA | 7.00 | 8 | 257 | 141 | 14 | 28.576 | 38 |
| m^4,4^Cm | 7.26 | 4 | 286 | 140 | 14 | 44.846 | 40 |
| m^1^I | 7.98 | 6 | 283 | 151 | 18 | 29.799 | 57 |
| mchm^5^U | 8.39 | 4 | 333 | 187 | 18 | 39.851 | 72 |
| m^2,7^G | 8.68 | 4 | 312 | 180 | 14 | 34.117 | 45 |
| mcm^5^U | 8.70 | 3 | 317 | 185 | 9 | 42.208 | 38 |
| m^1^G | 9.27 | 7 | 298 | 166 | 17 | 23.485 | 56 |
| m^8^A | 10.30 | 6 | 282 | 106 | 54 | 23.57 | 47 |
| m^6^A | 10.44 | 6 | 282 | 94 | 56 | 23.57 | 41 |
| Gm | 10.15 | 6 | 298 | 152 | 12 | 23.537 | 36 |
| Am | 10.70 | 8 | 282 | 136 | 16 | 23.485 | 49 |
| m^2^G | 10.81 | 7 | 298 | 166 | 17 | 23.485 | 56 |
| ac^4^C | 11.15 | 6 | 286 | 154 | 9 | 23.485 | 31 |
| m^5^Um | 11.24 | 6 | 273 | 127 | 12 | 23.485 | 35 |
| mcmo^5^U | 11.28 | 6 | 333 | 201 | 9 | 23.485 | 41 |
| f^5^Cm | 11.46 | 5 | 286 | 140 | 11 | 23.485 | 36 |
| m^2,2,7^G | 11.72 | 5 | 326 | 194 | 15 | 23.485 | 48 |
| m^5^s^2^U | 12.00 | 9 | 275 | 143 | 10 | 23.485 | 36 |
| m^2,2^G | 12.02 | 5 | 312 | 180 | 22 | 23.485 | 48 |
| mcm^5^Um | 12.42 | 4 | 331 | 185 | 9 | 23.485 | 36 |
| m^6^Am | 12.46 | 5 | 296 | 150 | 17 | 23.485 | 53 |
| m^3^Um | 12.52 | 6 | 273 | 127 | 12 | 23.485 | 35 |
| ac^4^Cm | 12.54 | 4 | 300 | 154 | 10 | 23.485 | 36 |
| m^6,6^A | 12.56 | 4 | 296 | 164 | 21 | 23.485 | 63 |
| PreQ0 | 12.56 | 4 | 308 | 176 | 16 | 23.485 | 59 |
| m^6^G | 12.63 | 7 | 298 | 166 | 17 | 23.485 | 56 |
| imG-14 | 12.78 | 5 | 322 | 190 | 15 | 23.485 | 47 |
| t^6^A | 12.80 | 4 | 413 | 281 | 12 | 23.485 | 56 |
| m^6,6^Am | 12.94 | 4 | 310 | 164 | 19 | 23.485 | 64 |
| O^2^yW | 12.94 | 4 | 542 | 496 | 16 | 23.485 | 117 |
| chm^5^U | 13.10 | 6 | 319 | 278 | 8 | 23.485 | 67 |
| mcm^5^s^2^U | 13.20 | 5 | 333 | 169 | 19 | 23.485 | 46 |
| m^1^acp3ψ | 13.22 | 5 | 360 | 319 | 6 | 23.485 | 57 |
| cmo^5^U | 13.32 | 6 | 319 | 187 | 9 | 23.485 | 43 |
| ms^2^m^6^A | 13.42 | 4 | 328 | 196 | 21 | 23.485 | 63 |
| i^6^A | 13.46 | 4 | 336 | 204 | 16 | 23.485 | 63 |
| manQ | 13.47 | 4 | 572 | 526 | 16 | 23.485 | 97 |
| galQ | 13.58 | 4 | 572 | 526 | 16 | 23.485 | 97 |
| ms^2^t^6^A | 13.80 | 4 | 459 | 327 | 14 | 23.485 | 73 |
| tm^5^s^2^U | 13.90 | 4 | 398 | 263 | 13 | 23.485 | 66 |
| ms^2^i^6^A | 14.32 | 5 | 382 | 250 | 19 | 23.485 | 92 |
| ncm^5^Um | 14.36 | 5 | 361 | 279 | 14 | 23.485 | 81 |
| Q | 14.40 | 4 | 410 | 295 | 19 | 23.485 | 75 |
| cmnm^5^U | 15.94 | 5 | 322 | 125 | 18 | 23.485 | 41 |
| tm^5^U | 17.20 | 5 | 382 | 266 | 16 | 35.771 | 90 |
| ms^2^io^6^A | 17.80 | 5 | 398 | 377 | 5 | 47.783 | 72 |
| Ar(P) | 18.20 | 5 | 480 | 439 | 7 | 59.373 | 105 |
| Im | 18.72 | 5 | 283 | 247 | 11 | 77.699 | 49 |
| OHyWx | 20.40 | 6 | 453 | 433 | 5 | 112.008 | 80 |
| yW | 21.00 | 6 | 509 | 463 | 22 | 112.008 | 117 |
| OHyW | 21.70 | 6 | 525 | 465 | 22 | 112.02 | 120 |
